# Asymmetric Hydrogenation of Ketones by Simple Alkane-Diyl-Based Ir(P,N,O) Catalysts: A Comparative Study

**DOI:** 10.3390/molecules29163743

**Published:** 2024-08-07

**Authors:** Zsófia Császár, Mária Guóth, Margit Kovács, Attila C. Bényei, József Bakos, Gergely Farkas

**Affiliations:** 1 Research Group of Organic Chemistry—Synthesis and Catalysis, University of Pannonia, Egyetem u. 10, H-8200 Veszprém, Hungary; csaszar.zsofia@mk.uni-pannon.hu (Z.C.); guoth.marika@gmail.com (M.G.); 2 NMR Laboratory, University of Pannonia, Egyetem u. 10, H-8200 Veszprém, Hungary; kovacs.margit@mk.uni-pannon.hu; 3 Department of Physical Chemistry, University of Debrecen, Egyetem tér 1, H-4032 Debrecen, Hungary; benyei.attila@science.unideb.hu

**Keywords:** iridium, asymmetric hydrogenation, prochiral ketone, phosphine–amine–alcohol, phosphine–amine–ether, P,N,O ligand

## Abstract

The development of new chiral ligands with simple and modular structure represents a challenging direction in the design of efficient homogeneous transition metal catalysts. Herein, we report on the asymmetric hydrogenation of prochiral ketones catalyzed by the iridium complexes of simple alkane-diyl-based P,N,O-type chiral ligands with a highly modular structure. The role of (i) the P-N and N-O backbone in the potentially tridentate ligands, (ii) the number, position and relative configuration of their stereogenic elements and (iii) the effect of their NH and OH subunits on the activity and enantioselectivity of the catalytic reactions are studied. The systematic variation in the ligand structure and the comparative catalytic experiments shed light on different mechanistic aspects of the iridium-catalyzed reaction. The catalysts containing the simple alkane-diyl-based ligands with central chirality provided high enantioselectivities (up to 98% *ee*) under optimized reaction conditions and proved to be active and selective even at very high substrate concentrations (100 mmol substrate/mL solvent).

## 1. Introduction

Transition metal-catalyzed asymmetric hydrogenation of prochiral ketones is one of the most convenient and economically feasible methodologies for the synthesis of optically enriched chiral alcohols [1,2]. These products are valuable building blocks for a number of biologically active compounds such as pharmaceuticals, agrochemicals and fragrances [3,4,5]. Besides the high atom economy of using molecular hydrogen as a reactant in the catalytic process, the easy separation of the excess H_2_ gas further enhances the synthetic value of the methodology [6].

**Figure 1:**
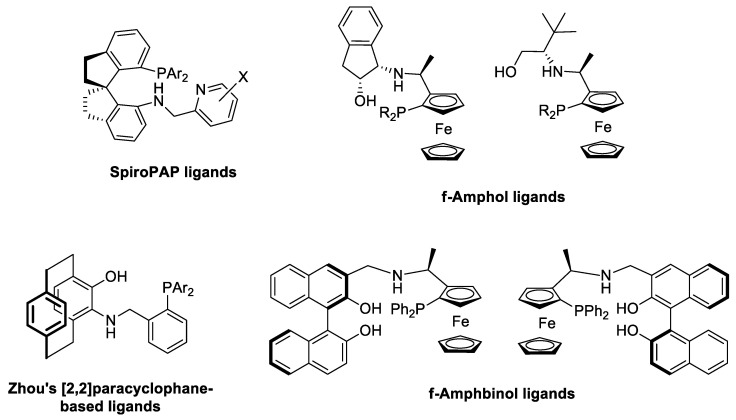
Multidentate ligands used in Ir-catalyzed asymmetric hydrogenation.

Since the development of the highly efficient [RuCl_2_(diphosphine)(diamine)]-type hydrogenation catalysts by Noyori and coworkers [7,8], significant research efforts have been devoted to the synthesis of even more efficient homogeneous catalysts for the asymmetric reduction of ketones [5,9]. In this contribution, iridium complexes modified by chiral tridentate ligands represent a unique class of catalysts due to their extremely high activity, enantioselectivity and stability [10]. The success of tridentate ligands compared to their bidentate analogues may rest upon the presence of an extra coordinating site that prevents the formation of catalytically inactive *bis*-ligated species and also leads to the formation of a conformationally less flexible chiral environment around the metal [11,12]. Furthermore, tridentate ligands have generally higher structural modularity compared to their bidentate analogues and therefore are more suitable for stereo-electronic fine-tuning [13]. The first highly efficient Ir catalyst containing a potentially tridentate P,N,N-type ligand, SpiroPAP, was developed by Zhou and coworkers (Figure 1) and was utilized in the asymmetric hydrogenation of a wide range of ketonic substrates, resulting in extremely high activities (turnover frequency > 100,000 h^−1^ in the hydrogenation of acetophenone) and enantioselectivities (>99% *ee*) [11]. Later, novel P,N,N-type ligand families emerged and proved to be efficient catalysts in iridium-catalyzed asymmetric hydrogenation [14]. Unlike P,N,N-type chiral stereoselectors, however, chiral phosphine–amine–alcohols or –phenols (P,N,OH ligands) represent a significantly less studied group of compounds despite their unique catalytic features [15,16].

Zhang and coworkers developed a ferrocene-based P,N,OH ligand family (f-Amphol) that was utilized in the iridium-catalyzed asymmetric hydrogenation of acetophenone derivatives (Figure 1) [17]. The catalysts afforded product alcohols with high enantioselectivities (98–99.9% *ee*) and activities, and the catalysts proved to be very stable in hydrogenation reactions (TON up to 200,000). A computational study on the catalytic intermediates provided firm evidence that the hydroxyl group plays a key role in the hydrogenation process. Later, Zhang’s f-Amphol ligand was utilized in the enantio- and diastereoselective hydrogenation of α-substituted β-ketoesters through dynamic kinetic resolution [18]. The products were obtained in high yields (up to 98%) with excellent diastereomeric ratios (up to 96:4) and enantioselectivities (>99 *ee*). Additionally, a member of the f-Amphol ligand family proved to be extremely efficient in the asymmetric hydrogenation of benzo-fused cyclic ketones, where *ee*’s up to 99% and TON up to 297,000 could be obtained [19].

Recently, Zhou et al. synthesized a P,N,OH ligand family based on a [2,2]-paracyclophane framework with planar chirality (Figure 1) [20]. The Ir-complexes of the ligands provided excellent enantioselectivities (up to >99 *ee*) in the asymmetric hydrogenation of simple alkyl-aryl ketones. The presence of both the NH and OH functionality in the ligand was crucial to achieve considerable catalytic activity and enantioselectivity.

Novel ferrocene-based aminophosphine-binol (f-Amphbinol)-type ligands were prepared by Zhou and Ji and were applied in the stereoselective hydrogenation of aryl-substituted cyclohexanones and in the asymmetric hydrogenation of simple alkyl-aryl ketones (Figure 1) [21]. High diastereoselectivities (>99:1) and enantioselectivities (83–99% *ee*) could be obtained in the iridium-catalyzed processes. Again, the indispensable role of both the NH and OH moieties was proved.

Besides the aforementioned ligand families, it is important to note that tridentate phosphine–amine–carboxylic acids (f-Ampha) [22] or potentially tetradentate P,N,N,O(H) ligands [23,24] are also extremely powerful stereoselectors in iridium-catalyzed asymmetric hydrogenation reactions that can also be utilized in the industrial-scale production of high-value bio-active compounds [25].

The above examples clearly demonstrate that the development of novel P,N,OH ligands represent a challenging new direction in catalysis research aiming at the improvement of catalytic efficiency and the identification of key structural elements in successful stereoelectronic communication between the catalysts and the substrate. However, ligand modifications in the literature generally follow the principle of changing the steric and/or electronic properties of the donor atoms by simply varying their substituents, which somewhat limits the potential of efficient ligand screening. Additionally, to the best of our knowledge, there is no literature report on the application of P,N,OH ligands in iridium-catalyzed asymmetric hydrogenation that have simple central chirality exclusively instead of planar or axially chiral subunits. The simplification of the ligand structure together with a highly modular synthetic approach would offer an outstanding opportunity for the efficient structural screening of an easily available chiral P,N,OH ligand family. Inspired by this philosophy in ligand design and the high potential of P,N,OH ligands in iridium-catalyzed asymmetric hydrogenation, we decided to develop an alkane-diyl-based phosphine–amine–alcohol ligand family. Our intention was to prove the usefulness of simple alkane-diyl-based ligands equipped with solely centrally chiral subunits in iridium-catalyzed asymmetric hydrogenation and the identification of the key structural elements of the ligands in the catalytic process.

## 2. Results and Discussion

### 2.1. Synthesis of the Novel Ligands

**Figure 2:**
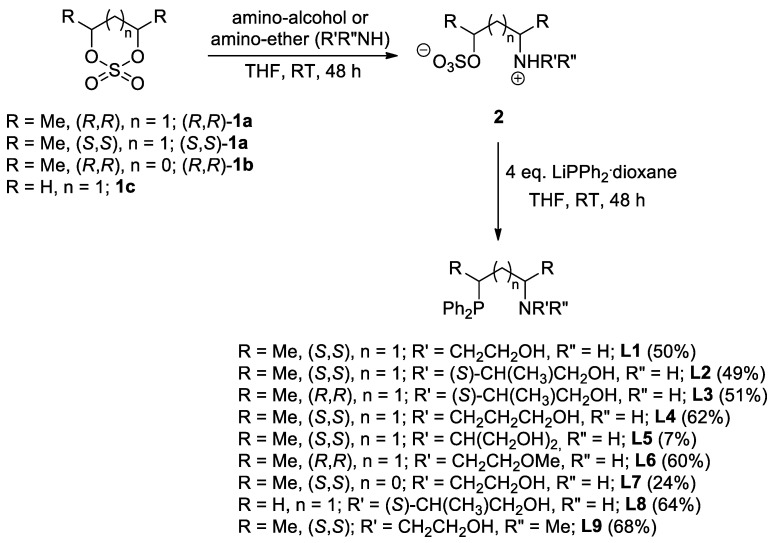
Synthesis of PNO ligands via ring opening of cyclic sulfates. (Values in parentheses represent the yield of the synthesis relative to the corresponding cyclic sulfate **1**).

**Figure 3:**
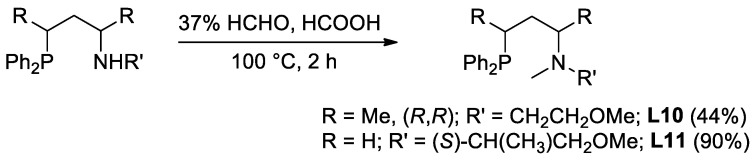
Synthesis of PNO ligands with tertiary amine functionality through Eschweiler–Clarke methylation. (Values in parentheses represent the yield of the synthesis).

Recently, we developed a synthetic protocol for the preparation of structurally analogous alkane-diyl-based chiral phosphine–amine–alcohol (**L1-3**, Figure 2) [26] and phosphine–amine–ether-type (**L6** and **L11**, Figure 2 and Figure 3, respectively) [27] ligands that were utilized in palladium-catalyzed asymmetric allylic alkylation and ruthenium-catalyzed asymmetric hydrogenation, respectively. In the present study, we broadened the scope of our P,N,O ligand library based on the same highly modular, two-step synthetic procedure (Figure 2). At first, chiral or achiral cyclic sulfates **1** were treated with aminoalcohols or aminoethers, resulting in the formation of the corresponding sulfated amines (**2**). The subsequent reaction of compounds **2** with lithium diphenylphosphide afforded the desired novel tridentate ligands **L4**, **L5** and **L7–L9**. In the case of chiral starting materials (*R*,*R*)- or (*S*,*S*)-**1a** and (*R*,*R*)-**1b**, both steps occurred with complete inversion at the stereogenic centers, resulting in the stereoselective formation of the product.

In addition, ligand **L10** could selectively be synthesized through Eschweiler–Clark methylation [28,29] of the parent compounds **L6** with secondary amine functionality, respectively (Figure 3).

**Figure 4:**
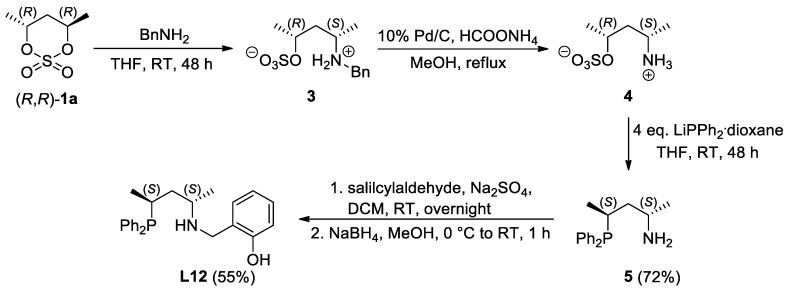
Multistep synthesis of ligand **L12** starting from cyclic sulfate (*R*,*R*)-**1a**. (Yield of **5** is calculated relative to **3** and yield of **L12** is calculated relative to **5**).

Unlike ligands **L1**–**11** with an aliphatic N-O backbone, compound **L12** containing an *o*-hydroxybenzyl moiety was prepared by the functionalization of the corresponding primary aminoalkyl-phosphine **5** (Figure 4). The preparation of this intermediate was realized by the slight modification of the procedure starting from cyclic sulfates, according to Figure 4. The ring opening of (*R*,*R*)-**1a** with benzyl amine delivered the *N*-benzyl-protected salt **3** [30] that can be converted to the primary amine **5** by the removal of the protecting group under hydrogenative conditions and by a subsequent nucleophilic substitution step.

With the novel ligand library in hand, we were able to investigate the effect of (i) the P-N and N-O backbone of the potentially tridentate ligands, (ii) the relative configuration of their stereogenic elements and (iii) the N-R and O-R (R = H or Me) subunits on the activity and selectivity of iridium-catalyzed asymmetric hydrogenation.

### 2.2. Asymmetric Hydrogenation

In the first set of experiments, we set out to investigate the effect of the reaction conditions using ligand **L1** as the stereoselector. The precatalyst was generated in situ starting from the commercially available [Ir(COD)Cl]_2_ and two equivalents of the chiral ligand. Initially, the reaction was carried out at a substrate/catalyst (S/C) molar ratio of 500 and base/catalyst (B/C) ratio of 5 in isopropanol as solvent under 30 bar hydrogen pressure at room temperature. The variation in the base cation and the B/C ratio strongly affected the activity and selectivity of the catalyst (entries 1–5, Table 1). The highest *ee* (92%, entry 3) could be obtained with lithium-*tert*butoxyde at a B/C ratio of 10. Next, the effect of the solvent was investigated (entries 6–11), which proved to be crucial in terms of activity and selectivity. The reaction proceeds smoothly in alcohols (entries 6–8), but polar non-protic solvents have a detrimental effect on both conversion and enantioselectivity (9–10). The highest *ee*’s, 94 and 95%, could be observed in ethanol and *n*-butanol, respectively. Furthermore, the reaction could also be realized in 96% ethanol (entry 11) or under anhydrous conditions at S/C = 1000, with 95% enantioselectivity and complete conversion (entry 12).

**Figure 5:**
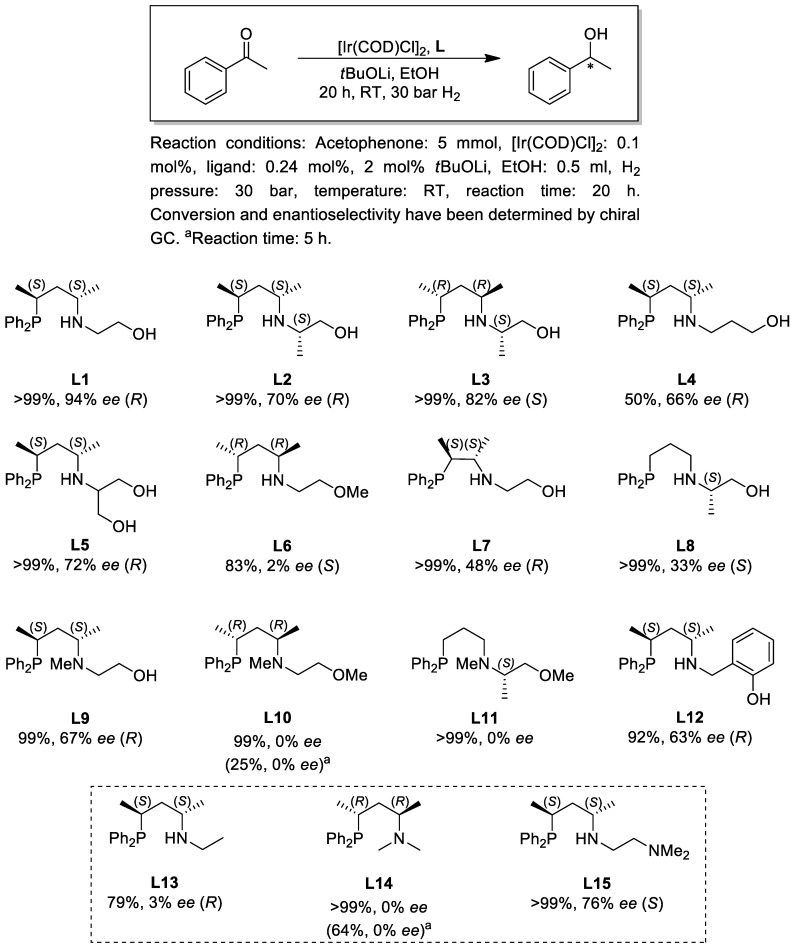
Asymmetric hydrogenation of acetophenone with different chiral ligands.

Next, we investigated the ligand effects in the asymmetric hydrogenation reaction of the benchmark substrate acetophenone. The reactions were performed at a S/C = 500 and B/C = 10, in dry ethanol under 30 bar hydrogen pressure using *t*BuOLi as a base. Similarly to the previous experiments, the catalyst was synthesized in situ by mixing the dimer [Ir(COD)Cl]_2_ with two equivalents of the chiral ligand. In addition to phosphine–amine–alcohol (P,N,OH) ligands **L1–5**, **L7–9** and **L12**, the reaction was also performed in the presence of phosphine–amine–ethers **L6**, **L10** and **L11** (Figure 5). Based on the catalytic results achieved, some general conclusions can be drawn. First, under the reaction conditions applied all catalysts proved to be catalytically active, although significant differences can be observed in enantioselectivity. Second, catalysts modified with pentane-diyl-based phosphine–amine–alcohols (**L1–5**, **L9** and **L12**) provided the product with generally higher enantioselectivity than with the rest of the ligands. Third, in the case of phosphine–amine–alcohols (**L1–5**, **L9** and **L12**) the configuration of the main product enantiomer is determined by the configuration of the P-N-framework, irrespective of the N-O-backbone: ligands with (*S*,*S*)-pentane-2,4-diyl backbone provided (*R*)-1-phenylethanol as the main product, and **L3** with (*R*,*R*)-configuration afforded the prevailing product enantiomer with (*S*)-configuration.

The enantioselectivities achieved by ligands **L1-3** clearly prove that the introduction of a stereogenic element into the N-O tether has a detrimental effect on the enantioselectivity. Although this result might seem somewhat unexpected, the addition of a new chirality element does not necessarily improve selectivity [31]. Similarly, further modification of the N-O bridge (**L4**, **L5** and **L12**) led to reduced selectivity of the catalytic reaction compared to **L1**.

The comparison of the catalytic results obtained by ligands **L1**, **L6**, **L9** and **L10**, which differ from each other only in the number and position of methyl substituents, suggests that the presence of the OH functionality is essential to achieve enantioselectivity. Although the catalyst modified by the N-Me-containing **L9** provided lower enantioselectivity (67% *ee*) compared to **L1** (94% *ee*), it is still a significantly better stereoselector than its analogues **L6** (2% *ee*) or **L10** (0% *ee*) without the OH site. This observation reflects the pivotal role of the OH group in the catalytic process.

To interpret the function of the OH group in the catalytic reaction, we performed further catalytic tests using simple alkane-diyl-based bidentate systems **L13** [32] and **L14** [33] under identical conditions. Interestingly, the enantioselectivities and activities achieved by the Ir-complexes of bidentate ligands **L13** (79%, 3% *ee*) and **L14** (64%, 0% *ee*) are very similar to those obtained by the catalysts bearing the O-methylated ligands **L6** (83%, 2% *ee*) and **L10** (25%, 0% *ee*). Based on these results and literature findings [34], it is reasonable to assume that in the O-methylated compounds the N-O-framework remains a pendant side arm as the ligands coordinate in a κ^2^-P,N bidentate fashion to iridium in the catalytically active species mainly due to steric reasons. In contrast to this, a κ^3^-P,N,O coordination pattern is expected for P,N,OH ligands [17,20,25]. The remarkably different coordination mode can explain the differences in the enantioselectivities obtained by phosphine–amine–ethers **L6** and **L10** and phosphine–amine–alcohols **L1** and **L9**, respectively.

Furthermore, the crucial role of the OH functionality is also manifested in the significantly different catalytic results achieved by ligands **L1** and **L15**. The PNN-type ligand **L15** [35] is an analogue of **L1**, both having the same (*S*,*S*)-pentane-2,4-diyl backbone as the only source of chirality. Despite their close structural analogy, however, the catalyst modified by **L15** produced (*S*)-1-phenylethanol with 76% *ee* in contrast to **L1** which leads to the formation of (*R*)-1-phenylethanol with 94% *ee*. The observed enantioselectivity switch can be explained in terms of a metal–ligand bifunctional mechanism where a protic subunit (e.g., NH) of the chiral ligand is involved in the stabilization of the hydrogenation transition state through the formation of a hydrogen bonding interaction (e.g., NH^…^O=C) [36]. This interaction also strongly influences the enantiodiscrimination of the catalytic reaction through affecting the orientation of the substrate to the catalyst. As ligand **L15** has no OH, the hydrogenation necessarily involves the cooperation of its internal amino site. For **L1**, however, it is surmised that the catalytic process is promoted by its O-containing moiety instead of the amino group. The different orientation of the substrate in the two cases may be responsible for the opposite selectivities of the two systems. Obviously, it cannot be excluded that conformational differences or the hemilability of the PNN ligand [37] also play an important role in the distinct catalytic behavior. Nevertheless, the catalytic results obtained by **L1** and **L15** emphasize the fact that an apparently minor modification in the ligand structure, i.e., the introduction of a competing protic functionality (e.g., OH site) into a chiral tridentate ligand, represents a useful tool to reverse enantioselectivity without changing the source of chirality in the catalyst.

Finally, the variation in the P-N-backbone, i.e., changing the pentane-2,4-diyl moiety to butane-2,3-diyl (**L7**) or propane-1,3-diyl unit (**L8**), reduced the enantioselectivity, although complete conversions were obtained. Again, **L11**, the N- and O-methylated derivative of **L8** afforded a racemic product, similarly to **L10** and **L14**.

**Figure 6:**
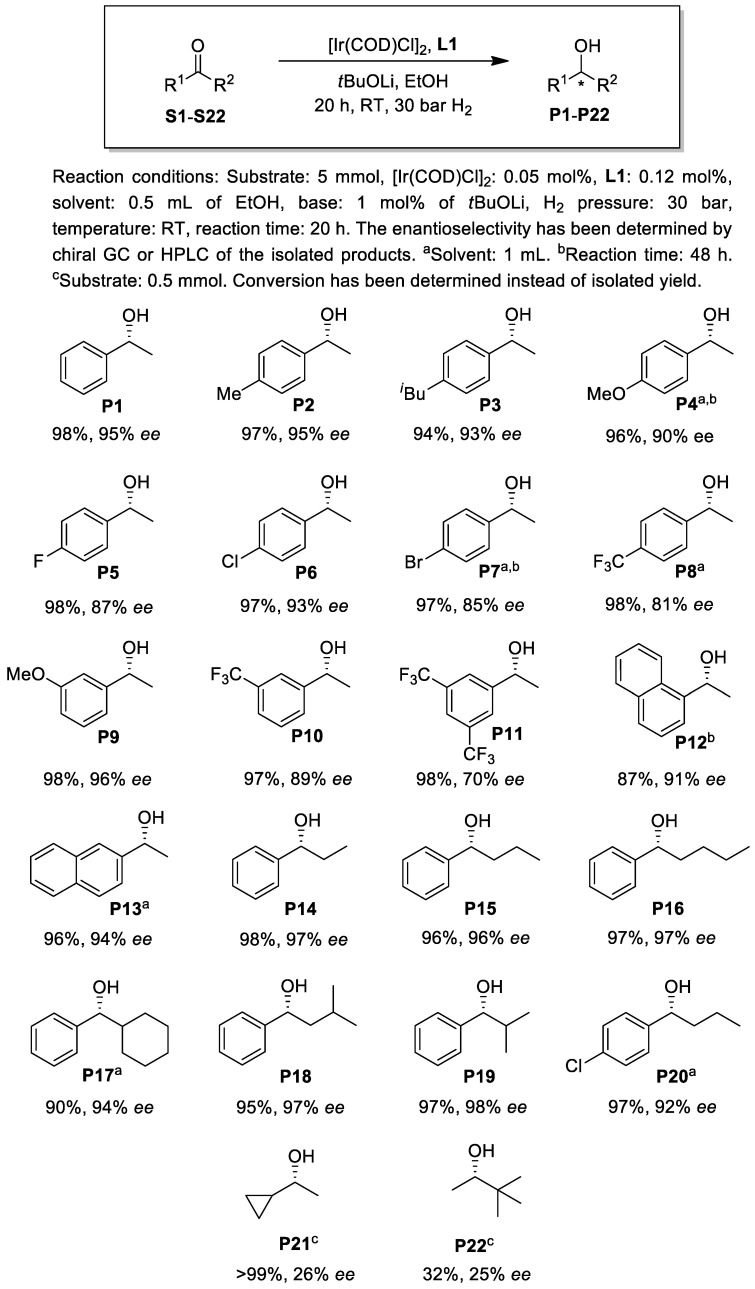
Substrate screening in the Ir-catalyzed asymmetric hydrogenation using chiral ligand **L1**.

Motivated by the high enantioselectivity achieved by **L1**, we decided to broaden the scope of the ketonic substrates. A variety of aromatic ketones were hydrogenated under highly concentrated conditions at a substrate/catalyst molar ratio of 1000 (Figure 6). Although alcohols can be considered green reaction media, the minimization of their use as solvents is also important as it can help to prevent the generation of hazardous waste [38,39,40]. Additionally, the reduction in the amount of solvent in chemical syntheses may lead to more energy-efficient technologies, increased reaction rates and reduced batch sizes. Although there are several literature examples for the hydrogenation of ketones at elevated substrate concentrations (>10 mmol substrate/mL solvent) in high S/C molar ratio experiments, e.g., [11,19,22,25,41,42,43,44], substrate screening with Ir(PNN)- or Ir(PNO)-based simple ketone hydrogenation catalysts is generally performed at a concentration range of 0.2–3 mmol substrate/mL solvent. In the present study, the substrate screening tests for **S1–20** were conducted at concentrations of 5 or 10 mmol substrate/mL solvent depending on the solubility of the substrate at room temperature in ethanol (Figure 6).

To our delight, the catalyst afforded the product (**P1**--**P20**) of the hydrogenation reaction in high yields and with good to excellent enantioselectivities under the highly concentrated conditions. The hydrogenation of substrates with electron-donating aryl substituents led to generally higher enantioselectivities (**S2--S4**, **S9**) compared to those with electron-withdrawing groups (**S5--S8**, **S10** and **S11**). It is important to note that ketones with higher alkyl carbonyl substituents (**S14--S19**) gave excellent enantioselectivities (up to 98% *ee*) under the concentrated conditions. In contrast, the hydrogenation of dialkyl ketones **S21** and **S22** provided low optical yields.

In order to explore the limits of the catalyst, we decided to further increase the concentration of the substrate. In our catalytic experiments, the concentration could be increased to 100 mmol/mL ethanol at substrate/catalyst molar ratio of 500 with complete conversion after 20 h (Table 2). Additionally, only a minor decrease in enantioselectivity could be observed under these conditions. Considering the amount of the solvent (50 μL) relative to the substrate (585 μL acetophenone), ethanol can be regarded as an additive rather than a solvent.

**Figure 7:**
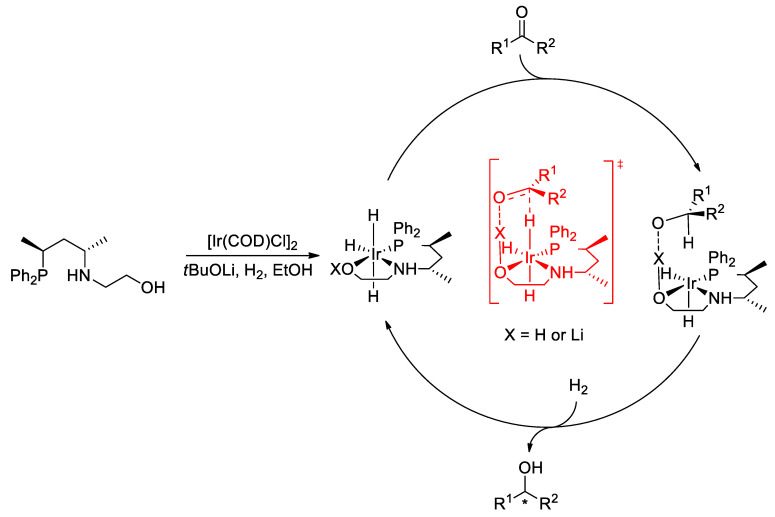
Proposed mechanism for the asymmetric hydrogenation catalyzed by Ir/**L1**.

Based on the outcome of the comparative experiments and literature findings, we proposed a mechanism for the hydrogenation of ketonic substrates (Figure 7). The catalytically active trihydrido-Ir(III) complexes are formed in the reaction between the chiral ligand and the Ir precursor in the presence of a base under hydrogen pressure [12,17,20]. As the acidity of the coordinated OH (X = H) is increased, due to its coordination to the transition metal, the hydroxyl group can be deprotonated by the excess base under hydrogenation conditions (X = Li) [45]. In this scenario, a C=O^…^Li-type electrostatic interaction links the substrate and the catalysts and can be responsible for stabilizing the transition state [18,46]. The transfer of the hydride from the Ir-H unit to the substrate and the subsequent reaction of the complex with H_2_ regenerate the catalytically active species.

**Figure 8:**
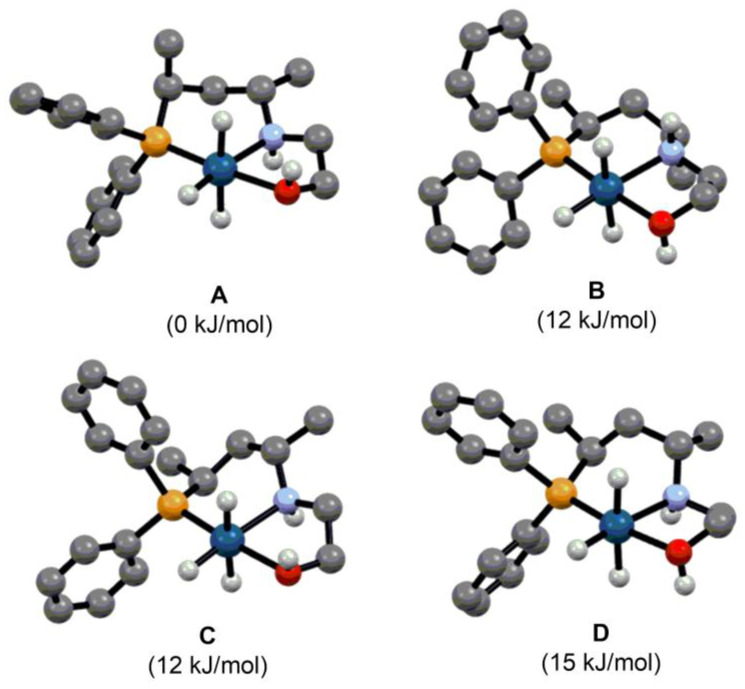
Possible stereoisomers of [IrH_3_(**L1**)] and their relative enthalpies. Atom colors: grey C, white H, orange P, blue N, red O, cyan Ir. The C-H hydrogens are omitted for clarity. (The conformation of the six- and five-membered ring and the configuration of nitrogen is chair, λ-skew and (*R*) in (**A**), chair, δ-skew and (*S*) in (**B**), twist, λ-skew and (*R*) in (**C**) and twist, δ-skew and (*R*) in (**D**)).

In order to shed light on the structural features of the catalytically active species, we performed density functional theory geometry optimizations for complex [IrH_3_(**L1**)] using the CAM-B3LYP functional combination with the SDD basis set and pseudopotential for Ir as well as the 6–31G* basis set for the rest of the atoms. Considering the possible conformations of the six-(chair or twist) and five-membered ring (δ- or λ-skew) and the two possible configurations of the stereogenic nitrogen ((*R*) or (*S*)) in the pincer-type complex, the structure of eight different stereoisomers were optimized (Table S1). The DFT calculations showed that in the lowest energy structure the six-membered ring adopts a chair conformation with axially and equatorially disposed methyl substituents moving from the phosphorus towards the nitrogen along the carbon backbone (Figure 8). The five-membered ring is stabilized in a λ-skew conformation and the stereogenic N-atom is fixed in (*R*)-configuration. As the lowest energy isomer is at least 12 kJ/mol more stable compared to the other optimized structures, one would presume that the formation of the complex occurs stereoselectively. Although ligands with a simple alkane-diyl backbone are often considered “stereolabile” systems, our study clearly shows that these types of compounds are capable of stereoselective coordination, which plays a key role in efficient chirality transfer.

## 3. Materials and Methods

All manipulations were carried out under argon using Schlenk techniques. Solvents were purified, dried, and deoxygenated by standard methods. Compounds **L1–3** [26], **L6**, **L11** [27], **L13** [32], **L14** [33], **L15** [35] and **3** [30] were prepared according to the reported literature method. All other starting materials were purchased from Sigma Aldrich and used without further purification. ^31^P{^1^H}-, ^13^C{^1^H}- and ^1^H-NMR measurements were conducted on a Bruker Avance 400 spectrometer (NMR Laboratory, University of Pannonia) operating at 161.98, 100.61 and 400.13 MHz, respectively. HR-MS experiments were carried out on a maXis II UHR ESI-QTOF mass spectrometer (Bruker, Bremen, Germany). A CE instrument (7100 model, Santa Clara, CA, USA) was used to inject and transport the samples. otofControl (version 4.1, build: 3.5, Bruker) controlled the MS instrument. Compass DataAnalysis (version: 4.4, build: 200.55.2969, Bruker, Billerica, MA, USA) was used to evaluate the data. Gas chromatography analysis was performed using an Agilent Technologies 6850 instrument; HPLC analysis was carried out using a Hewlett Packard Series 1050 instrument. Details of the analysis are available in the Supplementary Material.

### 3.1. Synthesis and Characterization of the New Compounds

#### 3.1.1. Synthesis of 3-(((2*S*,4*S*)-4-(diphenylphosphino)pentan-2-yl)amino)propan-1-ol (**L4**)

3-Amino-propane-1-ol (347 μL, 4.53 mmol) was added to the solution of (4*R*,5*R*)-4,5-dimethyl-1,3,2-dioxathiolane 2,2-dioxide ((*R*,*R*)-**1a**) (750 mg, 4.53 mmol) in THF (5 mL) and the mixture was stirred for 48 h at room temperature. The volume of the solvent was reduced to ca. 2 mL in vacuum and ether (20 mL) was added. A precipitate formed and the mixture was stirred for 30 min. The supernatant was decanted, and the solvent residue was removed in vacuum. In a separate flask, LiPPh_2_.1,4-dioxane adduct (5 g, 18.12 mmol) was dissolved in THF (35 mL) under argon, and the solution was cooled to 0 °C and then transferred carefully to sulfated amine via cannula under argon. The reaction mixture was stirred for 48 h at room temperature. The color of the reaction mixture remained red after stirring. After evaporating the solvent, deoxygenated water (50 mL) and ether (35 mL) were added to the residue and the mixture was stirred until the two phases became clear solutions. The pH of the mixture was then adjusted to 1 with 10% deoxygenated HCl solution. The two phases were then separated, and the water phase was washed three times with 35 mL portions of ether. The pH was then adjusted to around 9–10 with the dropwise addition of a dilute solution of Na_2_CO_3_. The product was extracted four times with 35 mL portions of ether. After drying with MgSO_4_ the solvent was evaporated. The crude product mixture was purified by column chromatography (silica gel, eluent: CHCl_3_/MeOH 4/1) to give 3-(((2*S*,4*S*)-4-(diphenylphosphino)pentan-2-yl)amino)propan-1-ol (**L4**) as a white solid. Yield: 930 mg, 62%. ^1^H NMR (400 MHz, Acetone-d_6_) δ 7.62–7.49 (m, 4H, aromatic), 7.42–7.31 (m, 6H, aromatic), 3.65–3.57 (m, 2H), 2.81–2.68 (m, 2H), 2.69–2.52 (m, 2H), 1.62–1.55 (m, 2H), 1.53–1.38 (m, 1H, diast. C*H*H), 1.38–1.23 (m, 1H, diast. CH*H*), 1.02 (dd, *J* = 15.1, 6.6 Hz, 3H, C*H*_3_CHP), 1.00 (d, *J* = 6.5 Hz, 3H, C*H*_3_CHN). ^31^P NMR (162 MHz, Acetone-d_6_) δ 2.14 (s). ^13^C NMR (101 MHz, Acetone-d_6_) δ 137.76 (d, *J* = 15.2 Hz, 1C, aromatic), 137.60 (d, *J* = 16.1 Hz, 1C, aromatic), 133.71 (d, *J* = 19.6 Hz, 2C, aromatic), 133.52 (d, *J* = 19.1 Hz, 2C, aromatic), 128.74 (s, 1C, aromatic), 128.68 (s, 1C, aromatic), 128.32 (d, *J* = 6.9 Hz, 2C, aromatic), 128.28 (d, *J* = 7.1 Hz, 2C, aromatic), 61.91 (s, 1C), 51.10 (d, *J* = 12.3 Hz, 1C), 45.60 (s, 1C), 41.00 (d, *J* = 17.8 Hz, 1C), 32.47 (s, 1C), 26.75 (d, *J* = 9.8 Hz, 1C), 19.41 (s, 1C), 16.14 (d, *J* = 16.1 Hz, 1C). HR-MS (ESI) calcd. for C_20_H_29_NOP [M + H]^+^: 330.1981; found: 330.1977.

#### 3.1.2. Synthesis of 2-(((2*S*,4*S*)-4-(diphenylphosphino)pentan-2-yl)amino)propane-1,3-diol (**L5**)

Ligand **L5** was prepared according to the procedure described for **L4**. Greasy solid. Yield: 7%. ^1^H NMR (400 MHz, MeOD) δ 7.60–7.50 (m, 4H, aromatic), 7.39–7.31 (m, 6H, aromatic), 3.64 (ddd, *J* = 11.3, 5.2, 1.3 Hz, 2H, diast. CH_2_), 3.57 (ddd, *J* = 11.4, 5.5, 1.0 Hz, 2H, diast. CH_2_), 3.30–3.21 (m, 1H, CH), 2.98 (p, *J* = 5.3 Hz, 1H, CH), 2.66–2.40 (m, 1H, CH), 1.70–1.55 (m, 1H, diast. C*H*H), 1.53–1.37 (m, 1H, diast. CH*H*), 1.16 (d, *J* = 6.3 Hz, 3H, C*H*_3_CHN), 1.06 (dd, *J* = 15.2, 6.8 Hz, 3H, C*H*_3_CHP). ^31^P NMR (162 MHz, MeOD) δ 1.96 (s). ^13^C NMR (101 MHz, MeOD) δ 138.19 (d, *J* = 13.7 Hz, 1C, aromatic), 138.02 (d, *J* = 14.0 Hz, 1C, aromatic), 134.83 (d, *J* = 19.6 Hz, 2C, aromatic), 134.74 (d, *J* = 19.8 Hz, 2C, aromatic), 130.14 (s, 1C, aromatic), 130.09 (s, 1C, aromatic), 129.56 (d, *J* = 7.0 Hz, 2C, aromatic), 129.49 (d, *J* = 7.0 Hz, 2C, aromatic), 61.42 (s, 1C), 60.91 (s, 1C), 59.13 (s, 1C), 50.63 (d, *J* = 13.1 Hz, 1C), 40.10 (d, *J* = 17.6 Hz, 1C), 28.01 (d, *J* = 9.2 Hz, 1C), 18.40 (s, 1C), 16.33 (d, *J* = 16.6 Hz, 1C). HR-MS (ESI) calcd. for C_20_H_29_NO_2_P [M + H]^+^: 346.1930; found: 346.1928.

#### 3.1.3. Synthesis of 2-(((2*S*,3*S*)-3-(diphenylphosphino)butan-2-yl)amino)ethanol (**L7**)

Ligand **L7** was prepared according to the procedure described for **L4**. Transparent oil. Yield: 24%. ^1^H NMR (400 MHz, Acetone-d_6_) δ 7.68–7.55 (m, 4H), 7.42–7.25 (m, 6H), 3.53–3.49 (m, 2H, CH_2_), 2.87–2.78 (m, 1H, CH, partially overlapped with the very broad NH or OH signal), 2.77–2.57 (m, 3H, CH and CH_2_, overlapped with the very broad OH or NH signal), 1.11 (d, *J* = 6.5 Hz, 3H, CH_3_CHN), 0.93 (dd, *J* = 13.9, 7.0 Hz, 3H, CH_3_CHP). ^31^P NMR (162 MHz, Acetone-d_6_) δ −6.24 (s). ^13^C NMR (101 MHz, Acetone-d_6_) δ 137.85 (d, *J* = 14.9 Hz, 1C, aromatic), 137.75 (d, *J* = 15.2 Hz, 1C, aromatic), 133.85 (d, *J* = 20.8 Hz, 2C, aromatic), 133.39 (d, *J* = 20.1 Hz, 2C, aromatic), 128.79 (s, 1C, aromatic), 128.76 (s, 1C, aromatic), 128.42 (d, *J* = 7.1 Hz, 2C, aromatic), 128.28 (d, *J* = 7.5 Hz, 2C, aromatic), 60.96 (s, 1C), 53.24 (d, *J* = 17.4 Hz, 1C), 49.07 (s, 1C), 33.94 (d, *J* = 11.1 Hz, 1C), 15.71 (d, *J* = 10.0 Hz, 1C), 9.52 (d, *J* = 16.9 Hz, 1C). HR-MS (ESI) calcd. for C_18_H_25_NOP [M + H]^+^: 302.1668; found: 302.1663.

#### 3.1.4. Synthesis of (*S*)-2-((3-(diphenylphosphino)propyl)amino)propan-1-ol (**L8**)

Ligand **L8** was prepared according to the procedure described for **L4**. White solid. Yield: 64%. ^1^H NMR (400 MHz, Acetone-d_6_) δ 7.48–7.41 (m, 4H, aromatic), 7.39–7.30 (m, 6H, aromatic), 3.48–3.35 (m, 1H, diast. C*H*H), 3.24 (dd, *J* = 10.4, 6.8 Hz, 1H, diast. CH*H*), 2.77 (dt, *J* = 11.4, 7.0 Hz, 1H, CH), 2.72–2.58 (m, 2H, CH_2_), 2.15 (ddd, *J* = 7.6, 6.5, 1.6 Hz, 2H, CH_2_), 1.65–1.48 (m, 2H, CH_2_), 0.95 (d, *J* = 6.4 Hz, 3H, CH_3_). ^31^P NMR (162 MHz, Acetone-d_6_) δ −13.61 (s). ^13^C NMR (101 MHz, Acetone-d_6_) δ 140.15 (d, *J* = 14.2 Hz, 1C, aromatic), 140.11 (d, *J* = 14.2 Hz, 1C, aromatic), 133.46 (d, *J* = 18.7 Hz, 2C, aromatic), 133.44 (d, *J* = 18.7 Hz, 2C, aromatic), 129.34 (s, 2C, aromatic), 129.29 (d, *J* = 6.6 Hz, 4C, aromatic), 66.55 (s, 1C), 55.53 (s, 1C), 48.72 (d, *J* = 13.6 Hz, 1C, CH_2_), 27.81 (d, *J* = 16.2 Hz, 1C, CH_2_), 26.10 (d, *J* = 11.4 Hz, 1C, CH_2_), 17.57 (s, 1C, CH_3_). HR-MS (ESI) calcd. for C_18_H_25_NOP [M + H]^+^: 302.1668; found: 302.1667.

#### 3.1.5. Synthesis of 2-(((2*S*,4*S*)-4-(diphenylphosphino)pentan-2-yl)(methyl)amino)ethanol (**L9**)

Ligand **L9** was prepared according to the procedure described for **L4**. Transparent oil. Yield: 68%. ^1^H NMR (400 MHz, Acetone-d_6_) δ 7.60–7.51 (m, 4H, aromatic), 7.42–7.30 (m, 6H, aromatic), 3.48 (t, *J* = 5.9 Hz, 2H, CH_2_), 2.90–2.80 (m, 1H, CH), 2.63–2.51 (m, 1H, CH), 2.50–2.41 (m, 2H, CH_2_), 2.08 (s, 3H, NCH_3_), 1.56–1.45 (m, 1H, diast. C*H*H), 1.44–1.32 (m, 1H, diast. CH*H*), 1.02 (dd, *J* = 14.2, 6.9 Hz, 3H, C*H*_3_CHP), 0.91 (d, *J* = 6.6 Hz, 3H, C*H*_3_CHN). ^31^P NMR (162 MHz, Acetone-d_6_) δ 1.65 (s). ^13^C NMR (101 MHz, Acetone-d_6_) δ 138.71 (d, *J* = 14.9 Hz, 1C, aromatic), 138.44 (d, *J* = 16.3 Hz, 1C, aromatic), 134.68 (d, *J* = 19.9 Hz, 2C, aromatic), 134.23 (d, *J* = 19.0 Hz, 2C, aromatic), 129.65 (s, 1C, aromatic), 129.46 (s, 1C, aromatic), 129.21 (d, *J* = 4.8 Hz, 2C, aromatic), 129.14 (d, *J* = 5.2 Hz, 2C, aromatic), 59.83 (s, 1C), 57.09 (d, *J* = 11.0 Hz, 1C), 56.23 (s, 1C), 37.83 (d, *J* = 17.0 Hz, 1C), 36.45 (s, 1C), 27.70 (d, *J* = 10.2 Hz, 1C), 17.11 (d, *J* = 15.1 Hz, 1C), 14.06 (s, 1C). HR-MS (ESI) calcd. for C_20_H_29_NOP [M + H]^+^: 330.1981; found: 330.1981.

#### 3.1.6. Synthesis of (2*R*,4*R*)-4-(diphenylphosphino)-*N*-(2-methoxyethyl)-*N*-methylpentan-2-amine (**L10**)

In a small Schlenk tube, chiral ligand **L6** (300 mg, 0.91 mmol) was added to a mixture of HCOOH (138 µL, 3.64 mmol) and 412 µL of aqueous HCHO solution (37 wt%). The mixture was then stirred for 2 h at 100 °C. After being stirred, the solution was cooled to room temperature and 5 mL of 20% aqueous solution of NaOH was added. The product was extracted with 3 × 5 mL of diethyl ether and the combined organic phases were washed with water (2 × 5 mL) and dried over MgSO_4_. The solution was filtered and the solvent was evaporated in vacuum to give 140 mg of ligand **L10** as a transparent oil. Yield: 44%. ^1^H NMR (400 MHz, Acetone-d_6_) δ 7.65–7.48 (m, 4H), 7.45–7.28 (m, 6H), 3.37 (t, *J* = 6.2 Hz, 2H, CH_2_), 3.24 (s, 3H, OCH_3_), 2.85–2.75 (m, 1H, CHN), 2.63–2.54 (m, 1H, CHP), 2.54–2.37 (m, 2H, CH_2_), 2.12 (s, 3H, NCH_3_), 1.52–1.28 (m, 2H, CH_2_), 1.01 (dd, *J* = 14.1, 6.9 Hz, 3H, C*H*_3_CHP), 0.88 (d, *J* = 6.6 Hz, 3H, C*H*_3_CHN). ^31^P NMR (162 MHz, Acetone-d_6_) δ 2.83 (s). ^13^C NMR (101 MHz, Acetone-d_6_) δ 138.91 (d, *J* = 15.6 Hz, 1C, aromatic), 138.48 (d, *J* = 16.6 Hz, 1C, aromatic), 134.74 (d, *J* = 19.8 Hz, 2C, aromatic), 134.23 (d, *J* = 18.9 Hz, 2C, aromatic), 129.61 (s, 1C, aromatic), 129.37 (s, 1C, aromatic), 129.16 (d, *J* = 6.5 Hz, 2C, aromatic), 129.13 (d, *J* = 7.1 Hz, 2C, aromatic), 72.75 (s, 1C), 58.65 (s, 1C), 57.28 (d, *J* = 11.4 Hz, 1C), 53.36 (s, 1C), 38.17 (d, *J* = 17.8 Hz, 1C), 37.59 (s, 1C), 27.61 (d, *J* = 10.6 Hz, 1C), 17.37 (d, *J* = 14.5 Hz, 1C), 13.83 (s, 1C). HR-MS (ESI) calcd. for C_21_H_31_NOP [M + H]^+^: 344.2138; found: 344.2136.

#### 3.1.7. Synthesis of (2*S*,4*S*)-4-(diphenylphosphino)pentan-2-amine (**5**)

To a mixture of (2*R*,4*S*)-4-(benzylammonio)pentan-2-yl sulfate (**4**) (1.09 g, 4 mmol), ammonium formate (1.26 g, 20 mmol) and 29 mg of 10% Pd/C methanol (25 mL) was added and the resulting mixture was refluxed for 4 h. The reaction mixture was then cooled to room temperature and filtered through a short pad of celite. The solvent was removed from the filtrate in vacuum and the remaining solid was used directly in the next step without further purification. LiPPh_2_.1,4-dioxane adduct (4.9 g, 16 mmol) was dissolved in THF (35 mL) under argon and the solution was cooled to 0 °C. The crude product of the previous reaction was added to the red solution of the phosphide in small portions. The reaction mixture was stirred at room temperature for 48 h. The color of the reaction mixture remained red. After evaporation of the solvent, deoxygenated water (50 mL) and ether (35 mL) were added to the residue and the mixture was stirred until the two phases became clear solutions. The pH of the mixture was then adjusted to approximately 1 with 10% deoxygenated HCl solution. The two phases were then separated, and the water phase was washed three times with 35 mL portions of ether. The pH was then adjusted to around 9–10 with dropwise addition of a solution of Na_2_CO_3_. The product was then extracted four times with 35 mL portions of ether. After drying with MgSO_4_, the solvent was evaporated. The crude product mixture was subjected to column chromatography (silica gel, eluent: CHCl_3_/MeOH 4/1) to give the title compound (**5**) as a yellowish oil. Yield: 781 mg, 72%. ^1^H NMR (400 MHz, CDCl_3_) δ 7.69–7.42 (m, 4H, aromatic), 7.39–7.16 (m, 6H, aromatic), 3.20–2.99 (m, 1H, CH, CHN), 2.51–2.22 (m, 3H, NH_2_ and CHP, overlapped), 1.42 (dt, *J* = 8.8, 7.0 Hz, 2H, CH_2_), 1.06 (d, *J* = 6.0 Hz, 3H, C*H*_3_CHN), 1.04 (dd, *J* = 14.7, 7.0 Hz, 3H, C*H*_3_CHP). ^31^P NMR (162 MHz, CDCl_3_) δ −0.34 (s). ^13^C NMR (101 MHz, CDCl_3_) δ 137.16 (d, *J* = 13.9 Hz, 1C, aromatic), 136.92 (d, *J* = 15.0 Hz, 1C, aromatic), 133.99 (d, *J* = 19.3 Hz, 2C, aromatic), 133.64 (d, *J* = 18.7 Hz, 2C, aromatic), 128.95 (s, 1C, aromatic), 128.85 (s, 1C, aromatic), 128.53 (d, *J* = 6.8 Hz, 2C, aromatic), 128.44 (d, *J* = 7.2 Hz, 2C, aromatic), 45.23 (d, *J* = 12.3 Hz, 1C), 43.58 (d, *J* = 16.9 Hz, 1C), 27.61 (d, *J* = 10.3 Hz, 1C), 22.82 (s, 1C), 16.69 (d, *J* = 14.4 Hz, 1C). HR-MS (ESI) calcd. for C_17_H_23_NP [M + H]^+^: 272.1563; found: 272.1565.

#### 3.1.8. Synthesis of 2-((((2*S*,4*S*)-4-(diphenylphosphino)pentan-2-yl)amino)methyl)phenol (**L12**)

(2*S*,4*S*)-4-(diphenylphosphino)pentan-2-amine (**5**) (300 mg, 1.106 mmol) and freshly distilled salicylaldehyde (135 mg, 1.106 mmol) were dissolved in CH_2_Cl_2_ (3.3 mL) and dry Na_2_SO_4_ (320 mg) was added. The reaction mixture was stirred for 24 h, filtered and the solvent was then removed in vacuo. The residue was dissolved in MeOH (5 mL) and NaBH_4_ (127 mg, 3.317 mmol) was added portionwise to the solution at 20 °C. After being stirred for 1 h at the same temperature, the solvent was removed in vacuo and water (3 mL) was added to the residue. The aqueous phase was extracted with 3 × 3 mL of CH_2_Cl_2_. The combined organic phases were dried with MgSO_4_ and the solvent was removed in vacuo. The crude product was subjected to column chromatography on silica using CHCl_3_/MeOH 16/1 as an eluent to yield 230 mg of the pure product as a white solid. Yield: 55%. ^1^H NMR (400 MHz, Acetone-d_6_) δ 7.65–7.49 (m, 4H, aromatic), 7.45–7.31 (m, 6H, aromatic), 7.10–7.05 (m, 1H, aromatic), 7.01–6.91 (m, 1H, aromatic), 6.71–6.65 (m, 2H, aromatic), 3.92 (ABq, *J* = 14.2 Hz, 2H), 2.89–2.75 (m, 1H, CHN), 2.66–2.52 (m, 1H, CHP), 1.70–1.54 (m, 1H, diast. C*H*H), 1.41 (dtd, *J* = 13.3, 9.3, 3.8 Hz, 1H, diast. CH*H*), 1.11 (d, *J* = 6.3 Hz, 3H, C*H*_3_CHN), 1.00 (dd, *J* = 15.0, 6.8 Hz, 3H, C*H*_3_CHP). ^31^P NMR (162 MHz, Acetone-d_6_) δ 2.00 (s). ^13^C NMR (101 MHz, Acetone-d_6_) δ 159.79 (s, 1C, aromatic), 138.53 (d, *J* = 15.3 Hz, 1C, aromatic), 138.37 (d, *J* = 15.9 Hz, 1C, aromatic), 134.69 (d, *J* = 19.9 Hz, 2C, aromatic), 134.57 (d, *J* = 19.5 Hz, 2C, aromatic), 129.83 (s, 1C, aromatic), 129.79 (s, 1C, aromatic), 129.38 (d, *J* = 7.0 Hz, 2C, aromatic), 129.34 (d, *J* = 7.1 Hz, 2C, aromatic), 129.21 (s, 1C, aromatic), 129.08 (s, 1C, aromatic), 124.50 (s, 1C, aromatic), 119.42 (s, 1C, aromatic), 116.87 (s, 1C, aromatic), 51.29 (d, *J* = 12.2 Hz, 1C), 50.37 (s, 1C), 41.23 (d, *J* = 18.4 Hz, 1C), 27.72 (d, *J* = 9.8 Hz, 1C), 19.70 (s, 1C), 17.09 (d, *J* = 16.5 Hz, 1C). HR-MS (ESI) calcd. for C_24_H_29_NOP [M + H]^+^: 378.1981; found: 378.1975.

### 3.2. Asymmetric Hydrogenation

A mixture of [Ir(COD)Cl]_2_ (5.06 or 2.53 µmol) and the chiral ligand (12.12 or 6.06 µmol) in the corresponding solvent was stirred for 30 min, and then the substrate (5 mmol) was added. A dry glass vial was charged with the base (0.01 or 0.005 µmol) and transferred into a stainless-steel autoclave. The sealed autoclave was carefully purged three times with argon and then hydrogen gas. The solution of the catalyst and the substrate was added to the base via an injection port. After adjusting the final hydrogen pressure, the reaction mixture was stirred for 20 h. After carefully releasing the hydrogen pressure, the crude reaction mixture was purified by column chromatography (silica gel, eluent: *n*-hexane/EtOAc 6/1) to determine the isolated yield of the chiral alcohols. The purified alcohol product was analyzed by HPLC or GC to determine the enantioselectivity of the reaction.

## 4. Conclusions

In conclusion, novel P,N,OH ligands were prepared and applied in the asymmetric hydrogenation of prochiral ketones. The highly modular synthetic approach enabled the thorough screening of the ligand effects. It has been established that in situ-formed iridium complexes modified by pentane-2,4-diyl-based ligands with central chirality are efficient catalysts for the hydrogenation of alkyl-aryl ketones. The presence of the O-H functionality in the ligand is the prerequisite for the high enantioselectivity. It was found that ligands with an O-methyl substituent coordinate in a bidentate fashion that might be responsible for reduced enantioselectivity. Additionally, the mechanistic role of the O-H functionality has been rationalized by comparative experiments. It is reasonable to assume that the hydrogenation preferentially occurs via a hydroxyl-promoted pathway, instead of the involvement of the NH site. The pentane-2,4-diyl-based ligand **L1** provided high enantioselectivities (up to 98% *ee*) and activities in the asymmetric hydrogenation of alkyl-aryl ketones and could be used at extremely high substrate concentrations without a significant decrease in the enantioselectivity. We believe that the findings reported in the present study can be extrapolated to other simple alkane-diyl-based chiral ligands and lead to chiral transition metal complexes with improved catalytic properties.

## Figures and Tables

**Table 1:** Optimization of reaction conditions using **L1**.

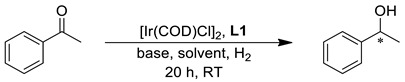					
Entry	Base	B/C Molar Ratio	Solvent	Conversion (%)	*ee* (%) ^a^
1	*t*BuOLi	5	*i*PrOH	76	88
2	*t*BuOK	5	*i*PrOH	>99	82
3	*t*BuOLi	10	*i*PrOH	>99	92
4	*t*BuOLi	20	*i*PrOH	>99	92
5	KOH	10	*i*PrOH	>99	80
6	*t*BuOLi	10	MeOH	98	72
7	*t*BuOLi	10	EtOH	>99	94
8	*t*BuOLi	10	*n*BuOH	>99	95
9	*t*BuOLi	10	*γ*-valerolactone	3	60
10	*t*BuOLi	10	2-Me-THF	9	39
11	*t*BuOLi	10	96% EtOH	>99	93
12 ^b^	*t*BuOLi	10	EtOH	>99	95

Reaction conditions: Acetophenone: 5 mmol, [Ir(COD)Cl]_2_: 0.1 mol%, **L1**: 0.24 mol%, solvent: 0.5 mL, temperature: RT, reaction time: 20 h, H_2_ pressure: 30 bar. ^a^ The prevailing product enantiomer is (*R*) in each case. The conversion and enantioselectivity were determined by GC equipped with a chiral column. ^b^ [Ir(COD)Cl]_2_: 0.05 mol%, **L1**: 0.12 mol%.

**Table 2:** Asymmetric hydrogenation of acetophenone: the effect of substrate concentration.

Substrate Concentration (mmol Acetophenone/mL Ethanol)	Conversion (%)	*ee* (%)
5	>99	94
10	>99	94
20	>99	93
50	>99	92
100	>99	90

Reaction conditions: Acetophenone: 5 mmol, [Ir(COD)Cl]_2_: 0.1 mol%, **L1**: 0.24 mol%, solvent: EtOH, base: 2 mol% of *t*BuOLi. Reaction time: 20 h, temperature: RT, H_2_ pressure: 30 bar. The conversion and enantioselectivity were determined by GC equipped with a chiral column. The prevailing product enantiomer is (*R*) in each case.

## Data Availability

The data presented in this study are available in the article or in the Supplementary File.

## Supplementary Materials

The following supporting information can be downloaded at: https://www.mdpi.com/article/10.3390/molecules29163743/s1, Figure S1–S24: ^1^H, ^31^P{^1^H} and ^13^C{^1^H} NMR spectra of the novel compounds **L4**, **L5**, **L7**, **L8-10**, **5** and **L12**. Analytical data (^1^H NMR, conditions of the chromatographic separation, GC and HPLC retention times) of the hydrogenation products **P1**-**22** [12,22,43,47,48,49,50,51,52,53,54]. Figure S25–S68: Chromatograms (chiral GC or HPLC) of **P1**-**P22** and their racemic forms. Details of the DFT calculations [55,56,57,58,59,60,61,62]. Table S1: relative enthalpies of the optimized structures **A–H**.
