# Supplementary material for: Asymmetric Hydrogenation of Ketones by Simple Alkane-Diyl-Based Ir(P,N,O) Catalysts: A Comparative Study
Source: Molecules. 2024 Aug 7;29(16):3743. doi: 10.3390/molecules29163743 (PMC11357652; doi:10.3390/molecules29163743)
Supplement: Supplementary file 1 [file molecules-29-03743-s001.zip › molecules-3137771-supplementary.pdf]

Asymmetric Hydrogenation of Ketones by Simple Alkane-Diyl-Based Ir(P,N,O) Catalysts: A Comparative Study

Zsófia Császár [1], Mária Guóth [1], Margit Kovács [2], Attila C. Bényei [3], József Bakos [1,*] and Gergely Farkas [1,*]

[1] Research Group of Organic Chemistry—Synthesis and Catalysis, University of Pannonia, Egyetem u. 10, H-8200 Veszprém, Hungary; csaszar.zsofia@mk.uni-pannon.hu (Z.C.); guoth.marika@gmail.com (M.G.)
[2] NMR Laboratory, University of Pannonia, Egyetem u. 10, H-8200 Veszprém, Hungary; kovacs.margit@mk.uni-pannon.hu
[3] Department of Physical Chemistry, University of Debrecen, Egyetem tér 1, H-4032 Debrecen, Hungary; benyei.attila@science.unideb.hu
* Correspondence: bakos.jozsef@mk.uni-pannon.hu (J.B.); farkas.gergely@mk.uni-pannon.hu (G.F.)

Table of contents

1. NMR spectra of the new compounds 2
2. Characterization of the hydrogenation products 14
3. GC and HPLC chromatograms 20
4. DFT computational studies 42

1. NMR spectra of the new compounds

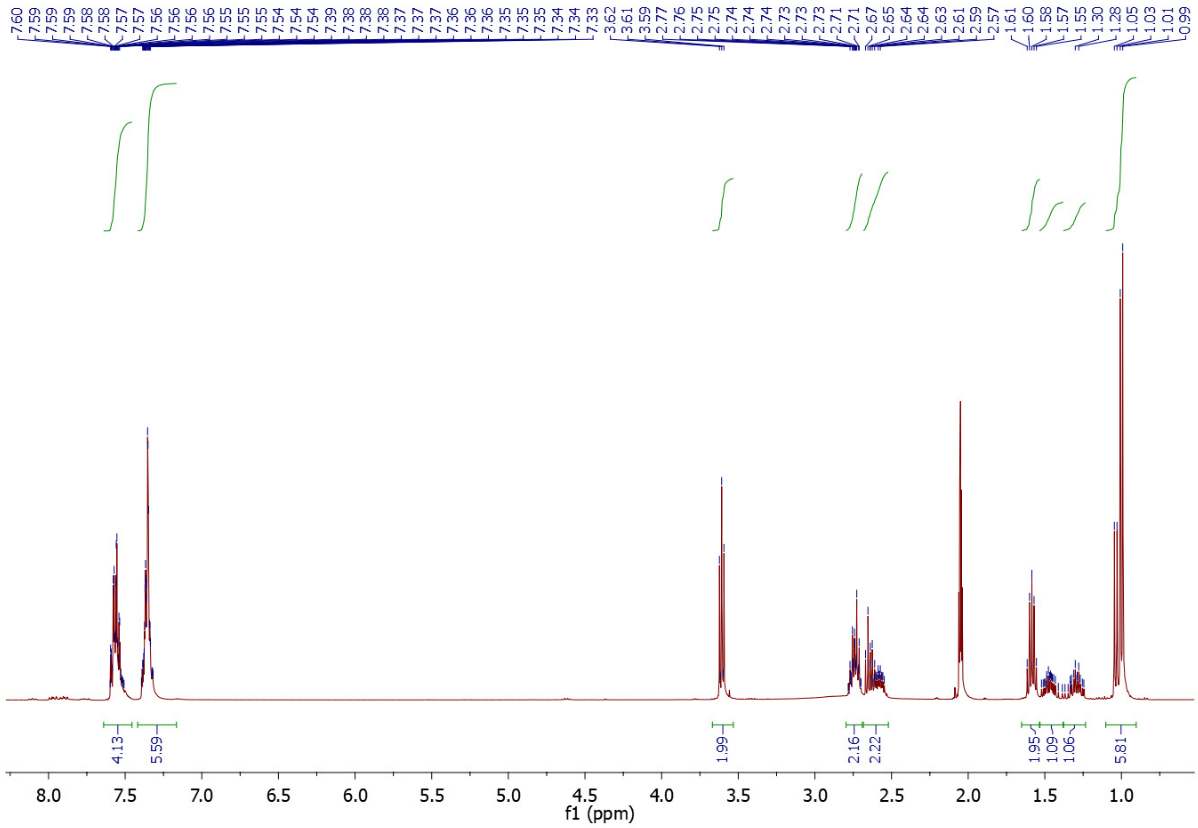

Figure S1. ^{1}H NMR spectrum of ligand **L4**

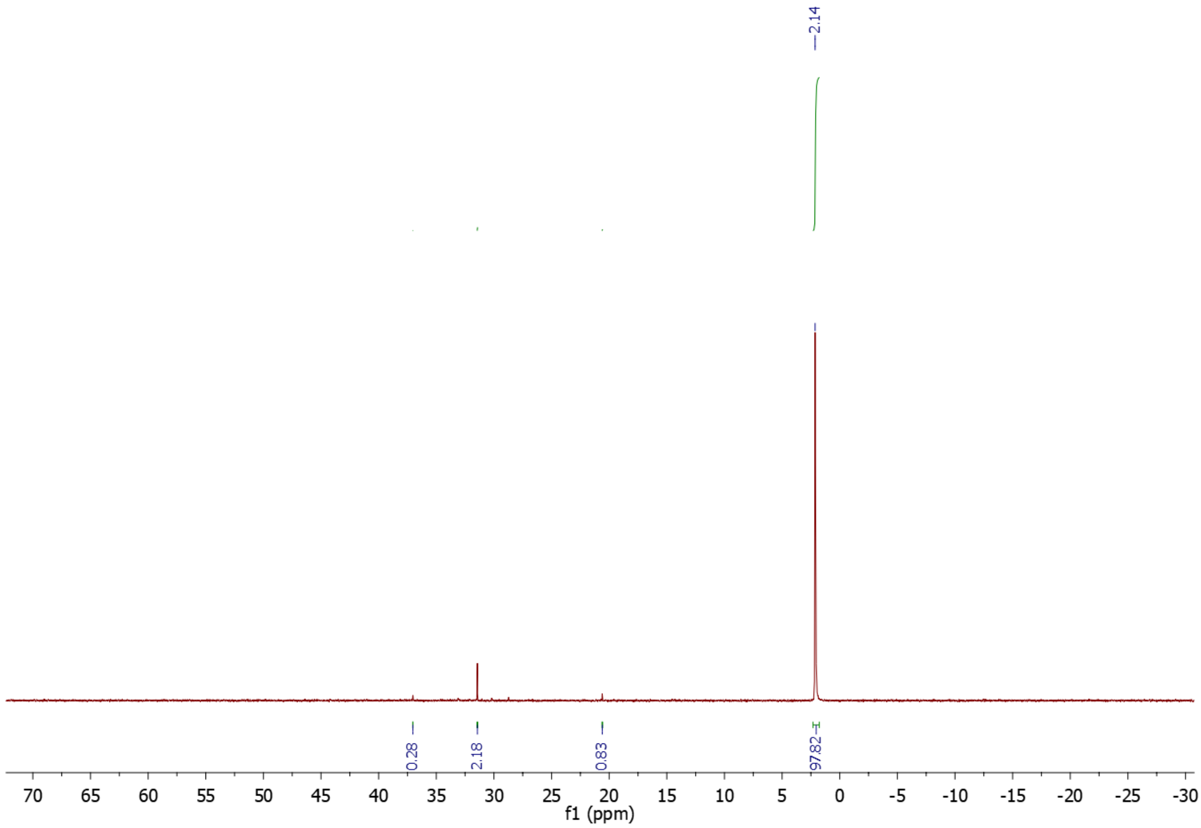

Figure S2. $^{31}P\{^{1}H\}$ NMR spectrum of ligand **L4**

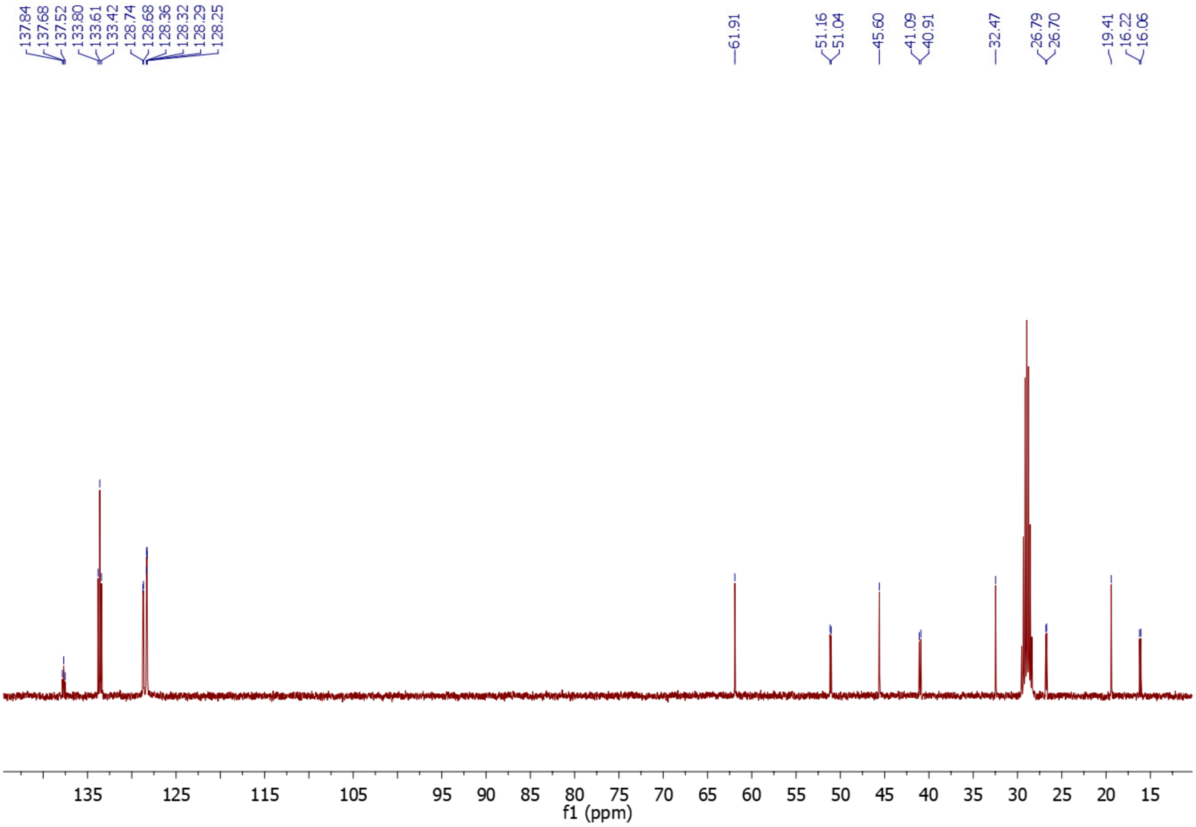

Figure S3. $^{13}C\{^{1}H\}$ NMR spectrum of ligand **L4**

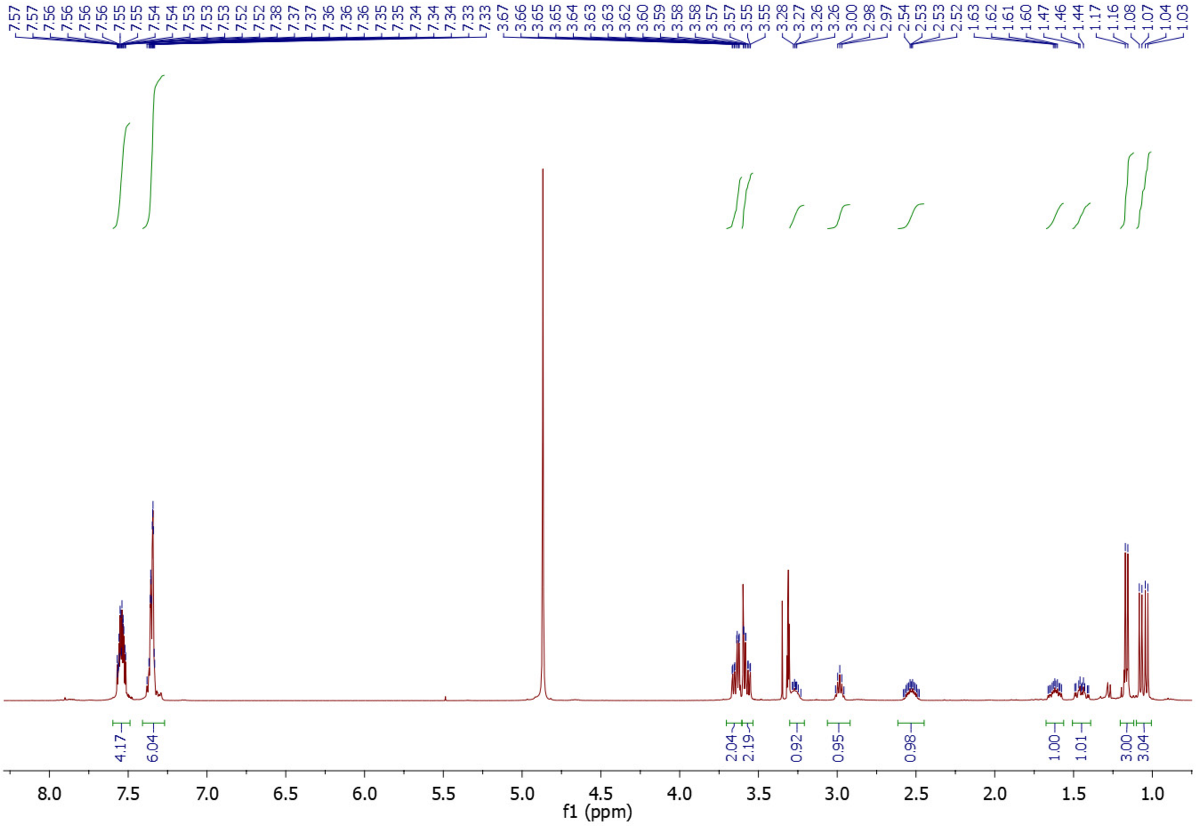

Figure S4. ^{1}H NMR spectrum of ligand **L5**

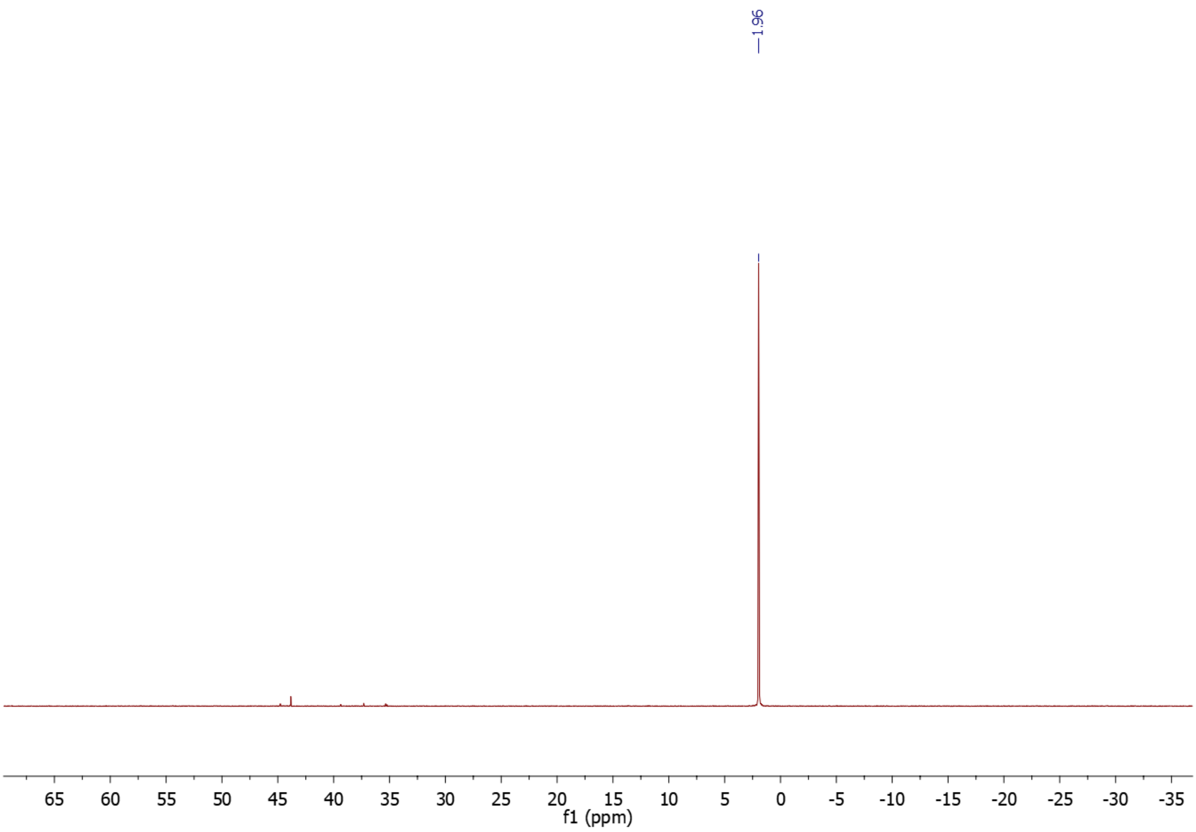

Figure S5. $^{31}P\{^{1}H\}$ NMR spectrum of ligand **L5**

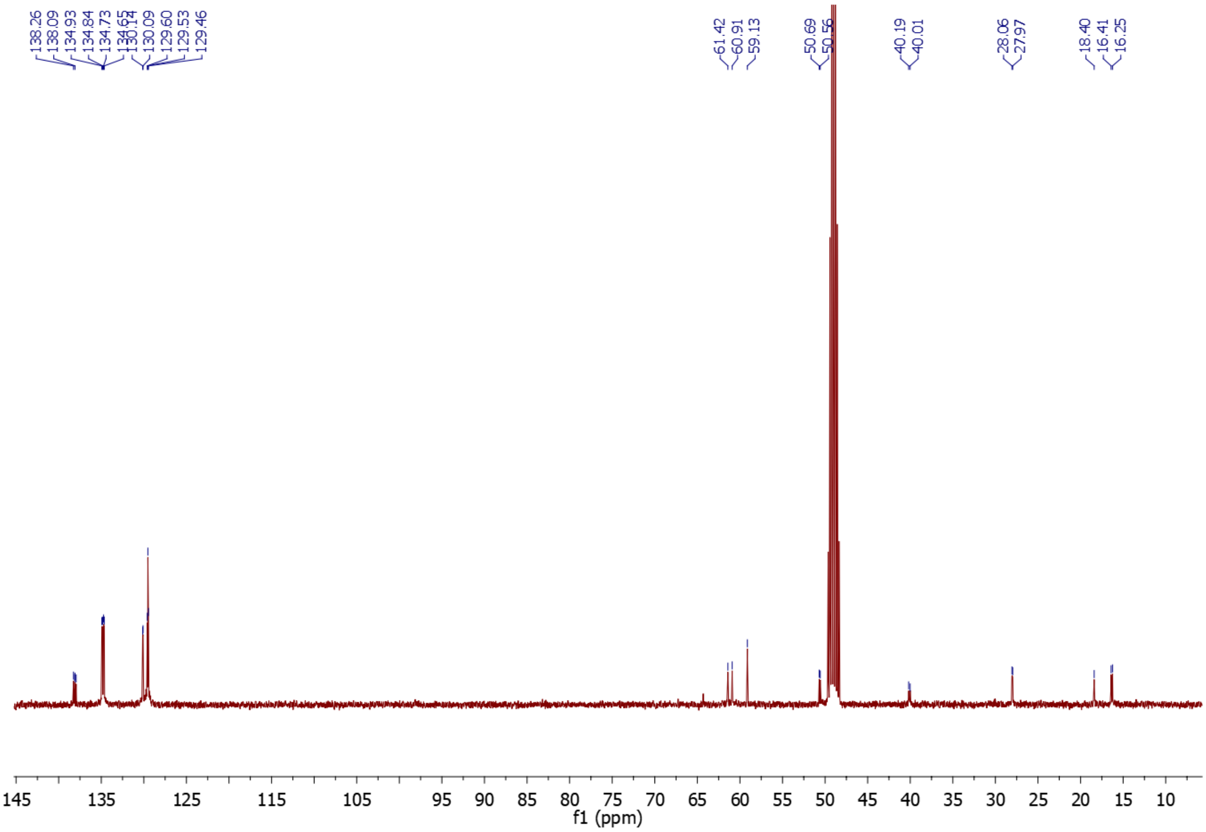

Figure S6. $^{13}C\{^{1}H\}$ NMR spectrum of ligand **L5**

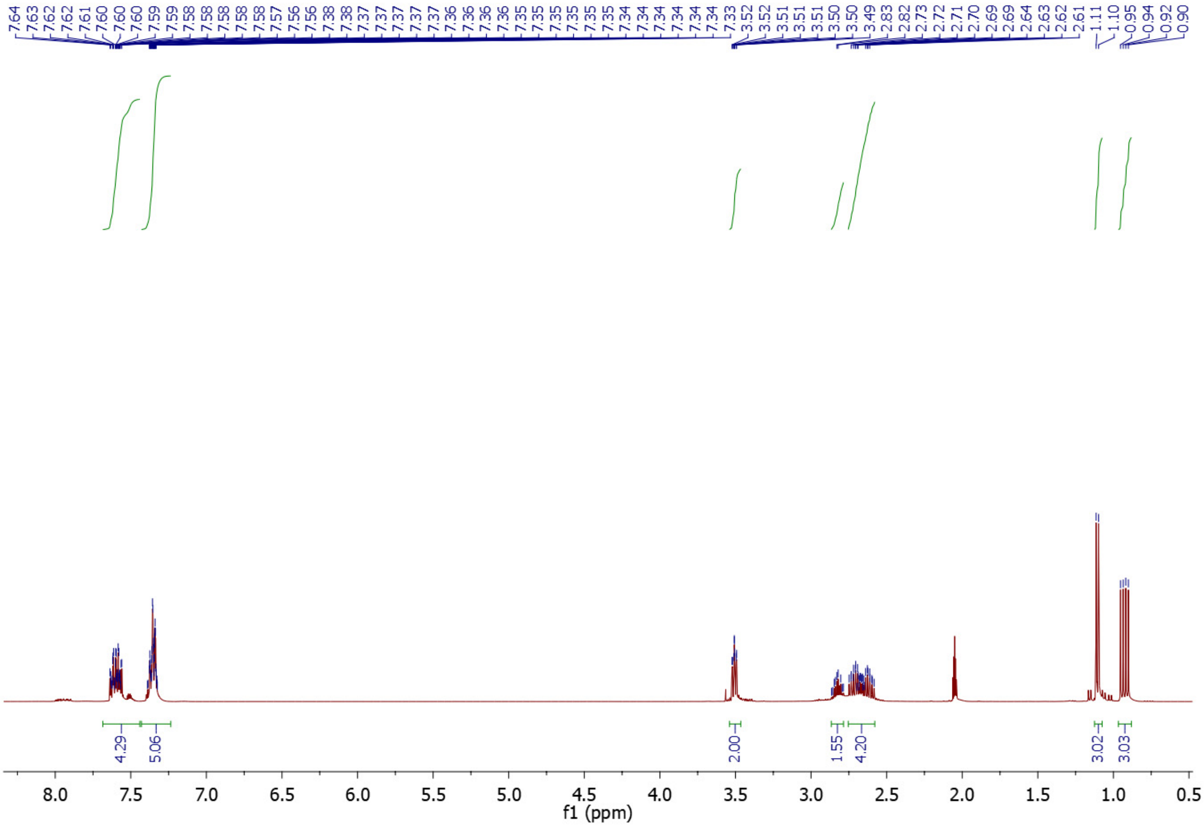

Figure S7. ^{1}H NMR spectrum of ligand **L7**

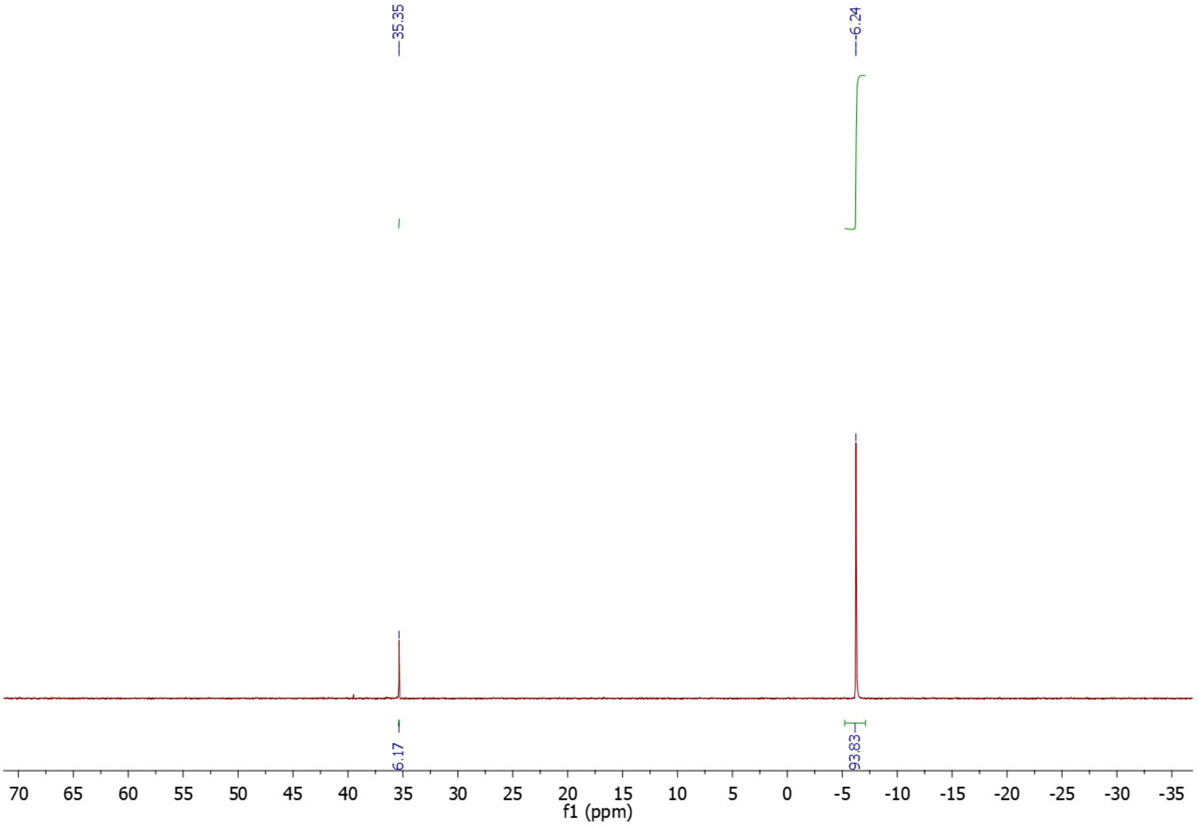

Figure S8. $^{31}P\{^{1}H\}$ NMR spectrum of ligand **L7**

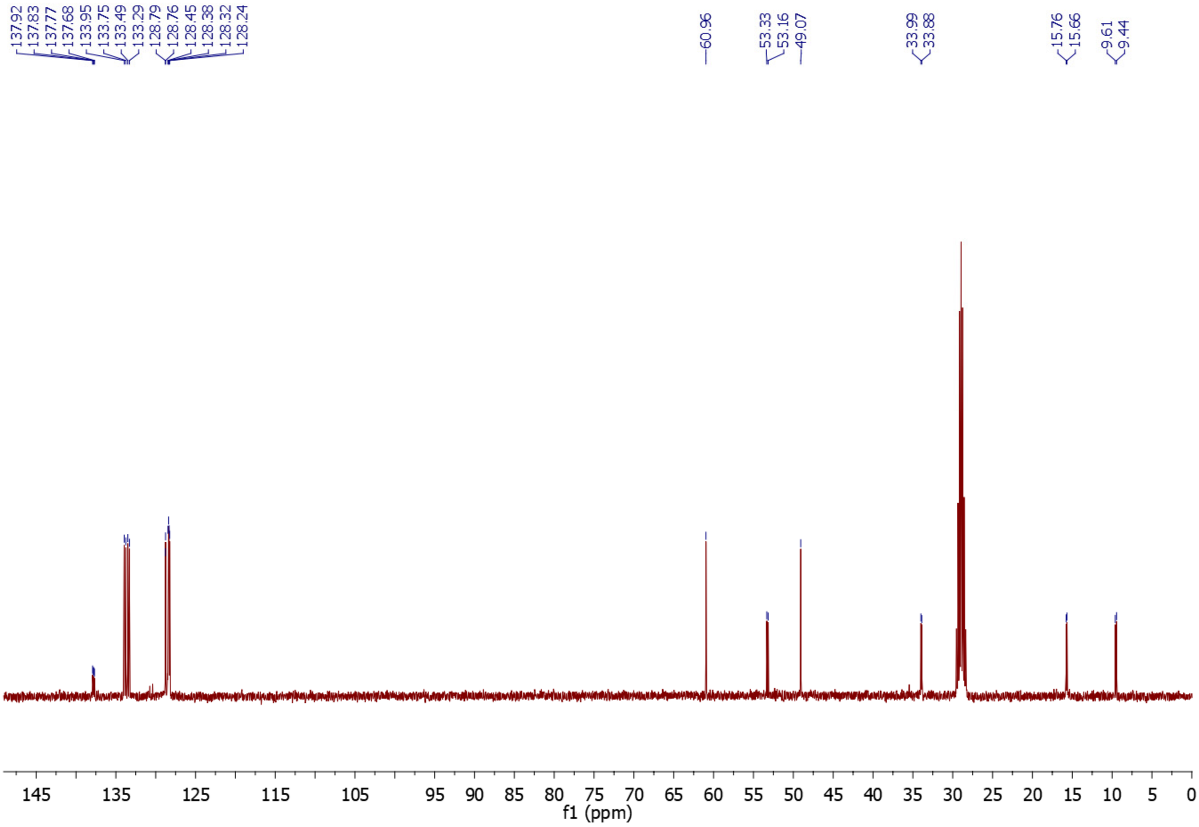

Figure S9. $^{13}C\{^{1}H\}$ NMR spectrum of ligand **L7**

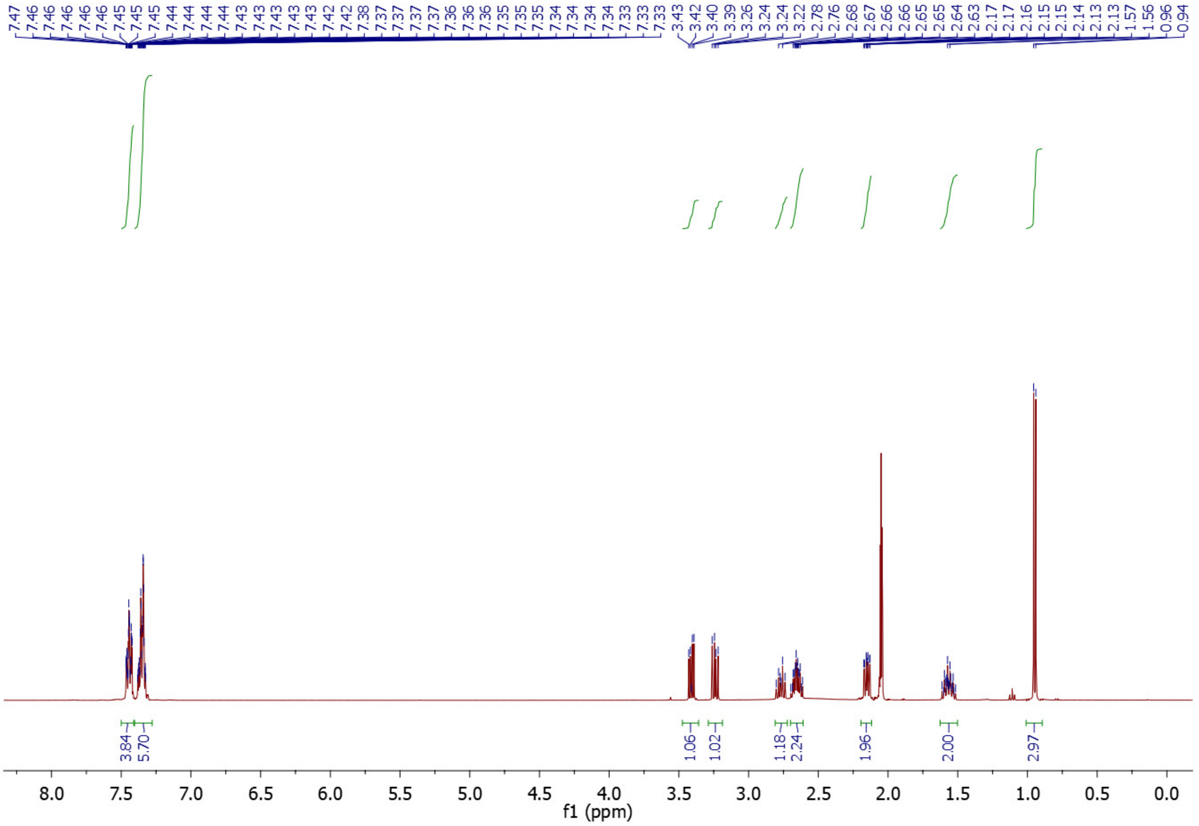

Figure S10. ^{1}H NMR spectrum of ligand **L8**

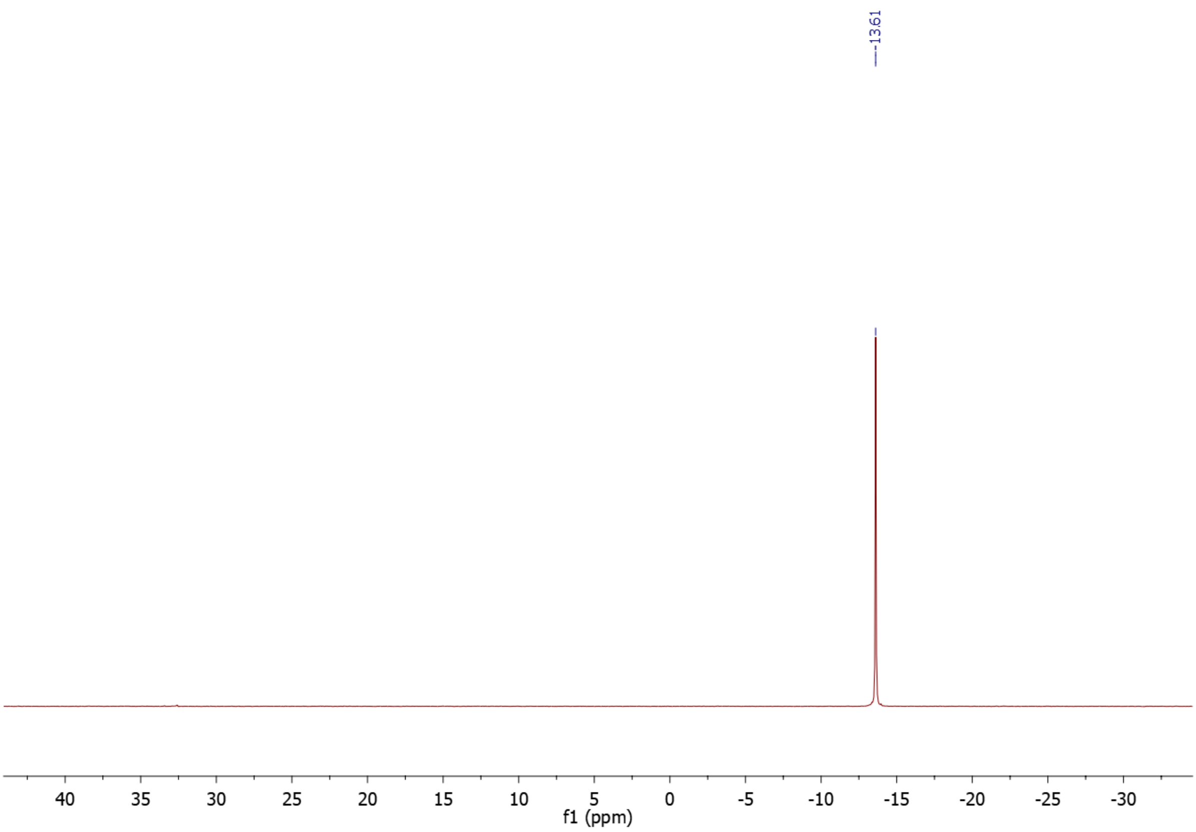

Figure S11. $^{31}P\{^{1}H\}$ NMR spectrum of ligand **L8**

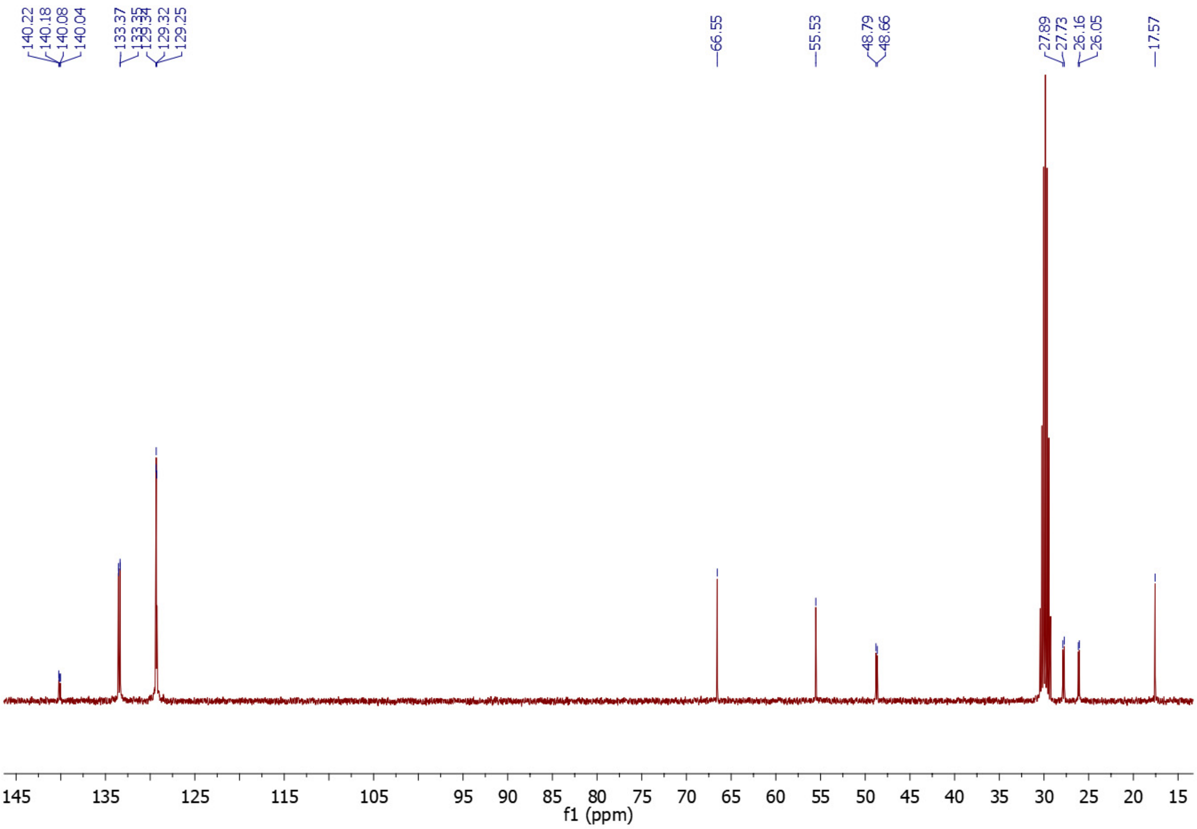

Figure S12. $^{13}C\{^{1}H\}$ NMR spectrum of ligand **L8**

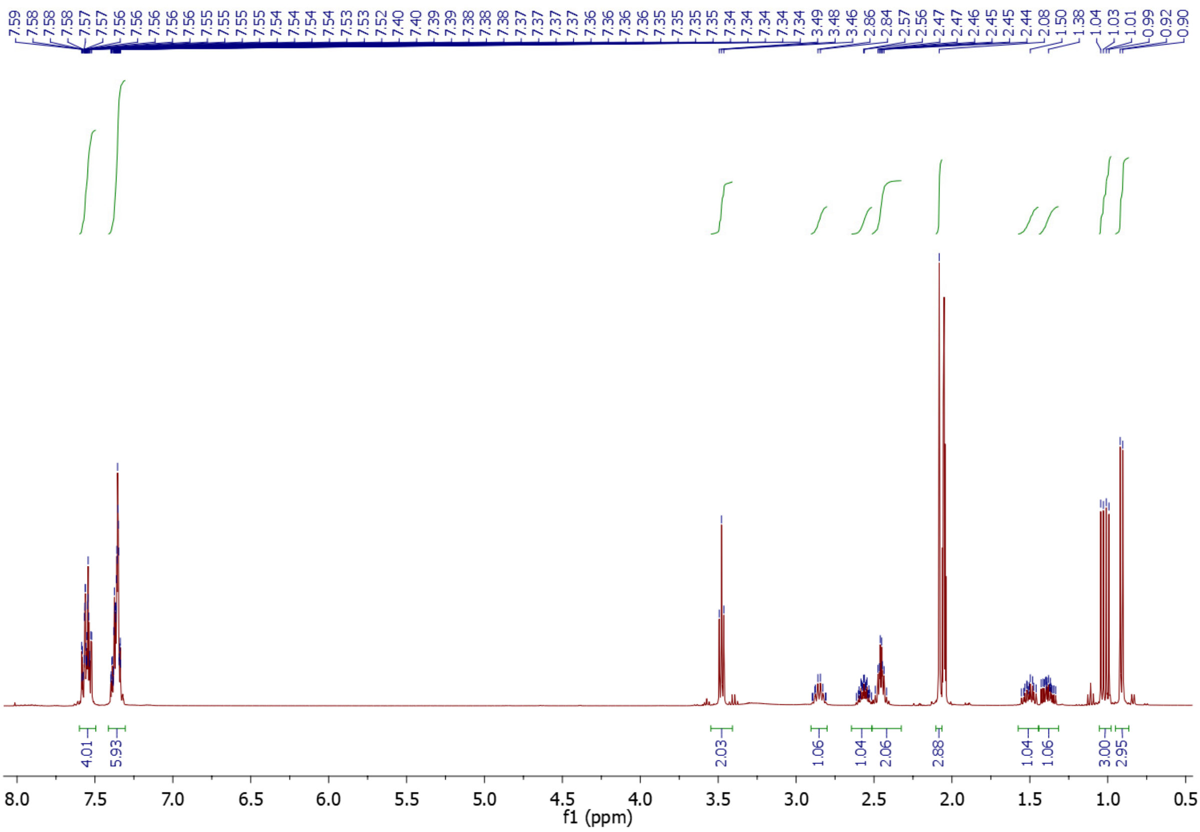

Figure S13. ^{1}H NMR spectrum of ligand **L9**

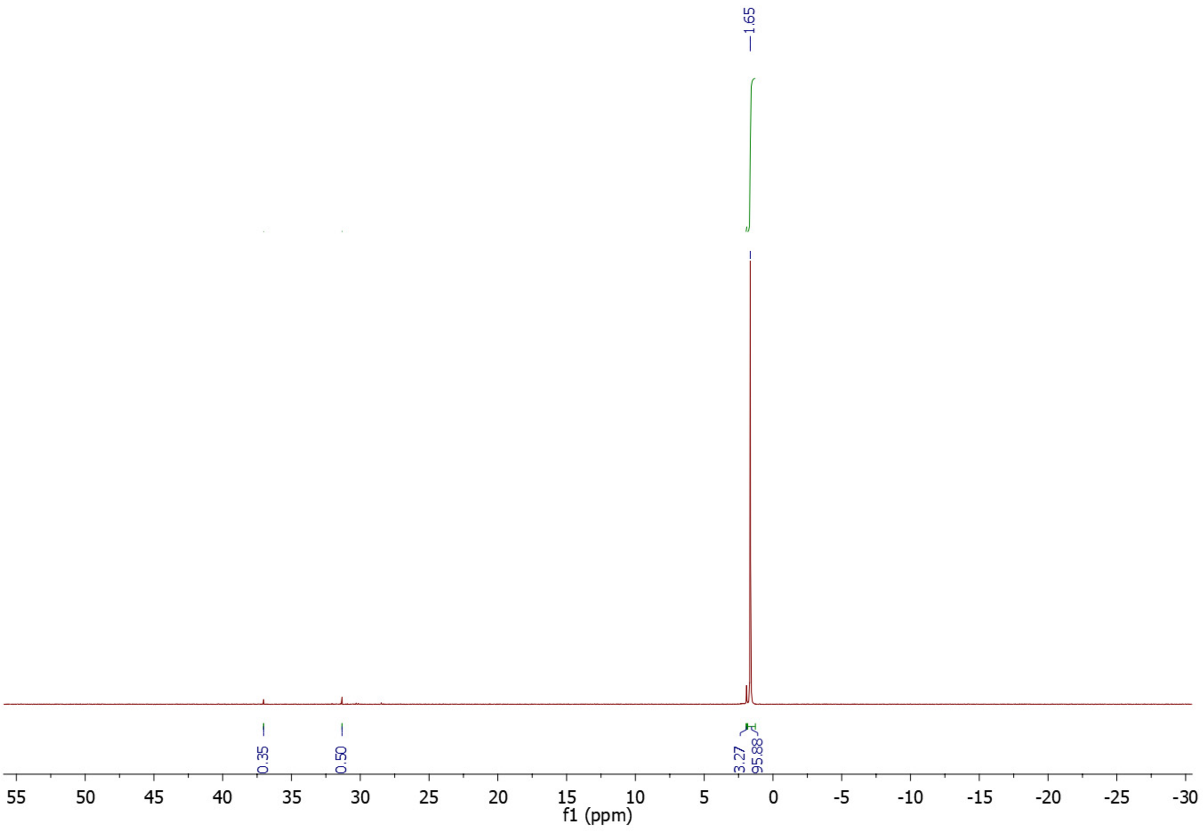

Figure S14. $^{31}P\{^{1}H\}$ NMR spectrum of ligand **L9**

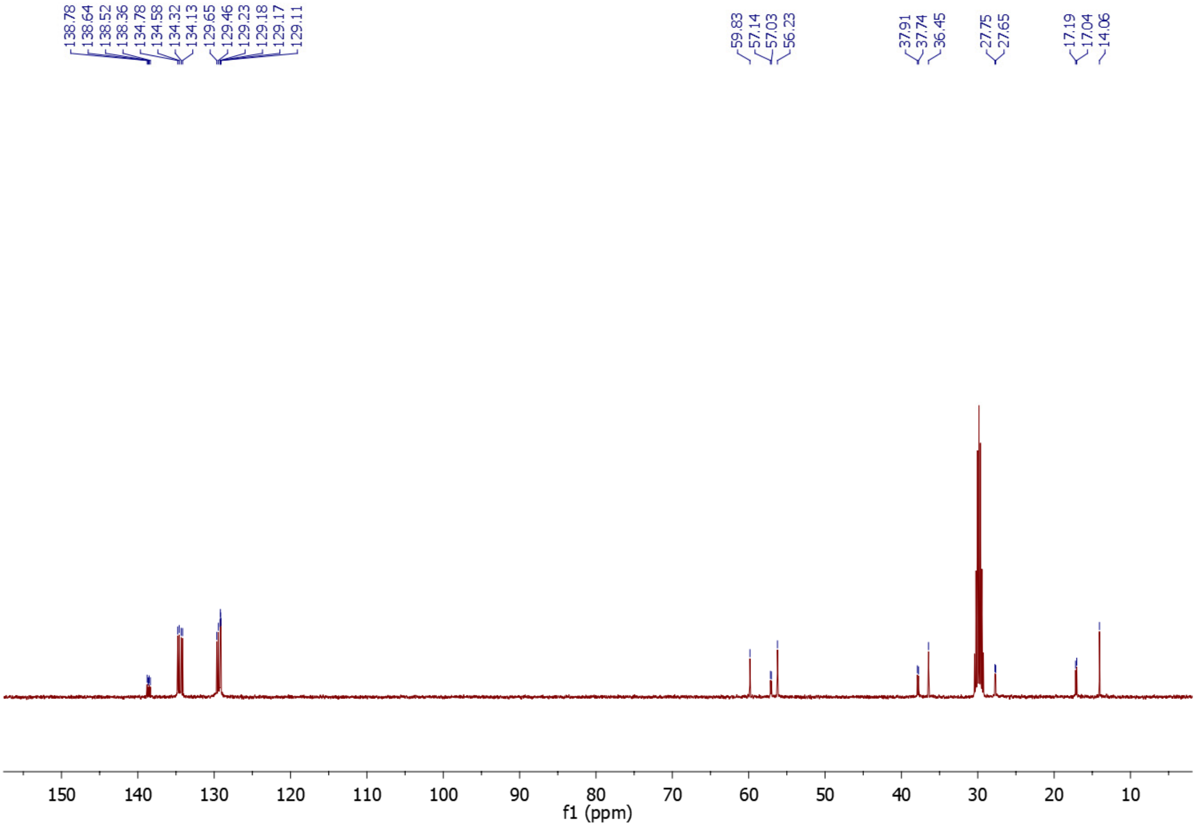

Figure S15. $^{13}C\{^{1}H\}$ NMR spectrum of ligand **L9**

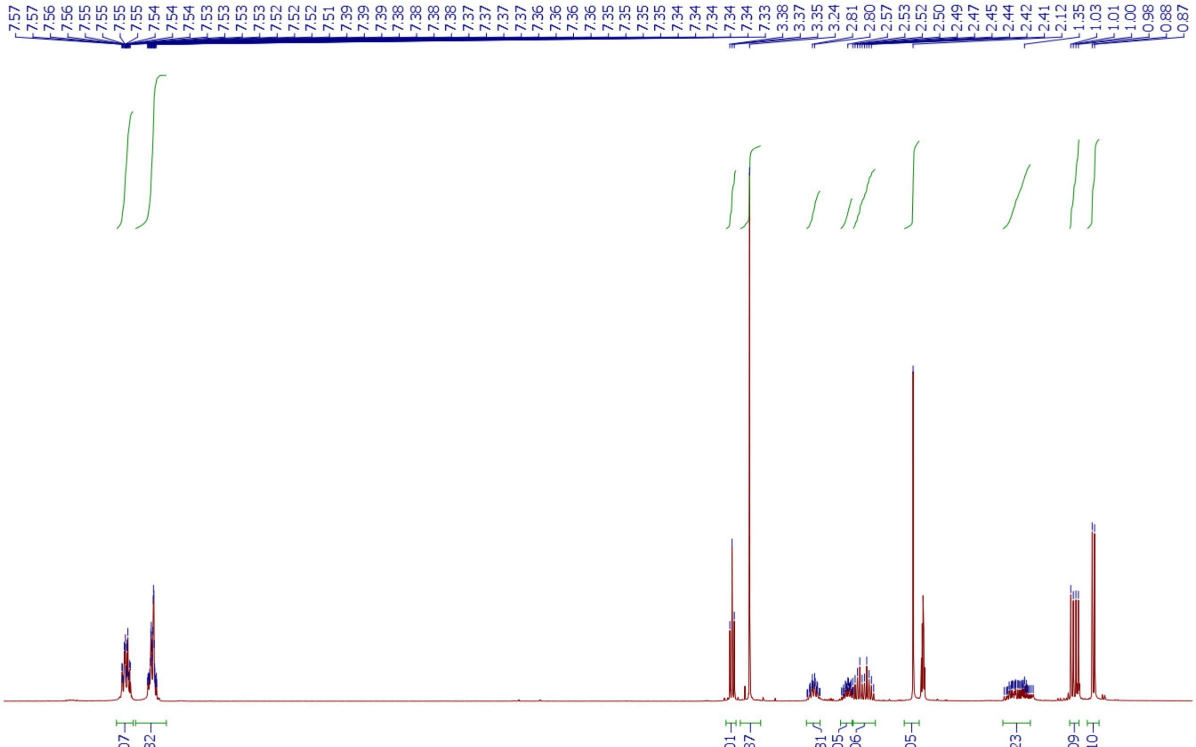

Figure S16. ^{1}H NMR spectrum of ligand **L10**

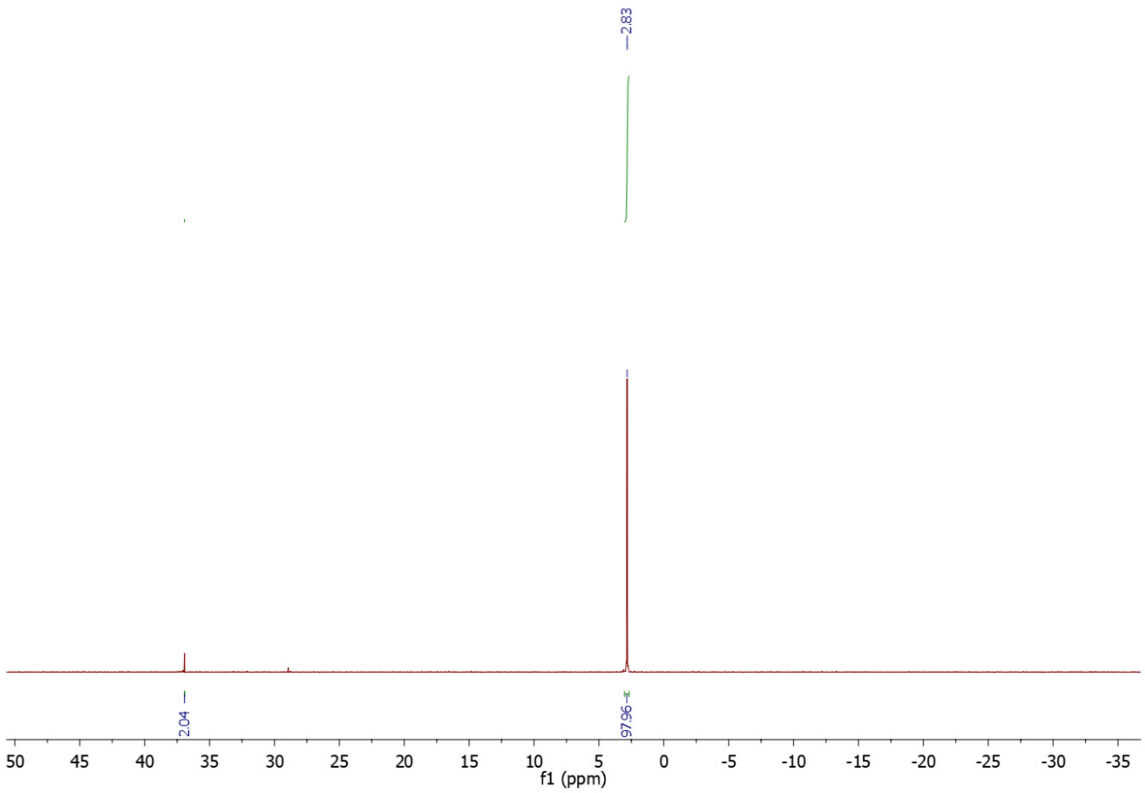

Figure S17. $^{31}P\{^{1}H\}$ NMR spectrum of ligand **L10**

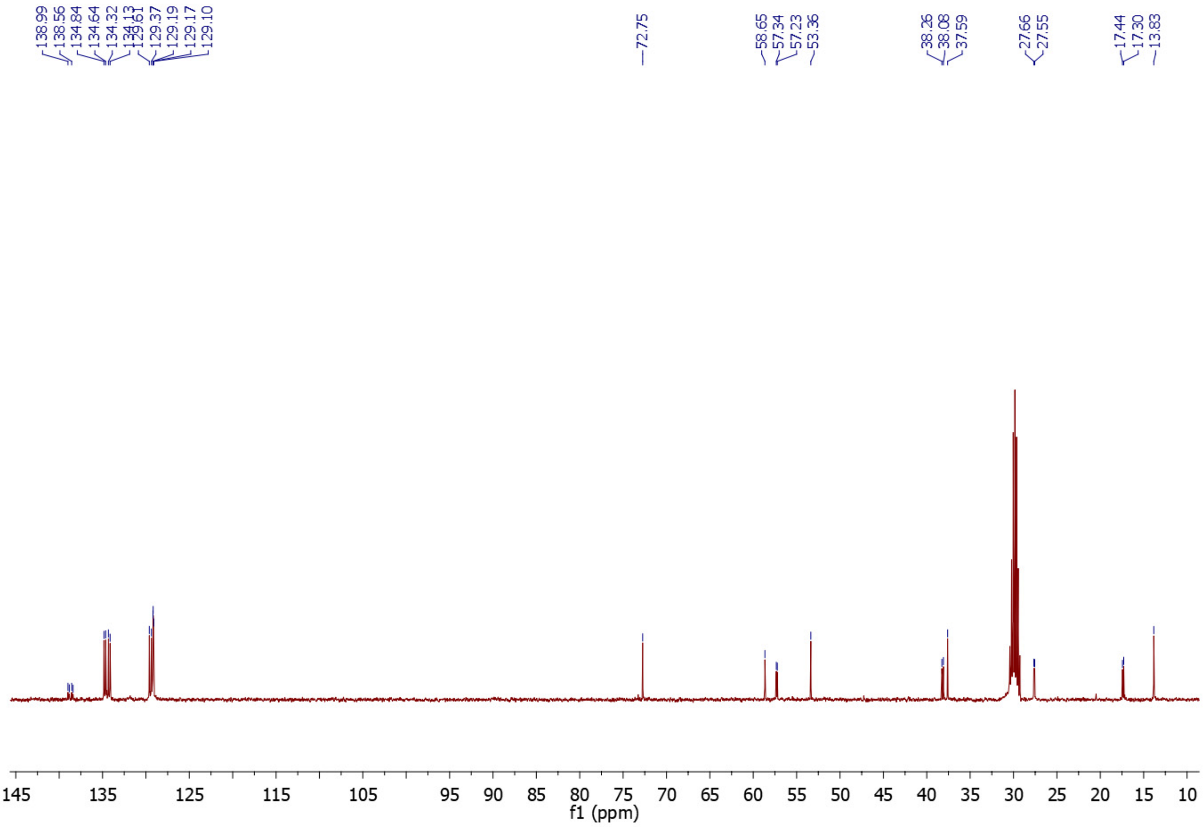

Figure S18. $^{13}C\{^{1}H\}$ NMR spectrum of ligand **L10**

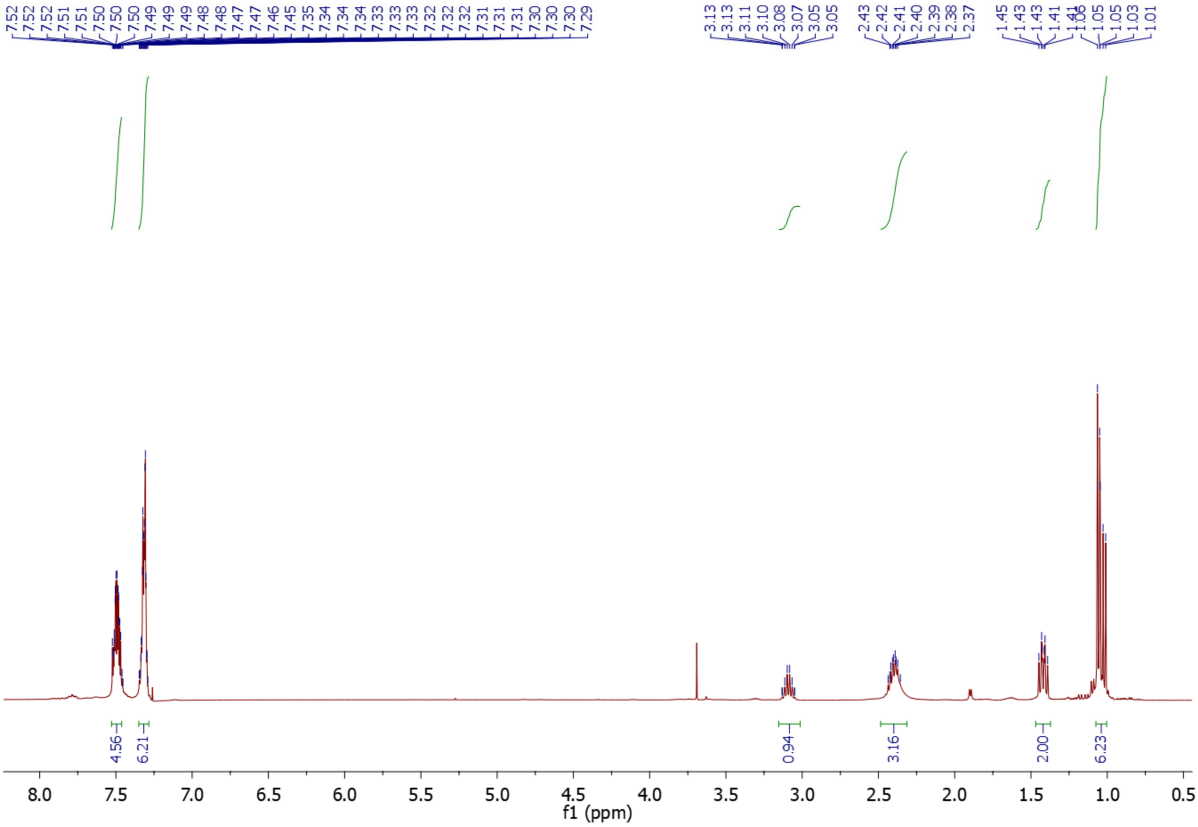

Figure S19. ^{1}H NMR spectrum of compound **5**

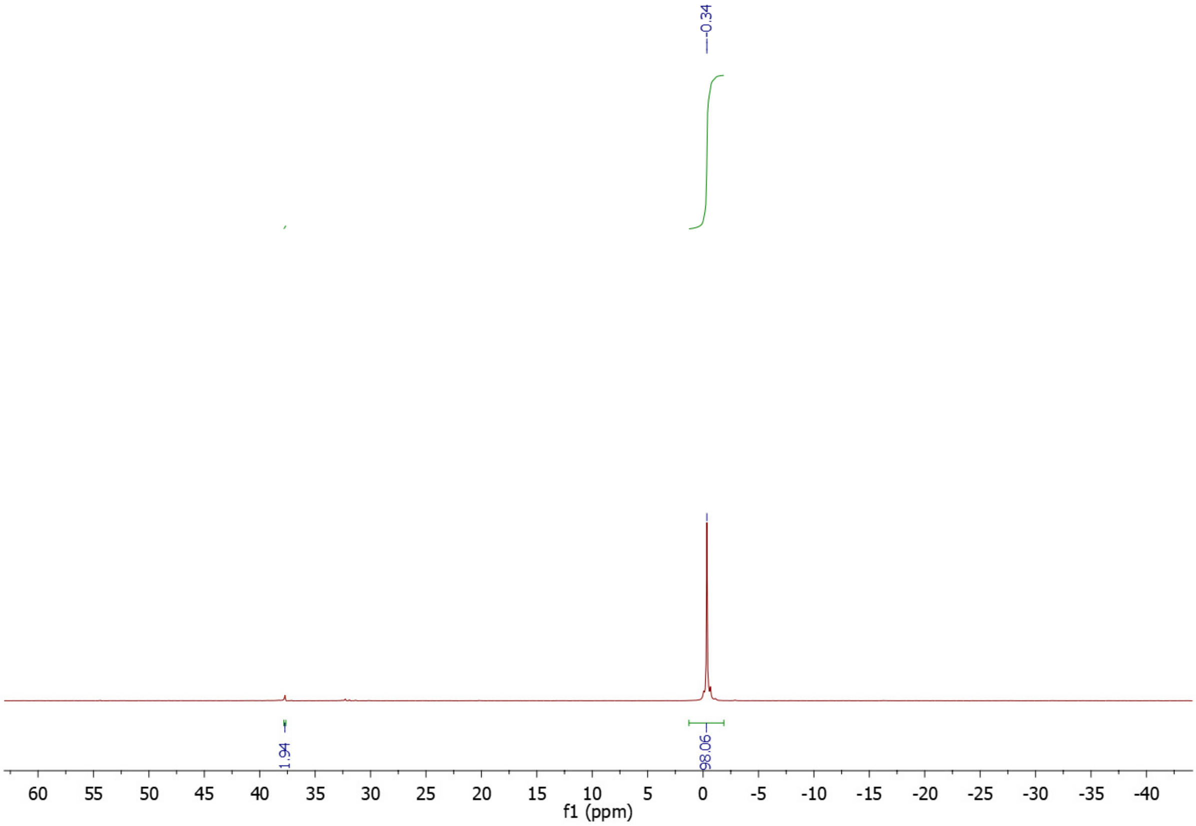

Figure S20. $^{31}P\{^{1}H\}$ NMR spectrum of compound **5**

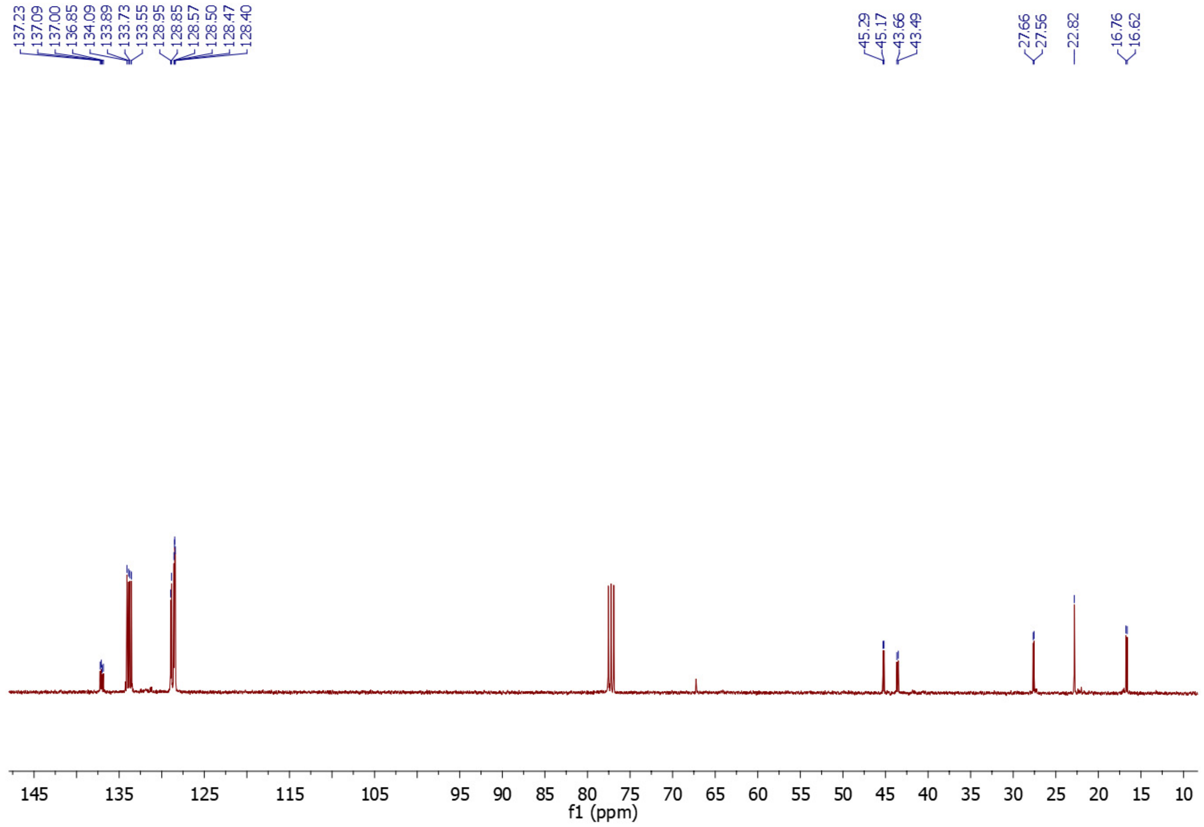

Figure S21. $^{13}C\{^{1}H\}$ NMR spectrum of compound **5**

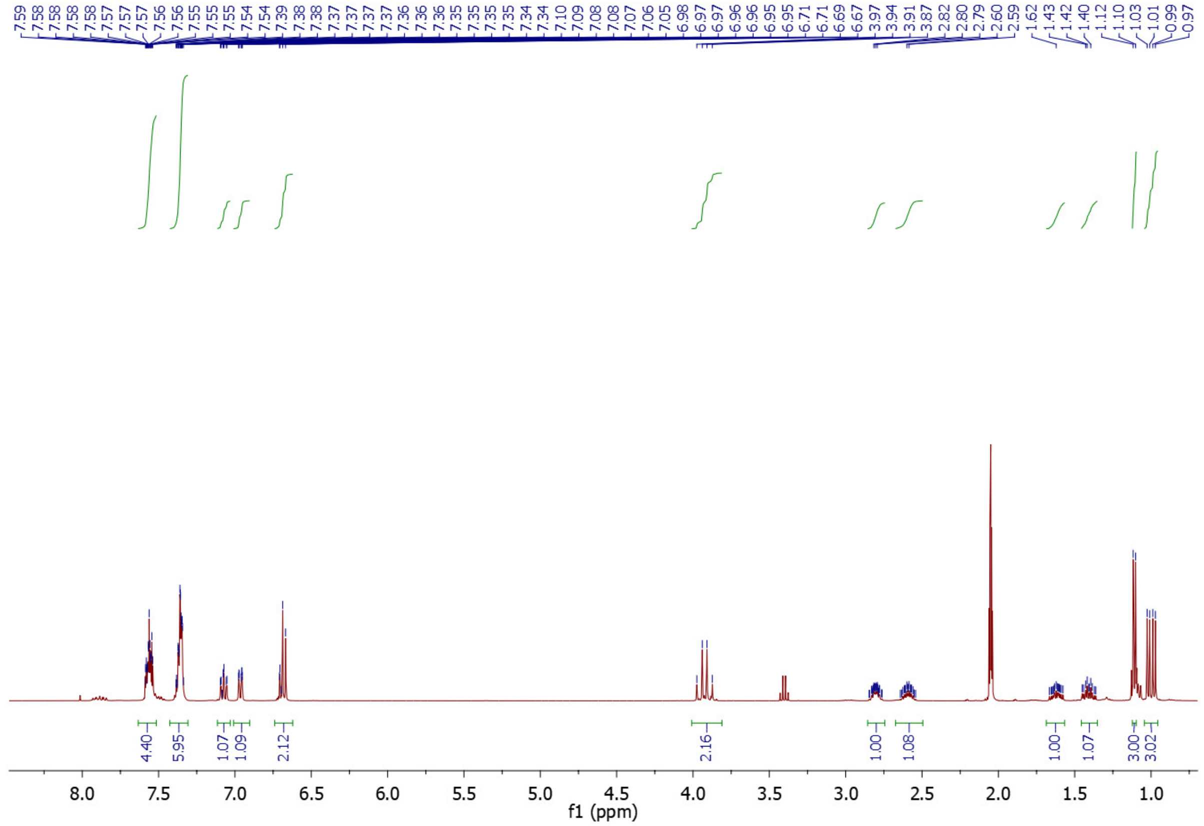

Figure S22. ^{1}H NMR spectrum of ligand **L12**

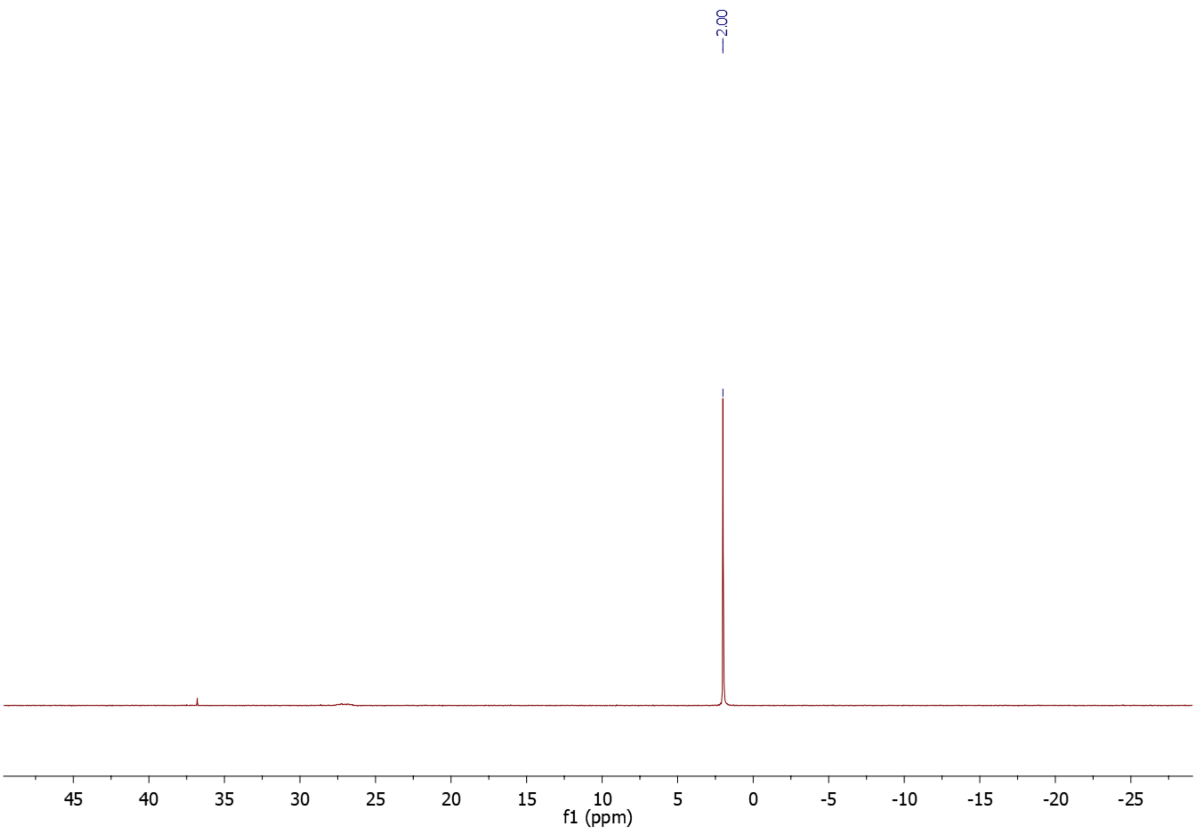

Figure S23. $^{31}P\{^{1}H\}$ NMR spectrum of ligand **L12**

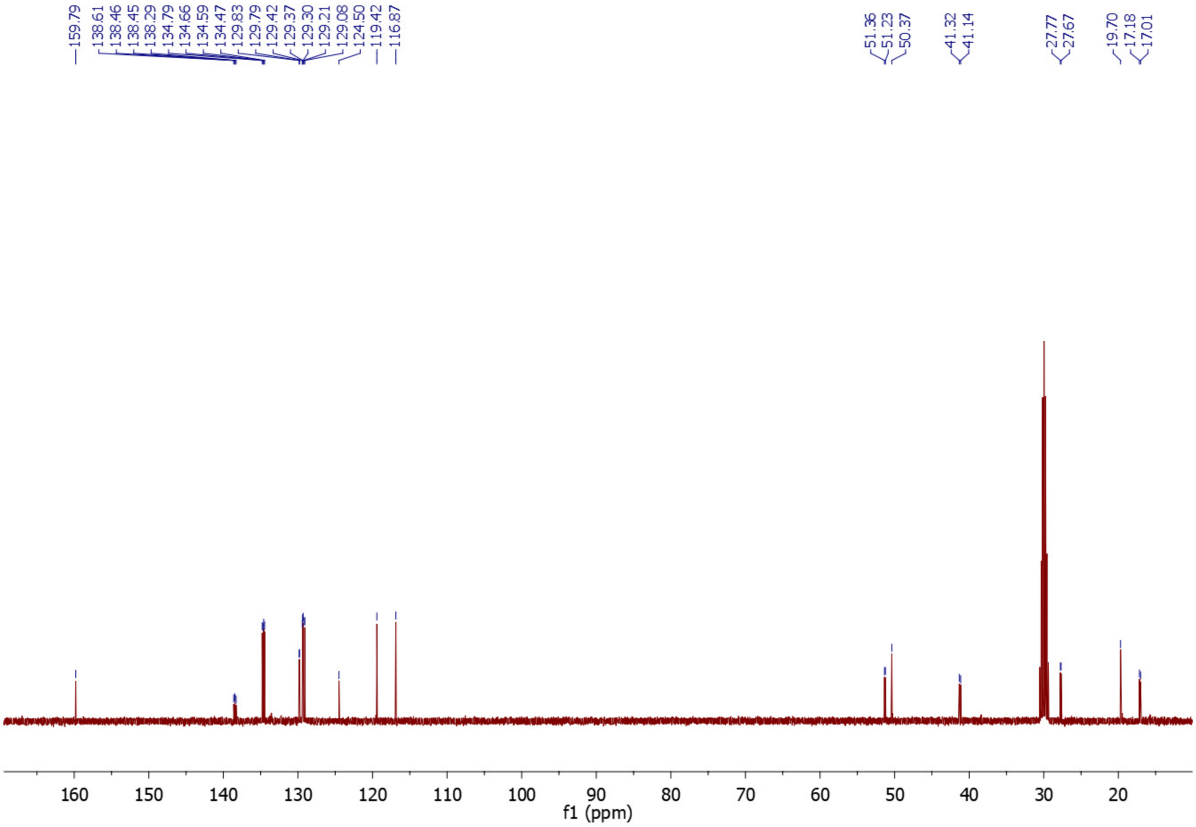

Figure S24. $^{13}C\{^{1}H\}$ NMR spectrum of ligand **L12**

2. Characterization of the hydrogenation products

(*R*)-1-phenylethanol (**P1**)

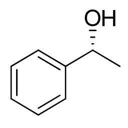

95% *ee* (*R*). ^{1}H NMR (400 MHz, $CDCl_3$) δ 7.38 – 7.30 (m, 4H, aromatic), 7.29 – 7.22 (m, 1H, aromatic), 4.88 (q, *J* = 6.5 Hz, 1H, CH), 1.85 (br. m, 1H, OH), 1.48 (d, *J* = 6.5 Hz, 3H, CH_3) ppm. GC separation conditions: Supelco β-DEX™ 225, L x I.D. 30 m x 0.25 mm, df 0.25 µm, carrier gas: N_2 (gas flow rate: 2 mL/min), injection temperature: 250°C, detector temperature: 250°C. Temperature profile: 80°C for 10 min, from 80°C to 120°C with ramp of 5°C/min, 120°C for 10 min, from 120°C to 180°C with ramp of 10°C/min, 180°C for 15 min. Retention times: t_R(acetophenone) = 15.1 min, t_R(*R*) = 17.9 min, t_R(*S*) = 18.4 min.[1]

(*R*)-1-(p-tolyl)ethanol (**P2**)

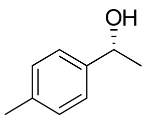

95% *ee* (*R*). ^{1}H NMR (400 MHz, $CDCl_3$) δ 7.32 – 7.25 (m, 2H, aromatic), 7.22 – 7.16 (m, 2H, aromatic), 4.89 (q, *J* = 6.4 Hz, 1H, CH), 2.38 (s, 3H, CH_3), 1.87 (br. s, 1H, OH), 1.51 (d, *J* = 6.5 Hz, 3H, CH_3) ppm. GC separation conditions: Supelco β-DEX™ 225, L x I.D. 30 m x 0.25 mm, df 0.25 µm, carrier gas: N_2 (gas flow rate: 2 mL/min), injection temperature: 250°C, detector temperature: 250°C. Temperature profile: from 100°C to 160°C with ramp of 1°C/min, from 160°C to 200°C with ramp of 10°C/min. Retention times: t_R(*R*) = 22.1 min, t_R(*S*) = 23.1 min.[2]

(*R*)-1-(4-isobutylphenyl)ethanol (**P3**)

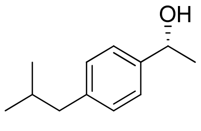

93% *ee* (*R*). ^{1}H NMR (400 MHz, $CDCl_3$) δ 7.33 – 7.29 (m, 2H, aromatic), 7.19 – 7.13 (m, 2H, aromatic), 4.89 (q, *J* = 6.4 Hz, 1H, CH), 2.50 (d, *J* = 7.2 Hz, 2H, CH_2), 1.98 (br. s, 1H, OH), 1.96 – 1.82 (m, 1H, CH), 1.51 (d, *J* = 6.5 Hz, 3H, CH_3), 0.94 (d, *J* = 6.6 Hz, 6H, CH_3). HPLC conditions: Kromasil 3-CelluCoat column, eluent: n-hexane/*i*PrOH 95/5, λ = 220 nm, flow rate: 0.5 mL/min. Retention times: t_R(*R*) = 6.8 min, t_R(*S*) = 7.8 min.[2]

(*R*)-1-(4-methoxyphenyl)ethanol (**P4**)

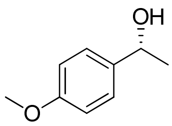

90% *ee* (*R*). ^{1}H NMR (400 MHz, $CDCl_3$) δ 7.35 – 7.30 (m, 2H, aromatic), 6.93 – 6.88 (m, 2H, aromatic), 4.88 (q, *J* = 6.4 Hz, 1H, CH), 3.83 (s, 3H, OCH_3), 1.80 (br. s, 1H, OH), 1.50 (d, *J* = 6.4 Hz, 3H, CH_3) ppm. HPLC conditions: Kromasil

3-CelluCoat column, eluent: n-hexane/*i*PrOH 95/5, λ = 220 nm, flow rate: 0.5 mL/min. Retention times: $t_R(R)$ = 12.1 min, $t_R(S)$ = 13.4 min.[3]

(*R*)-1-(4-fluorophenyl)ethanol (**P5**)

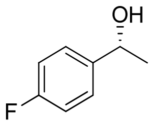

87% *ee* (*R*). ^{1}H NMR (400 MHz, $CDCl_3$) δ 7.37 – 7.29 (m, 2H, aromatic), 7.06 – 6.99 (m, 2H, aromatic), 4.88 (q, *J* = 6.4 Hz, 1H, CH), 1.95 (br. s, 1H, OH), 1.47 (d, *J* = 6.5 Hz, 3H, CH_3) ppm. GC conditions: Supelco β-DEX™ 225, L x I.D. 30 m x 0.25 mm, df 0.25 μm, carrier gas: N_2 (gas flow rate: 2 mL/min), injection temperature: 250°C, detector temperature: 250°C. Temperature profile: from 100°C to 160°C with ramp of 1°C/min, from 160°C to 200°C with ramp of 10°C/min. Retention times: $t_R(R)$ = 19.9 min, $t_R(S)$ = 20.4 min.[1]

(*R*)-1-(4-chlorophenyl)ethanol (**P6**)

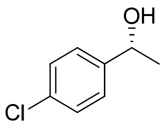

93% *ee* (*R*). ^{1}H NMR (400 MHz, $CDCl_3$) δ 7.34 – 7.27 (m, 4H, aromatic), 4.88 (q, *J* = 6.5 Hz, 1H, CH), 1.89 (br. s, 1H, OH), 1.47 (d, *J* = 6.5 Hz, 3H, CH_3) ppm. HPLC conditions: Kromasil 3-CelluCoat column, eluent: n-hexane/*i*PrOH 97/3, λ = 220 nm, flow rate: 0.5 mL/min. Retention times: $t_R(S)$ = 12.0 min, $t_R(R)$ = 13.3 min.[4]

(*R*)-1-(4-bromophenyl)ethanol (**P7**)

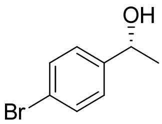

85% *ee* (*R*). ^{1}H NMR (400 MHz, $CDCl_3$) δ 7.49 – 7.40 (m, 2H, aromatic), 7.26 – 7.21 (m, 2H, aromatic), 4.85 (q, *J* = 6.4 Hz, 1H, CH), 1.72 (br. s, 1H, OH), 1.45 (d, *J* = 6.5 Hz, 3H, CH_3) ppm. HPLC conditions: Kromasil 3-CelluCoat column, eluent: n-hexane/*i*PrOH 97/3, λ = 220 nm, flow rate: 0.5 mL/min. Retention times: $t_R(S)$ = 12.5 min, $t_R(R)$ = 13.8 min.[5]

(*R*)-1-(4-(trifluoromethyl)phenyl)ethanol (**P8**)

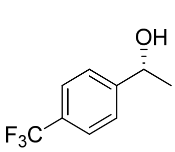

81% *ee* (*R*). ^{1}H NMR (400 MHz, $CDCl_3$) δ 7.65 – 7.58 (m, 2H, aromatic), 7.51 – 7.46 (m, 2H, aromatic), 4.96 (q, *J* = 6.5 Hz, 1H, CH), 1.92 (br. s, 1H, OH), 1.50 (d, *J* = 6.5 Hz, 3H, CH_3) ppm. GC conditions: Supelco β-DEX™ 225, L x I.D. 30 m x 0.25 mm, df 0.25 μm, carrier gas: N_2 (gas flow rate: 1.1 mL/min), injection temperature: 250°C, detector temperature: 250°C. Temperature profile: 80°C for 10 min, from 80°C to 120°C with ramp of 3°C/min, 120°C for 10 min, from 120°C to 180°C with ramp of 10°C/min, 180°C for 15 min. Retention times: $t_R(R)$ = 30.3 min, $t_R(S)$ = 31.1 min.[6]

(*R*)-1-(3-methoxyphenyl)ethanol (**P9**)

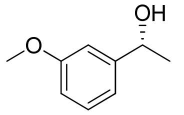

96% *ee* (*R*). ^{1}H NMR (400 MHz, $CDCl_3$) δ 7.28 – 7.19 (m, 1H, aromatic), 6.95 – 6.89 (m, 2H, aromatic), 6.81 – 6.77 (m, 1H, aromatic), 4.84 (q, J = 6.5 Hz, 1H, CH), 3.79 (s, 3H, OCH_3), 1.94 (br. s, 1H, OH), 1.47 (d, J = 6.5 Hz, 3H, CH_3) ppm. GC conditions: Supelco β-DEX™ 225, L x I.D. 30 m x 0.25 mm, df 0.25 μm, carrier gas: N_2 (gas flow rate: 2 mL/min), injection temperature: 250°C, detector temperature: 250°C. Temperature profile: from 100°C to 160°C with ramp of 1°C/min, from 160°C to 200°C with ramp of 10°C/min. Retention times: $t_R(R)$ = 26.8 min, $t_R(S)$ = 28.1 min.[1]

(*R*)-1-(3-(trifluoromethyl)phenyl)ethanol (**P10**)

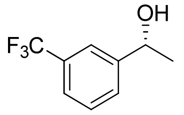

89% *ee* (*R*). ^{1}H NMR (400 MHz, $CDCl_3$) δ 7.71 – 7.66 (m, 1H, aromatic), 7.63 – 7.53 (m, 2H, aromatic), 7.53 – 7.44 (m, 1H, aromatic), 5.00 (q, J = 6.5 Hz, 1H, CH), 1.89 (br. s, 1H, OH), 1.55 (d, J = 6.5 Hz, 3H, CH_3). GC conditions: Supelco β-DEX™ 225, L x I.D. 30 m x 0.25 mm, df 0.25 μm, carrier gas: N_2 (gas flow rate: 2 mL/min), injection temperature: 250°C, detector temperature: 250°C. Temperature profile: 80°C for 10 min, from 80°C to 120°C with ramp of 5°C/min, 120°C for 10 min, from 120°C to 180°C with ramp of 10°C/min, 180°C for 15 min. Retention times: $t_R(R)$ = 20.0 min, $t_R(S)$ = 20.7 min.[1]

(*R*)-1-(3,5-bis(trifluoromethyl)phenyl)ethanol (**P11**)

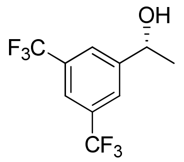

70% *ee* (*R*). ^{1}H NMR (400 MHz, $CDCl_3$) δ 7.87 (br. s, 2H, aromatic), 7.81 (br. s, 1H, aromatic), 5.07 (q, J = 6.5 Hz, 1H, CH), 1.86 (br. s, 1H, OH), 1.57 (d, J = 6.5 Hz, 3H, CH_3) ppm. GC conditions: Supelco β-DEX™ 225, L x I.D. 30 m x 0.25 mm, df 0.25 μm, carrier gas: N_2 (gas flow rate: 2 mL/min), injection temperature: 250°C, detector temperature: 250°C. Temperature profile: 80°C for 10 min, from 80°C to 120°C with ramp of 5°C/min, 120°C for 10 min, from 120°C to 180°C with ramp of 10°C/min, 180°C for 15 min. Retention times: $t_R(S)$ = 17.7 min, $t_R(R)$ = 18.0 min.[6]

(*R*)-1-(naphthalen-1-yl)ethanol (**P12**)

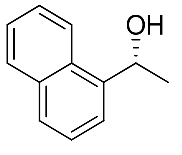

91% *ee* (*R*). ^{1}H NMR (400 MHz, $CDCl_3$) δ 8.14 – 8.06 (m, 1H, aromatic), 7.90 – 7.83 (m, 1H, aromatic), 7.80 – 7.73 (m, 1H, aromatic), 7.70 – 7.61 (m, 1H, aromatic), 7.55 – 7.42 (m, 3H, aromatic), 5.67 (q, J = 6.5 Hz, 1H, CH), 1.87 (br. s, 1H, OH), 1.66 (d, J = 6.5 Hz, 3H, CH_3) ppm. HPLC conditions: Kromasil 3-CelluCoat

column, eluent: n-hexane/*i*PrOH 85/15, λ = 220 nm, flow rate: 0.5 mL/min. Retention times: t_R(*S*) = 8.1 min, t_R(*R*) = 11.8 min.[2]

(*R*)-1-(naphthalen-2-yl)ethanol (**P13**)

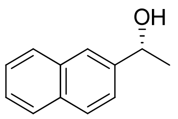

94% *ee* (*R*). ^{1}H NMR (400 MHz, $CDCl_3$) δ 7.89 – 7.81 (m, 4H, aromatic), 7.55 – 7.44 (m, 3H, aromatic), 5.09 (q, *J* = 6.5 Hz, 1H, CH), 1.86 (br. s, 1H, OH), 1.61 (d, *J* = 6.5 Hz, 3H, CH_3) ppm. HPLC conditions: Lux® Cellulose-3 column, eluent: n-hexane/*i*PrOH 85/15, λ = 220 nm, flow rate: 0.5 mL/min. Retention times: t_R(*S*) = 11.5 min, t_R(*R*) = 14.1 min.[7]

(*R*)-1-phenylpropan-1-ol (**P14**)

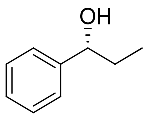

97% *ee* (*R*). ^{1}H NMR (400 MHz, $CDCl_3$) δ 7.39 – 7.34 (m, 4H, aromatic), 7.33 – 7.26 (m, 1H, aromatic), 4.62 (dd, *J* = 7.0, 6.2 Hz, 1H, CH), 1.92 – 1.66 (m, 3H, CH_2 and OH, overlapped), 0.95 (t, *J* = 7.4 Hz, 3H, CH_3) ppm. HPLC conditions: Kromasil 3-CelluCoat column, eluent: n-hexane/*i*PrOH 98/2, λ = 220 nm, flow rate: 0.5 mL/min. Retention times: t_R(*R*) = 14.5 min, t_R(*S*) = 17.0 min.[2]

(*R*)-1-phenylbutan-1-ol (**P15**)

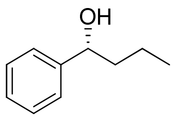

96% *ee* (*R*). ^{1}H NMR (400 MHz, $CDCl_3$) δ 7.36 – 7.30 (m, 4H, aromatic), 7.29 – 7.23 (m, 1H, aromatic), 4.66 (dd, *J* = 7.5, 5.8 Hz, 1H, CH), 1.85 (br. s, 1H, OH), 1.83 – 1.72 (m, 1H, diast. C*H*H), 1.72 – 1.60 (m, 1H, diast. CH*H*), 1.49 – 1.37 (m, 1H, diast. C*H*H), 1.36 – 1.22 (m, 1H, diast. CH*H*), 0.92 (t, *J* = 7.4 Hz, 1H, CH_3) ppm. HPLC conditions: Lux® Cellulose-3 column, eluent: n-hexane/*i*PrOH 97/3, λ = 220 nm, flow rate: 0.5 mL/min. Retention times: t_R(*S*) = 11.4 min, t_R(*R*) = 12.0 min.[7]

(*R*)-1-phenylpentan-1-ol (**P16**)

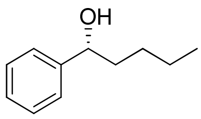

97% *ee* (*R*). ^{1}H NMR (400 MHz, Acetone-d_6): δ = 7.38 – 7.33 (m, 2H, aromatic), 7.33 – 7.27 (m, 2H, aromatic), 7.23 – 7.18 (m, 1H, aromatic), 4.69 – 4.57 (m, 1H, CH), 4.09 (d, *J* = 4.2 Hz, 1H, OH), 1.81 – 1.56 (m, 2H), 1.47 – 1.19 (m, 4H), 0.87 (t, *J* = 7.1 Hz, 3H, CH_3) ppm. HPLC conditions: Kromasil 3-CelluCoat column, n-hexane/*i*PrOH 99.5/0.5 eluent, λ = 220 nm, 0.5 mL/min flow rate. Retention times: t_R(*R*) = 35.5 min, t_R(*S*) = 39.5 min.[8]

(R)-cyclohexyl(phenyl)methanol (**P17**)

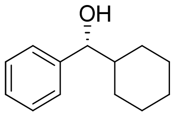

94% *ee* (R). ^{1}H NMR (400 MHz, Acetone-d_6) δ 7.32 – 7.25 (m, 4H, aromatic), 7.23 – 7.18 (m, 1H, aromatic), 4.35 (dd, J = 6.6, 4.2 Hz, 1H, CHOH), 4.02 (d, J = 4.1 Hz, 1H, OH), 1.98 – 1.91 (m, 1H), 1.77 – 1.69 (m, 1H), 1.69 – 1.49 (m, 3H), 1.45 – 1.35 (m, 1H), 1.26 – 0.95 (m, 5H) ppm. HPLC conditions: Kromasil 3-CelluCoat column, eluent: n-hexane/*i*PrOH 97/3, λ = 220 nm, flow rate: 0.5 mL/min. Retention times: t_R(S) = 10.5 min, t_R(R) = 12.2 min.[3]

(R)-3-methyl-1-phenylbutan-1-ol (**P18**)

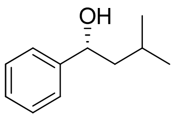

97% *ee* (R). ^{1}H NMR (400 MHz, Aceton-d_6): δ = 7.39 – 7.33 (m, 2H, aromatic), 7.33 – 7.27 (m, 2H, aromatic), 7.24 – 7.17 (m, 1H, aromatic), 4.71 (dt, J = 9.1, 4.7 Hz, 1H), 4.06 (dd, J = 4.4, 0.7 Hz, 1H), 1.84 – 1.72 (m, 1H), 1.65 (ddd, J = 13.6, 8.8, 5.6 Hz, 1H), 1.47 – 1.37 (m, 1H), 0.94 (d, J = 6.6 Hz, 3H, CH_3), 0.93 (d, J = 6.7 Hz, 3H, CH_3) ppm. HPLC conditions: Kromasil 3-CelluCoat column, n-hexane/*i*PrOH 99/1 eluent, λ = 220 nm, 0.5 mL/min flow rate. Retention times: t_R(S) = 18.0 min, t_R(R) = 21.2 min.[9]

(R)-2-methyl-1-phenylpropan-1-ol (**P19**)

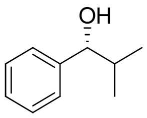

98% *ee* (R). ^{1}H NMR (400 MHz, Aceton-d_6): δ = 7.35 – 7.27 (m, 4H, aromatic), 7.23 – 7.18 (m, 1H, aromatic), 4.36 (dd, J = 6.3, 4.2 Hz, 1H), 4.07 (d, J = 4.1 Hz, 1H), 1.95 – 1.81 (m, 1H), 0.93 (d, J = 6.7 Hz, 3H, CH_3), 0.80 (d, J = 6.8 Hz, 3H, CH_3) ppm. HPLC conditions: Kromasil 3-CelluCoat column, n-hexane/*i*PrOH 98/2 eluent, λ = 220 nm, 0.5 mL/min flow rate. Retention times: t_R(S) = 11.8 min, t_R(R) = 13.9 min.[2]

(R)-1-(4-chlorophenyl)butan-1-ol (**P20**)

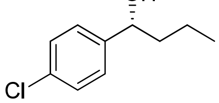

92% *ee* (R). ^{1}H NMR (400 MHz, Aceton-d_6): δ = 7.40 – 7.35 (m, 2H, aromatic), 7.35 – 7.30 (m, 2H, aromatic), 4.67 (dt, J = 7.7, 4.9 Hz, 1H), 4.31 – 4.16 (m, 1H), 1.75 – 1.51 (m, 2H), 1.51 – 1.22 (m, 2H), 0.90 (t, J = 7.4 Hz, 3H, CH_3) ppm. HPLC conditions: Lux® Cellulose-3 column, n-hexane/*i*PrOH 98/2 eluent, λ = 220 nm, 0.5 mL/min flow rate. Retention times: t_R(S) = 12.8 min, t_R(R) = 13.6 min.[10] The configuration of the main product (R) has been tentatively assigned based on the (R)-selectivity of the catalyst.

(*R*)-1-cyclopropylethanol (**P21**)

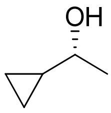

26% *ee* (*R*). ^{1}H NMR (400 MHz, Aceton-d$_6$): δ = 3.44 (d, *J* = 4.1 Hz, 1H), 3.14 – 3.06 (m, 1H), 1.17 (d, *J* = 6.2 Hz, 3H, CH_3), 0.86 – 0.76 (m, 1H), 0.39 – 0.33 (m, 2H), 0.30 – 0.22 (m, 1H), 0.18 – 0.11 (m, 1H) ppm. GC separation conditions: Supelco β-DEX™ 225, L x I.D. 30 m x 0.25 mm, df 0.25 µm, carrier gas: N_2 (gas flow rate: 2 mL/min), injection temperature: 250°C, detector temperature: 250°C. Temperature profile: 50°C for 5 min, from 50°C to 80°C with ramp of 0.5°C/min. Retention times: t_R(*S*) = 17.4 min, t_R(*R*) = 18.6 min.[11]

(*S*)-3,3-dimethylbutan-2-ol (**P22**)

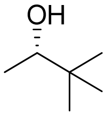

25% *ee* (*S*). ^{1}H NMR (400 MHz, Aceton-d$_6$): δ = 3.32 (q, *J* = 6.4 Hz, 1H), 1.05 (d, *J* = 6.4 Hz, 3H, CH_3), 0.86 (s, 9H, $C(CH_3)_3$) ppm. GC separation conditions: Supelco β-DEX™ 225, L x I.D. 30 m x 0.25 mm, df 0.25 µm, carrier gas: N_2 (gas flow rate: 2 mL/min), injection temperature: 250°C, detector temperature: 250°C. Temperature profile: from 50°C to 80°C with ramp of 1°C/min. Retention times: t_R(*S*) = 6.6 min, t_R(*R*) = 7.2 min, t_R(substrate) = 7.5 min.[11]

3. GC and HPLC chromatograms

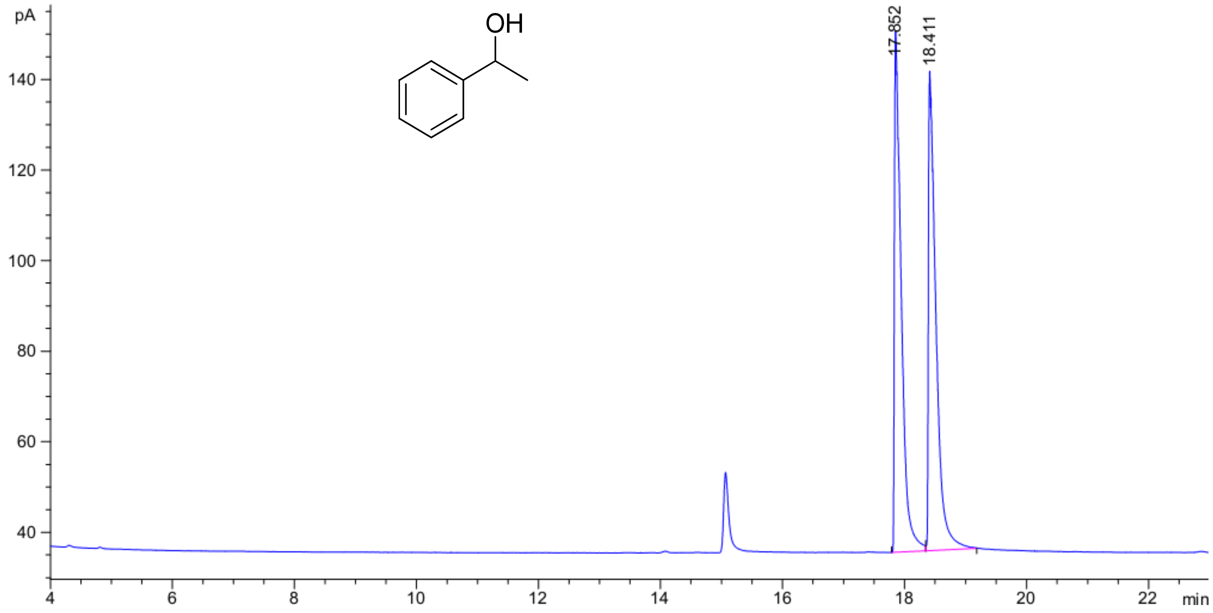

Peak #	RetTime [min]	Type	Width [min]	Area [pA*s]	Height [pA]	Area %
1	17.852	BV	0.1007	890.65765	115.14223	49.55752
2	18.411	VB	0.1084	906.56232	105.87869	50.44248
Totals :				1797.21997	221.02092	

Figure S25. Chromatogram of racemic 1-phenylethanol

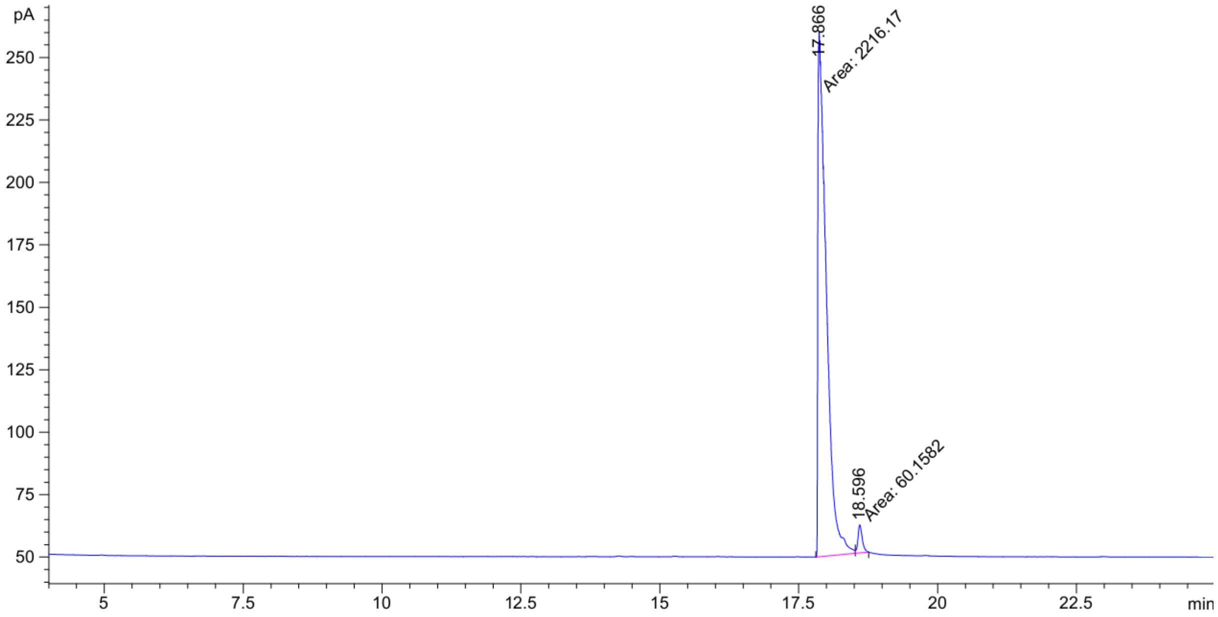

Peak #	RetTime [min]	Type	Width [min]	Area [pA*s]	Height [pA]	Area %
1	17.866	MF	0.1753	2216.17480	210.66841	97.35723
2	18.596	FM	0.0885	60.15823	11.32303	2.64277
Totals :				2276.33303	221.99144	

Figure S26. Chromatogram of optically active 1-phenylethanol (**P1**)

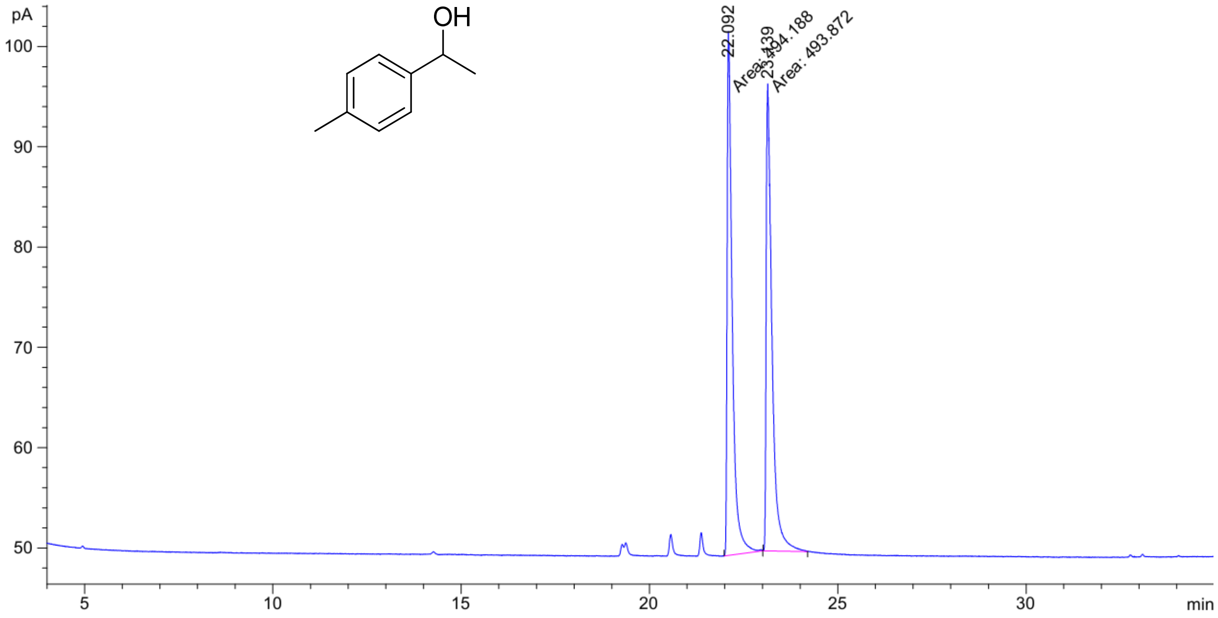

Peak #	RetTime [min]	Type	Width [min]	Area [pA*s]	Height [pA]	Area %
1	22.092	MM	0.1576	494.18759	52.27767	50.01599
2	23.139	MM	0.1766	493.87167	46.59840	49.98401
Totals :				988.05927	98.87607	

Figure S27. Chromatogram of racemic 1-(p-tolyl)ethanol

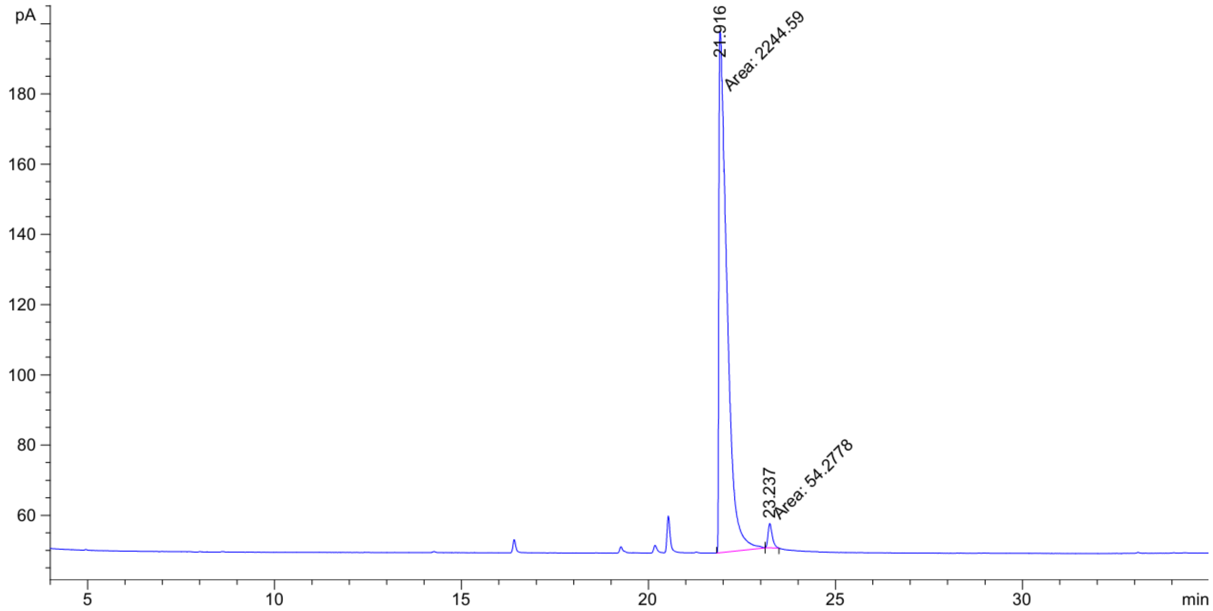

Peak #	RetTime [min]	Type	Width [min]	Area [pA*s]	Height [pA]	Area %
1	21.916	MM	0.2519	2244.59326	148.52371	97.63894
2	23.237	MM	0.1298	54.27782	6.96696	2.36106
Totals :				2298.87108	155.49067	

Figure S28. Chromatogram of optically active 1-(p-tolyl)ethanol (**P2**)

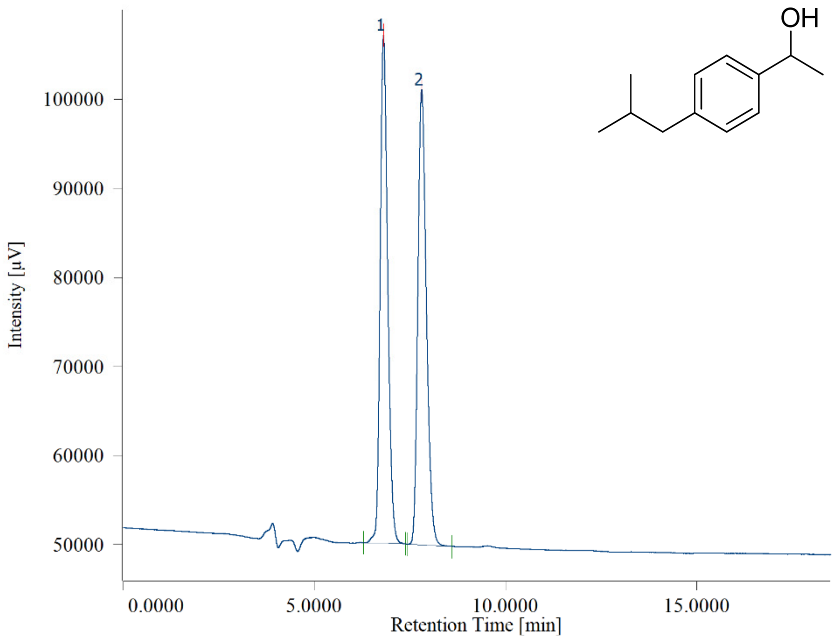

Peak Information

#	tR [min]	Area [μV·sec]	Height [μV]	Area%	Height%
1	6.806	782444	56904	49.762	52.732
2	7.801	789929	51008	50.238	47.268

Figure S29. Chromatogram of racemic 1-(4-isobutylphenyl)ethanol

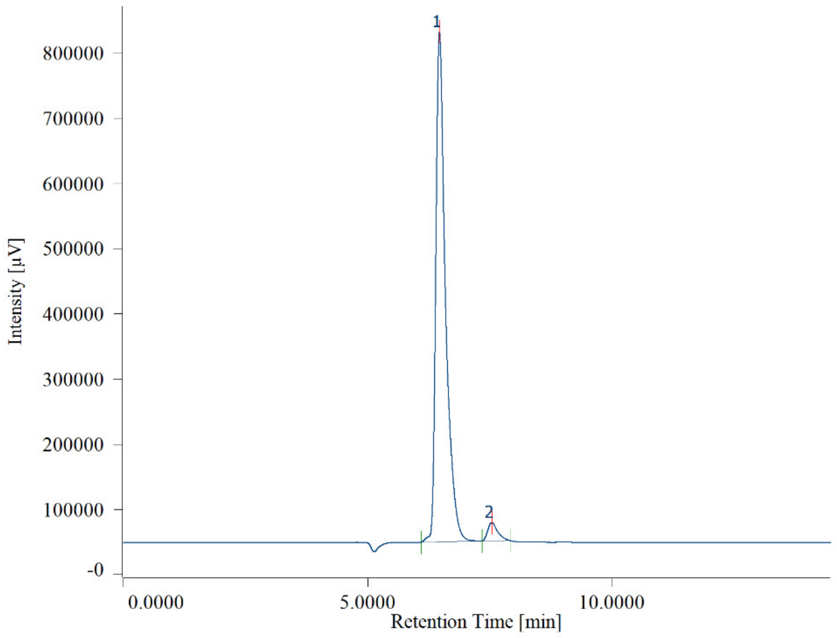

Peak Information

#	tR [min]	Area [μV·sec]	Height [μV]	Area%	Height%
1	6.464	10805355	781615	96.561	96.552
2	7.530	384792	27909	3.439	3.448

Figure S30. Chromatogram of optically active 1-(4-isobutylphenyl)ethanol (**P3**)

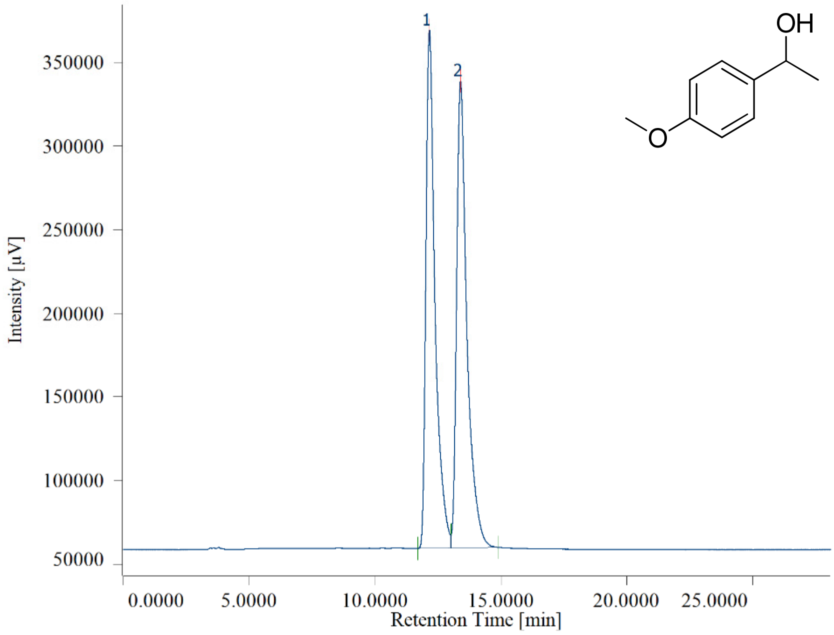

Peak Information

#	tR [min]	Area [µV·sec]	Height [µV]	Area%	Height%
1	12.151	7818286	309378	49.624	52.589
2	13.386	7936775	278912	50.376	47.411

Figure S31. Chromatogram of racemic 1-(4-methoxyphenyl)ethanol

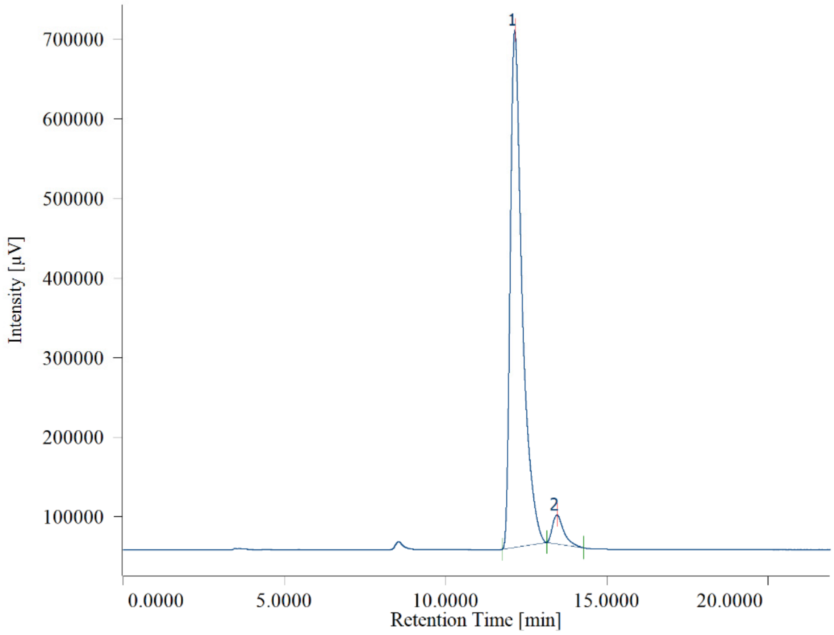

Peak Information

#	tR [min]	Area [µV·sec]	Height [µV]	Area%	Height%
1	12.130	16710219	649089	95.004	94.676
2	13.433	878742	36502	4.996	5.324

Figure S32. Chromatogram of optically active 1-(4-methoxyphenyl)ethanol (**P4**)

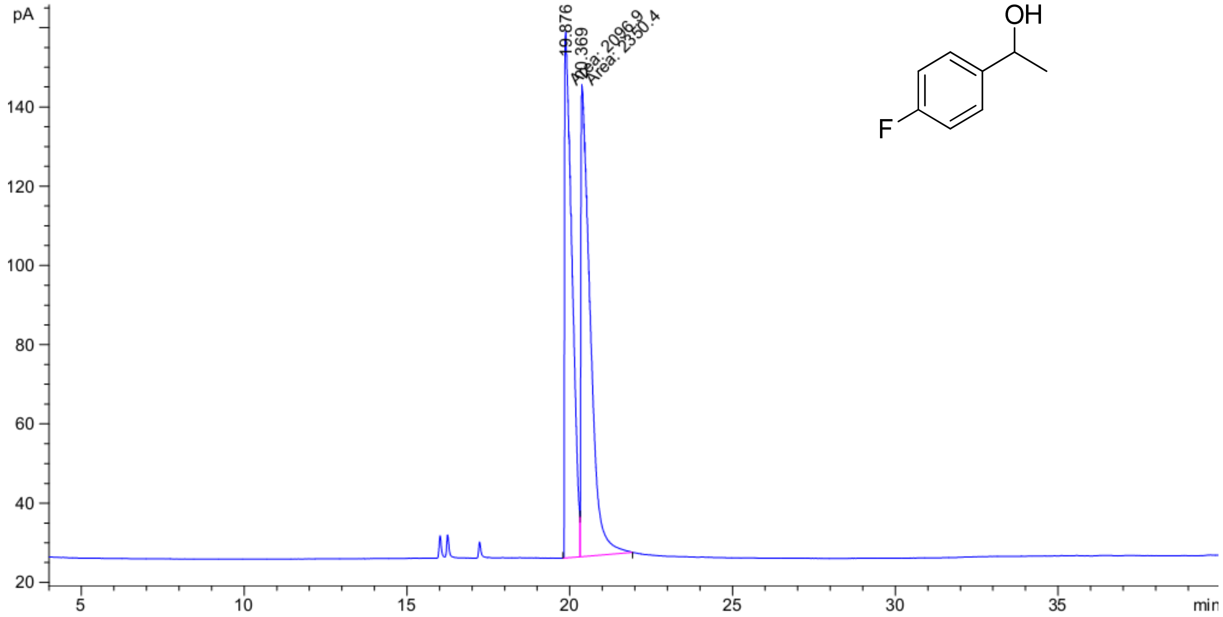

Peak #	RetTime [min]	Type	Width [min]	Area [pA*s]	Height [pA]	Area %
1	19.876	MF	0.2629	2096.90112	132.94525	47.14992
2	20.369	FM	0.3287	2350.40430	119.18733	52.85008
Totals :				4447.30542	252.13258	

Figure S33. Chromatogram of racemic 1-(4-fluorophenyl)ethanol

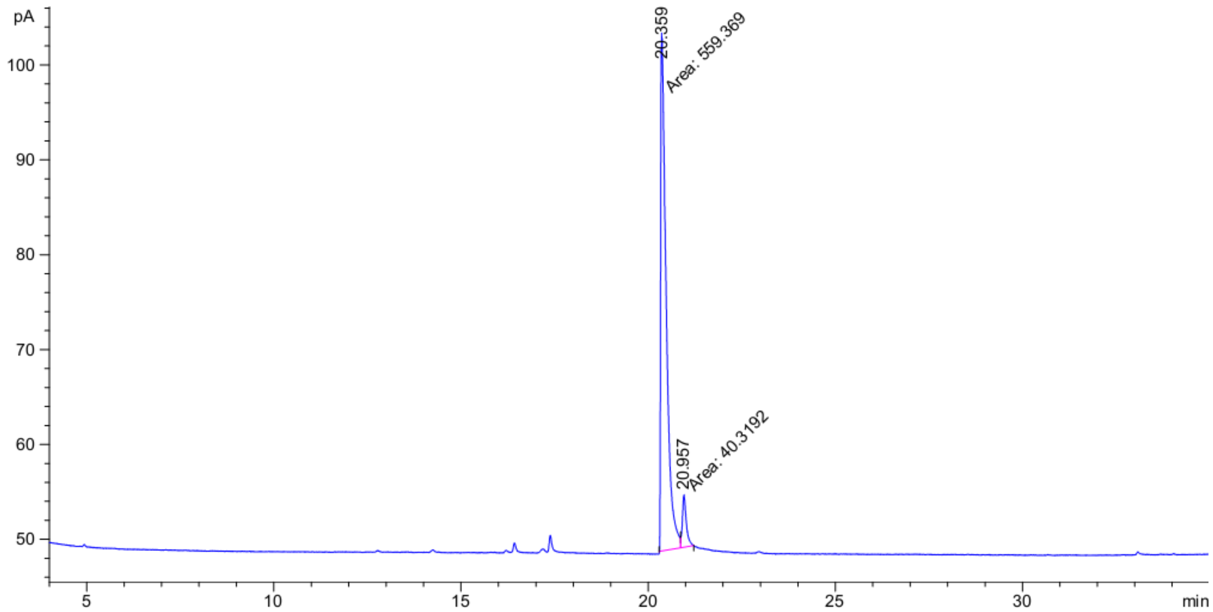

Peak #	RetTime [min]	Type	Width [min]	Area [pA*s]	Height [pA]	Area %
1	20.359	MF	0.1706	559.36853	54.64715	93.27663
2	20.957	FM	0.1216	40.31919	5.52690	6.72337
Totals :				599.68773	60.17405	

Figure S34. Chromatogram of optically active 1-(4-fluorophenyl)ethanol (**P5**)

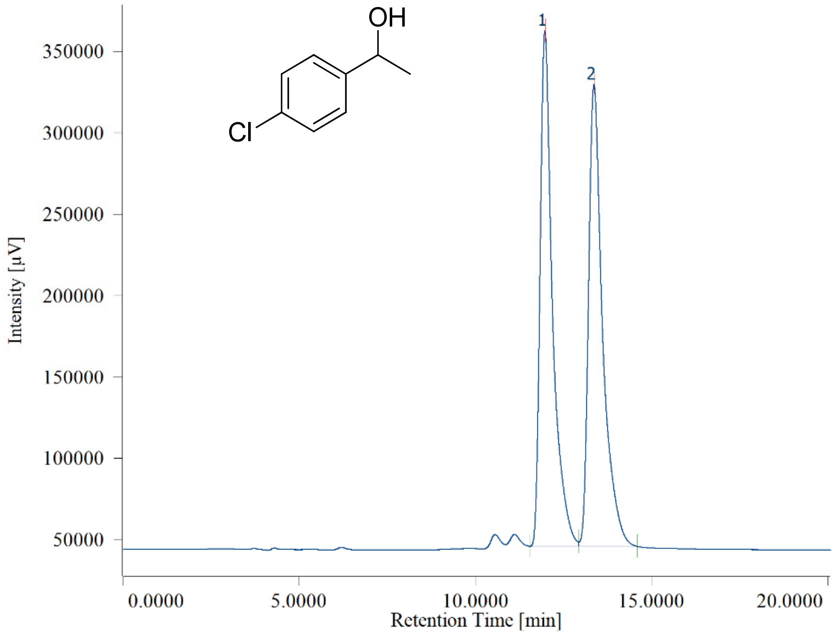

Peak Information

#	tR [min]	Area [µV·sec]	Height [µV]	Area%	Height%
1	11.957	7757593	316715	49.692	52.740
2	13.349	7853658	283810	50.308	47.260

Figure S35. Chromatogram of racemic 1-(4-chlorophenyl)ethanol

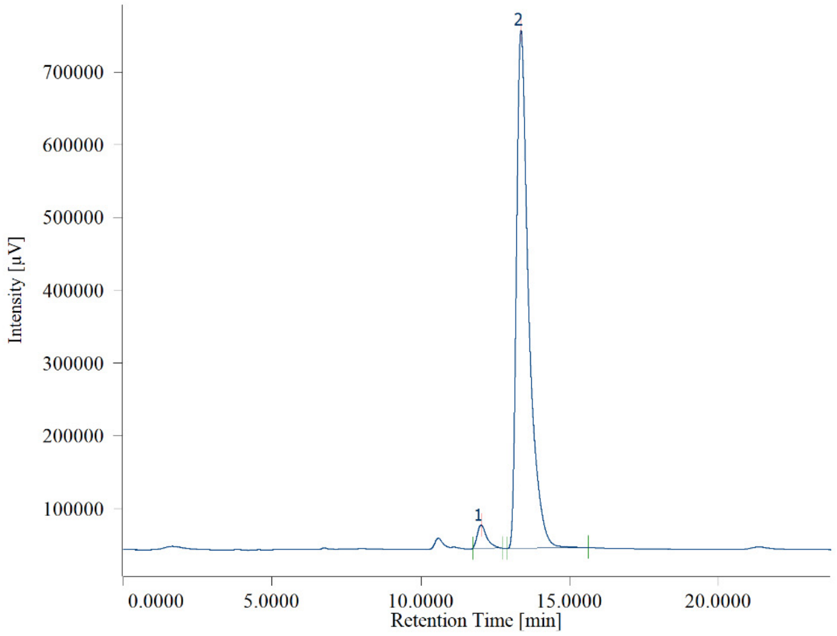

Peak Information

#	tR [min]	Area [µV·sec]	Height [µV]	Area%	Height%
1	12.019	701687	31969	3.345	4.303
2	13.359	20277454	710971	96.655	95.697

Figure S36. Chromatogram of optically active 1-(4-chlorophenyl)ethanol (**P6**)

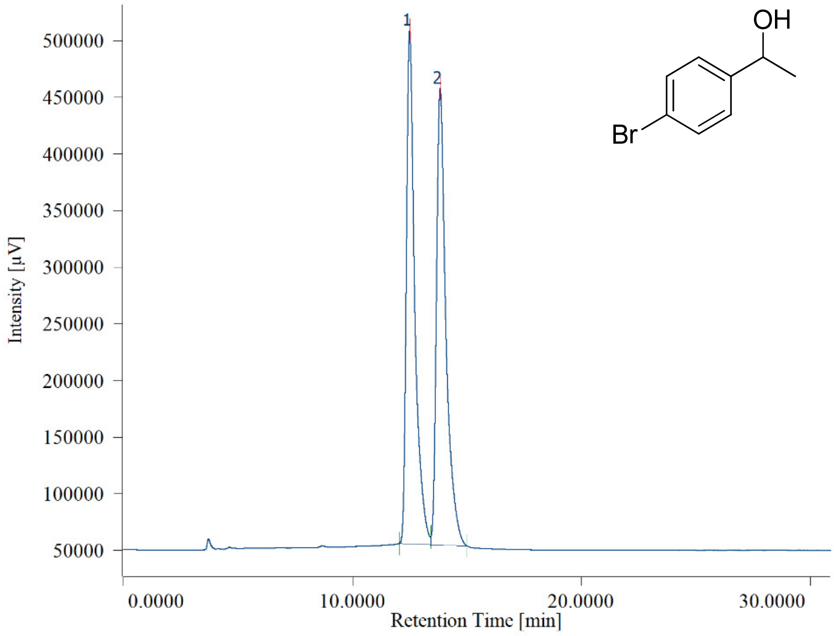

Peak Information

#	tR [min]	Area [μV·sec]	Height [μV]	Area%	Height%
1	12.495	11572042	453208	49.983	52.939
2	13.824	11579856	402887	50.017	47.061

Figure S37. Chromatogram of racemic 1-(4-bromophenyl)ethanol

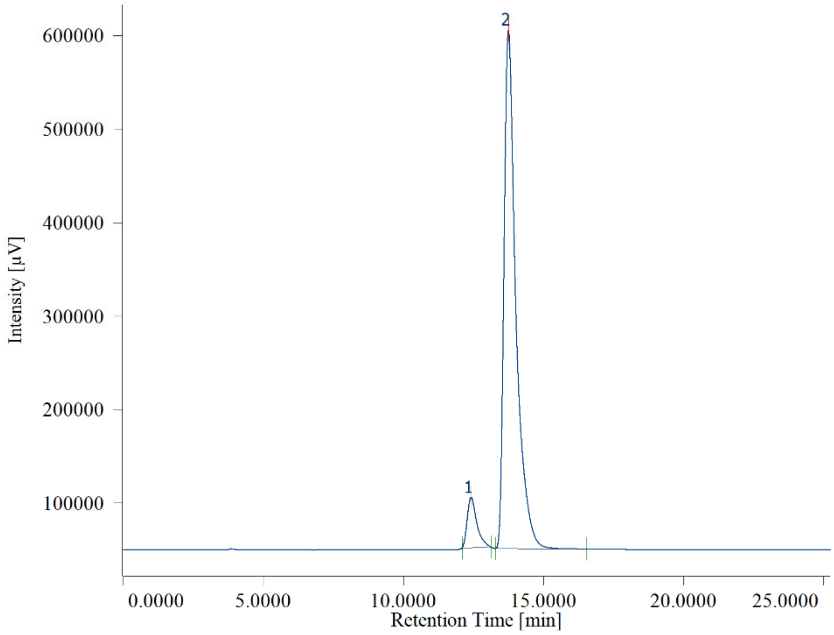

Peak Information

#	tR [min]	Area [μV·sec]	Height [μV]	Area%	Height%
1	12.406	1272162	53944	7.253	8.885
2	13.738	16266768	553229	92.747	91.115

Figure S38. Chromatogram of optically active 1-(4-bromophenyl)ethanol (**P7**)

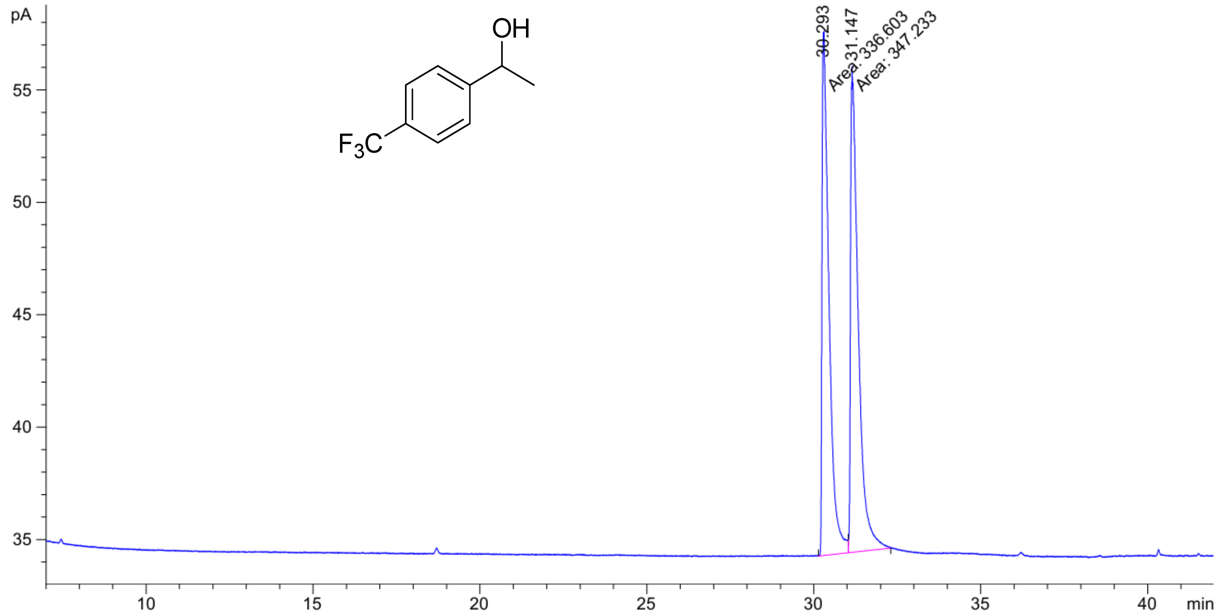

Peak #	RetTime [min]	Type	Width [min]	Area [pA*s]	Height [pA]	Area %
1	30.293	MF	0.2406	336.60349	23.31871	49.22283
2	31.147	FM	0.2671	347.23260	21.66545	50.77717
Totals :				683.83609	44.98416	

Figure S39. Chromatogram of racemic 1-(4-(trifluoromethyl)phenyl)ethanol

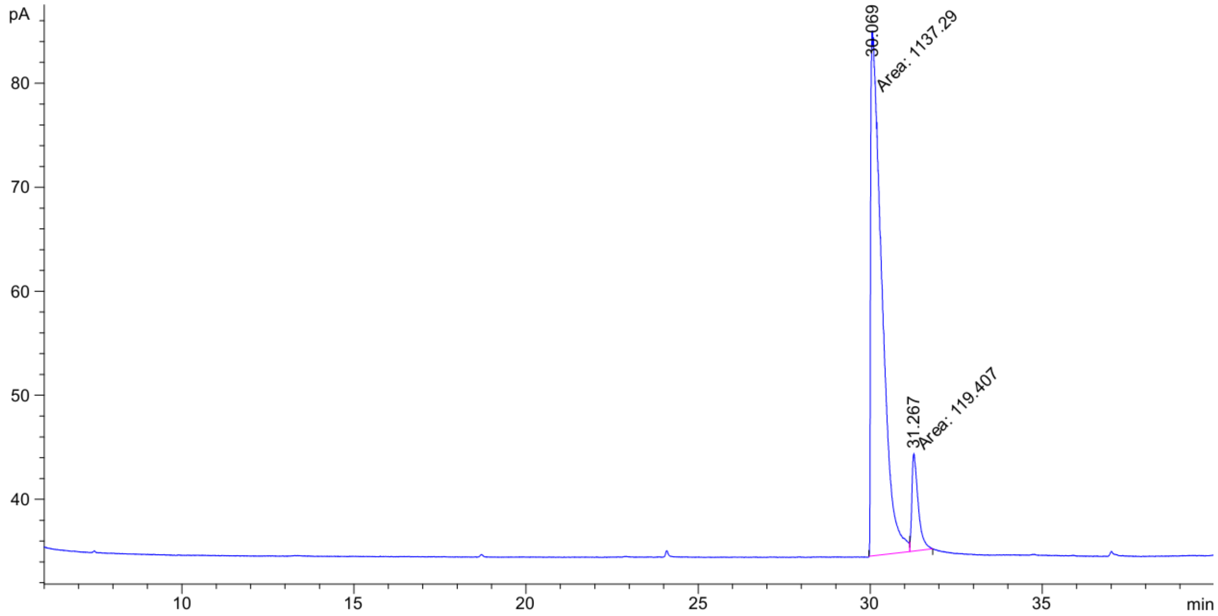

Peak #	RetTime [min]	Type	Width [min]	Area [pA*s]	Height [pA]	Area %
1	30.069	MF	0.3755	1137.29199	50.47402	90.49834
2	31.267	FM	0.2113	119.40726	9.41695	9.50166
Totals :				1256.69926	59.89097	

Figure S40. Chromatogram of optically active 1-(4-(trifluoromethyl)phenyl)ethanol (**P8**)

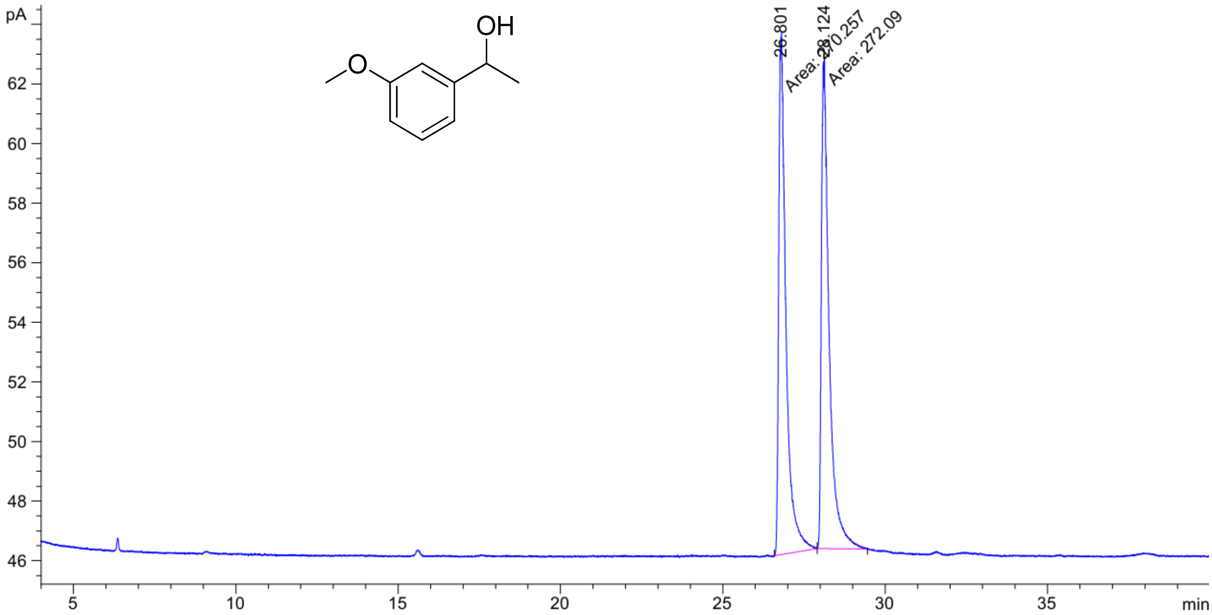

Peak #	RetTime [min]	Type	Width [min]	Area [pA*s]	Height [pA]	Area %
1	26.801	MM	0.2565	270.25674	17.55827	49.83099
2	28.124	MM	0.2739	272.09003	16.55460	50.16901
Totals :				542.34677	34.11287	

Figure S41. Chromatogram of racemic 1-(3-methoxyphenyl)ethanol

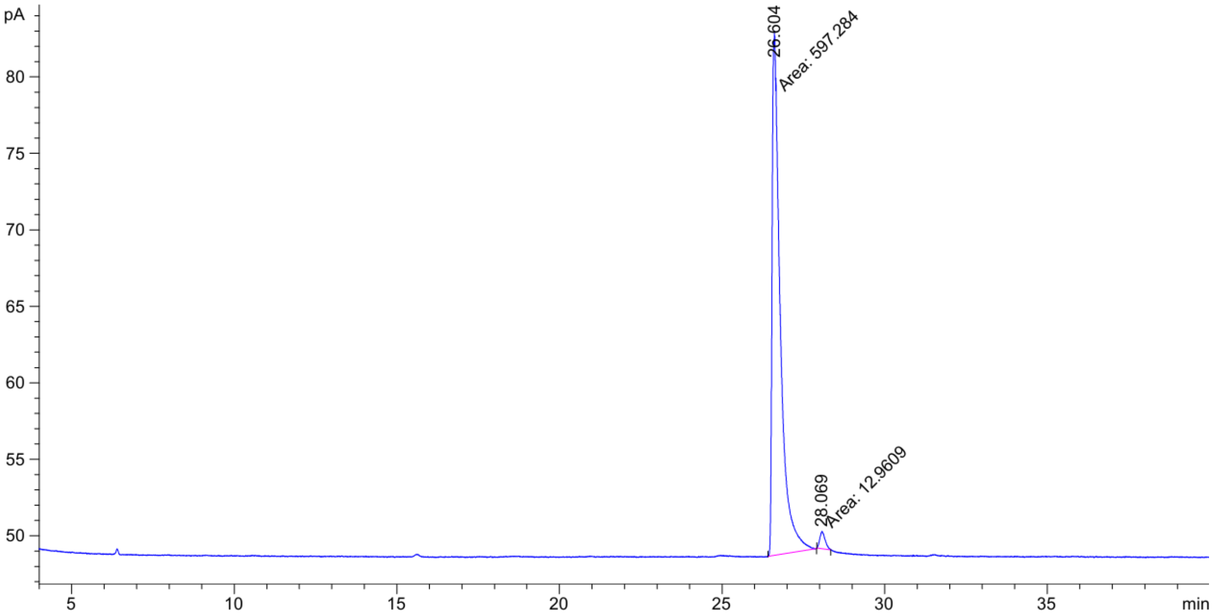

Peak #	RetTime [min]	Type	Width [min]	Area [pA*s]	Height [pA]	Area %
1	26.604	MM	0.2900	597.28436	34.32838	97.87611
2	28.069	MM	0.1881	12.96091	1.14856	2.12389
Totals :				610.24528	35.47695	

Figure S42. Chromatogram of optically active 1-(3-methoxyphenyl)ethanol (**P9**)

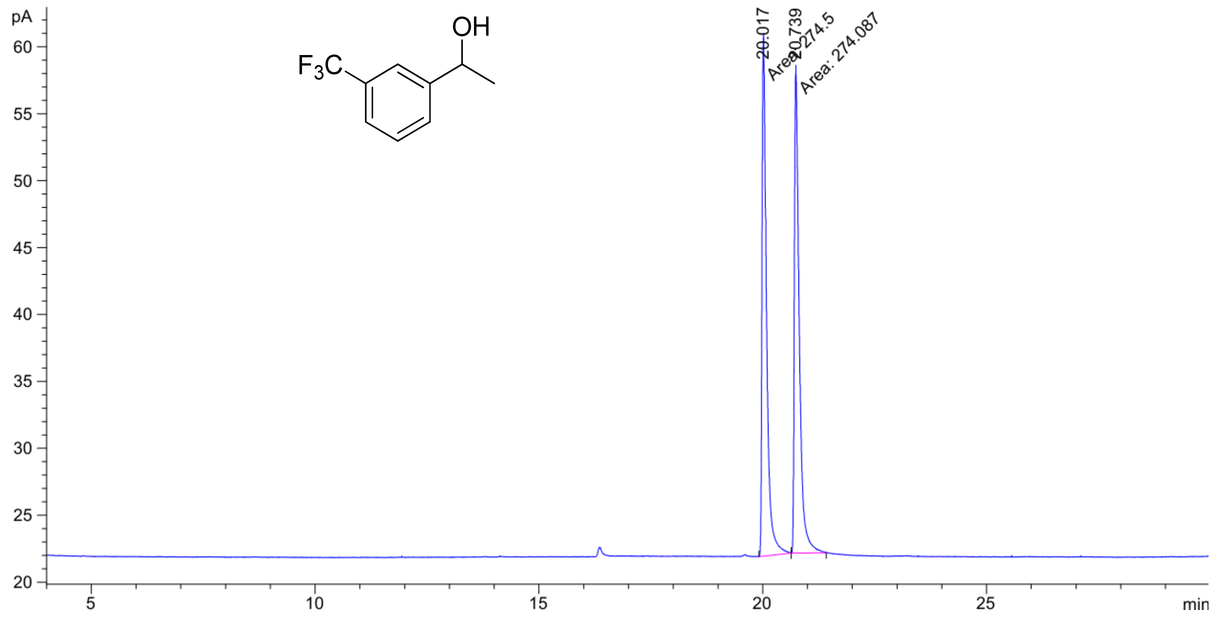

Peak #	RetTime [min]	Type	Width [min]	Area [pA*s]	Height [pA]	Area %
1	20.017	MM	0.1170	274.50009	39.09703	50.03762
2	20.739	MM	0.1252	274.08728	36.49136	49.96238
Totals :				548.58737	75.58839	

Figure S43. Chromatogram of racemic 1-(3-(trifluoromethyl)phenyl)ethanol

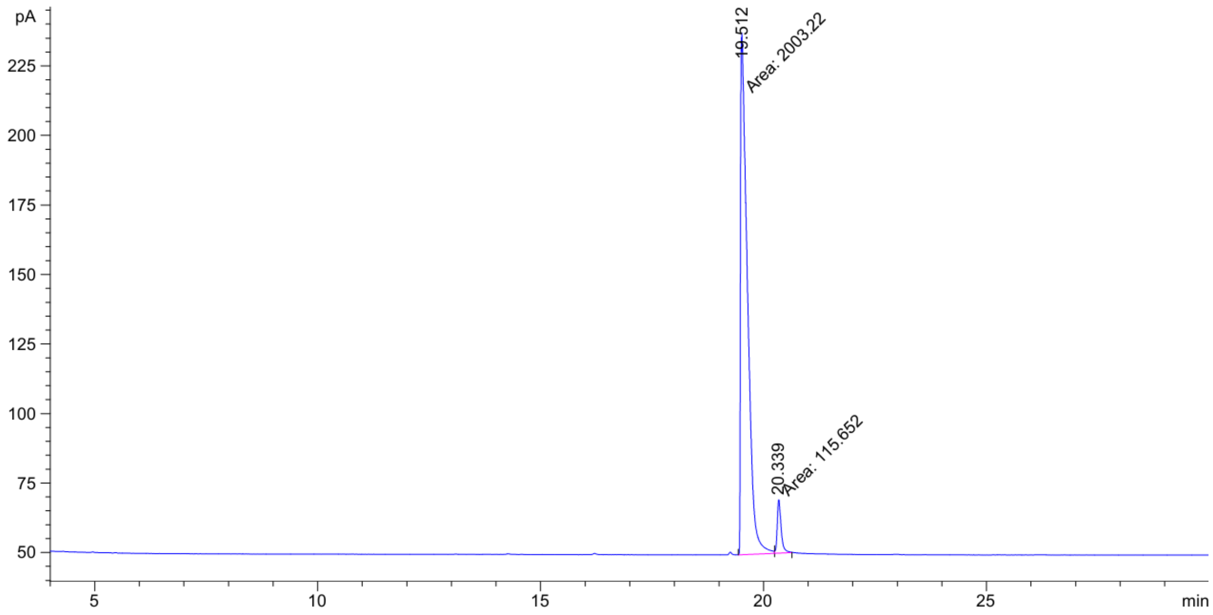

Peak #	RetTime [min]	Type	Width [min]	Area [pA*s]	Height [pA]	Area %
1	19.512	MF	0.1779	2003.22266	187.71419	94.54181
2	20.339	FM	0.1004	115.65224	19.20544	5.45819
Totals :				2118.87489	206.91963	

Figure S44. Chromatogram of optically active 1-(3-(trifluoromethyl)phenyl)ethanol (**P10**)

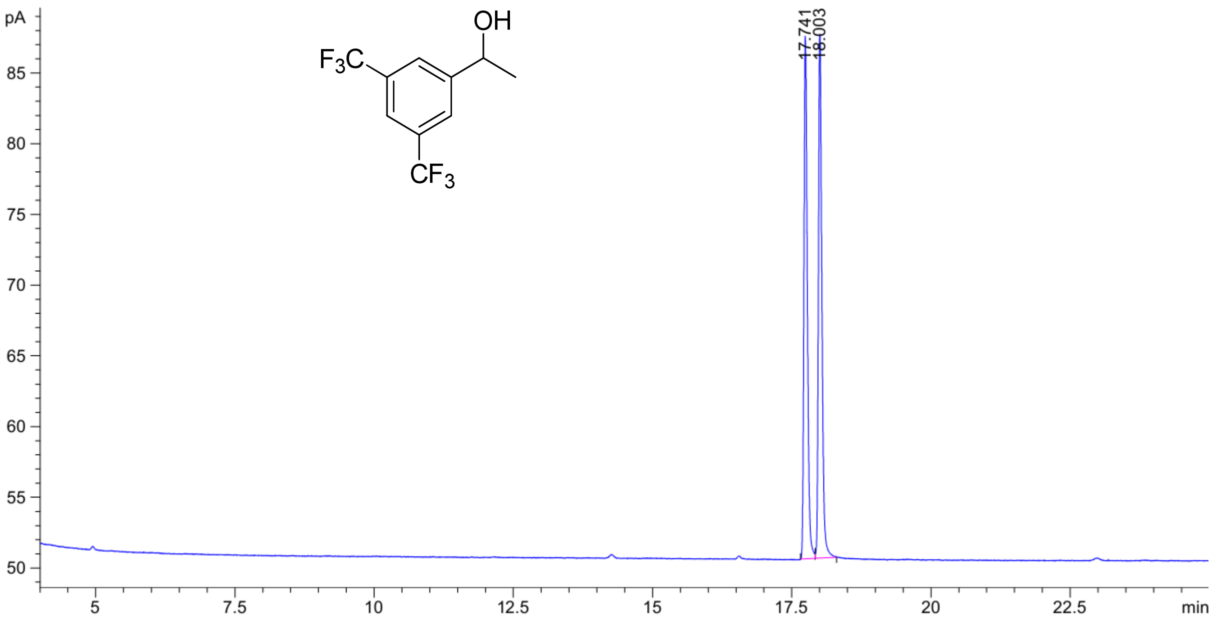

Peak #	RetTime [min]	Type	Width [min]	Area [pA*s]	Height [pA]	Area %
1	17.741	BV	0.0628	157.83784	36.59596	49.53066
2	18.003	VB	0.0649	160.82909	36.87909	50.46934
Totals :				318.66693	73.47505	

Figure S45. Chromatogram of racemic 1-(3,5-bis(trifluoromethyl)phenyl)ethanol

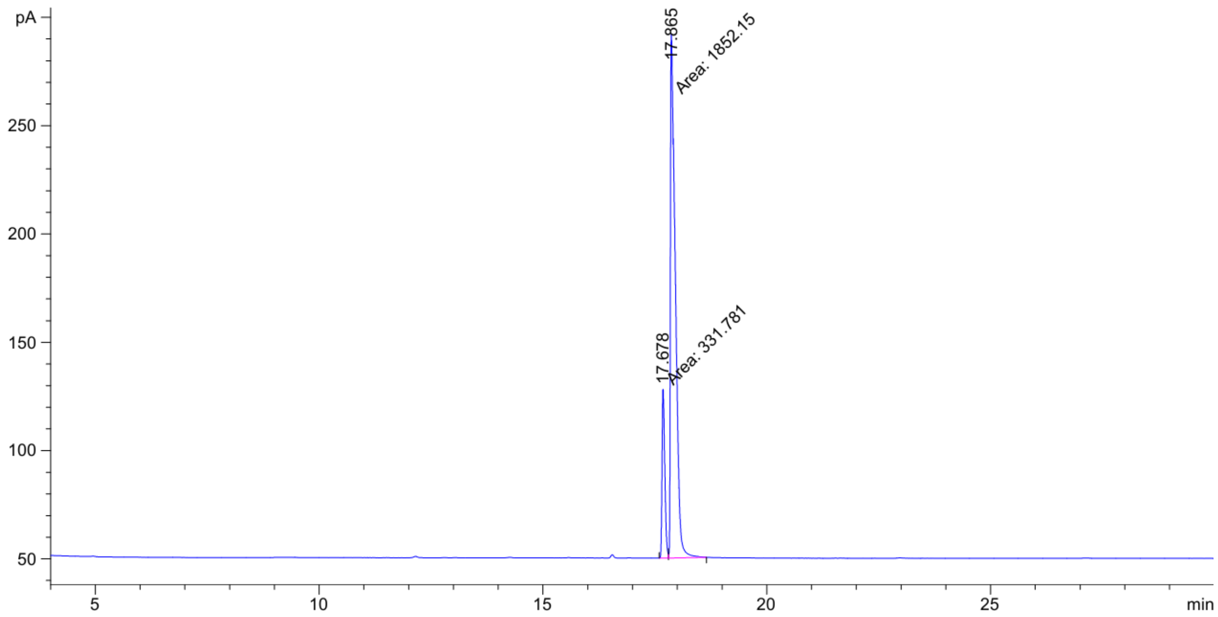

Peak #	RetTime [min]	Type	Width [min]	Area [pA*s]	Height [pA]	Area %
1	17.678	MF	0.0709	331.78067	78.00177	15.19191
2	17.865	FM	0.1275	1852.14929	242.13928	84.80809
Totals :				2183.92996	320.14105	

Figure S46. Chromatogram of optically active 1-(3,5-bis(trifluoromethyl)phenyl)ethanol (**P11**)

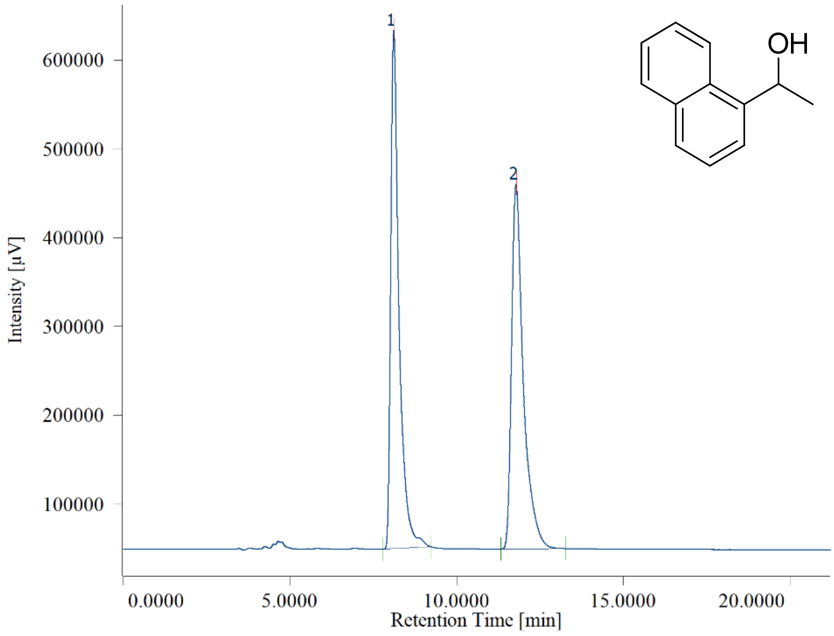

Peak Information

#	tR [min]	Area [µV·sec]	Height [µV]	Area%	Height%
1	8.113	10097369	582943	50.166	58.668
2	11.777	10030599	410692	49.834	41.332

Figure S47. Chromatogram of racemic 1-(naphthalen-1-yl)ethanol

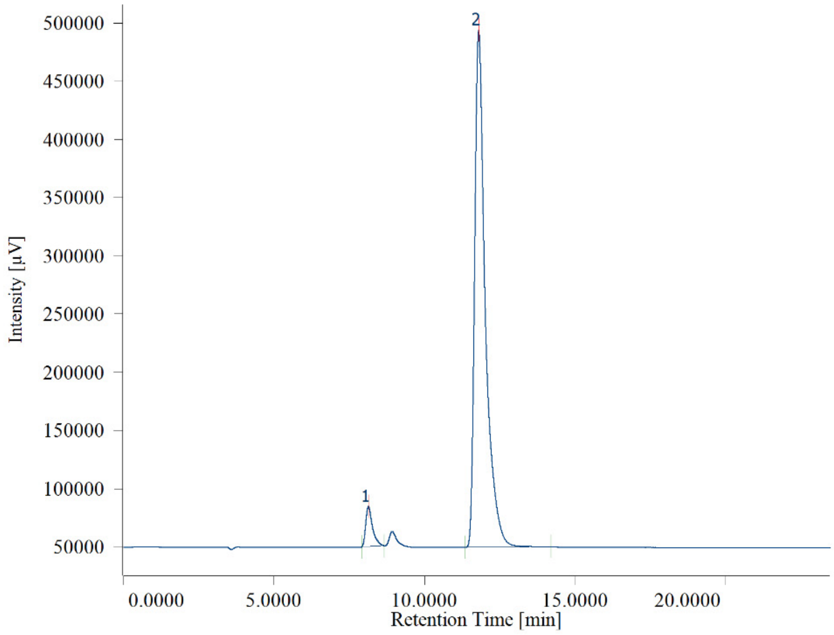

Peak Information

#	tR [min]	Area [µV·sec]	Height [µV]	Area%	Height%
1	8.129	539874	34469	4.700	7.205
2	11.790	10947362	443919	95.300	92.795

Figure S48. Chromatogram of optically active 1-(naphthalen-1-yl)ethanol (**P12**)

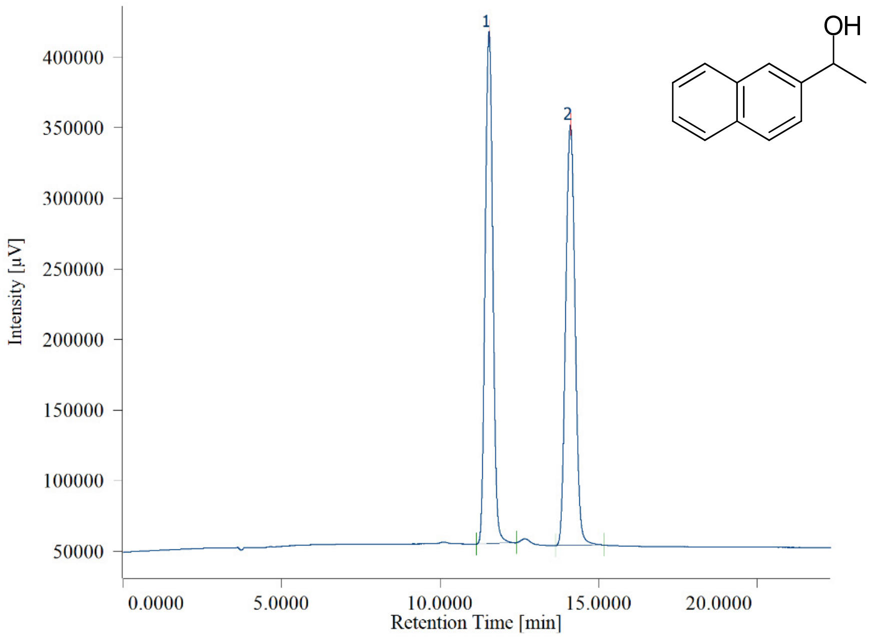

Peak Information

#	tR [min]	Area [µV·sec]	Height [µV]	Area%	Height%
1	11.534	6086908	362319	49.795	54.877
2	14.099	6137102	297917	50.205	45.123

Figure S49. Chromatogram of racemic 1-(naphthalen-2-yl)ethanol

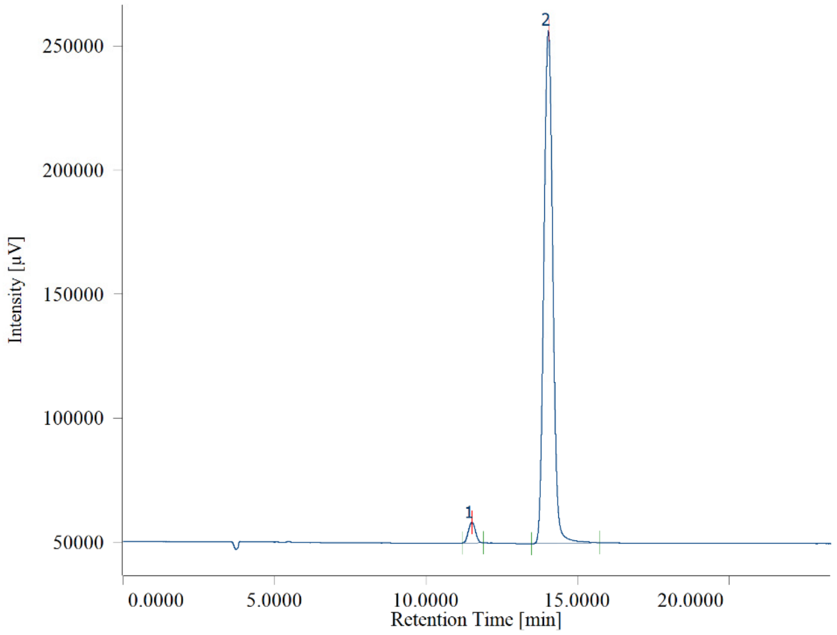

Peak Information

#	tR [min]	Area [µV·sec]	Height [µV]	Area%	Height%
1	11.509	133802	8263	3.054	3.849
2	14.032	4247819	206403	96.946	96.151

Figure S50. Chromatogram of optically active 1-(naphthalen-2-yl)ethanol (**P13**)

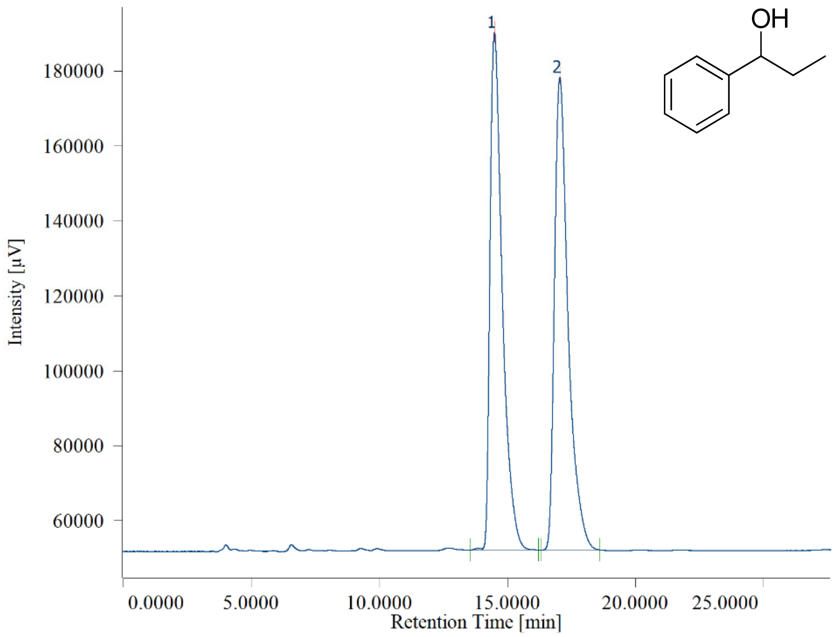

Peak Information

#	tR [min]	Area [µV·sec]	Height [µV]	Area%	Height%
1	14.482	4587664	137965	49.664	52.259
2	17.031	4649770	126038	50.336	47.741

Figure S51. Chromatogram of racemic 1-phenylpropan-1-ol

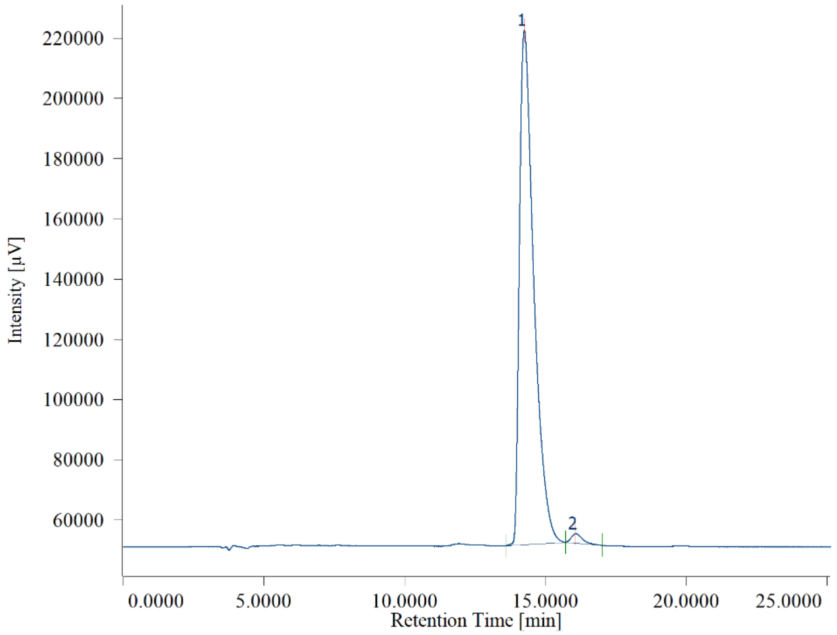

Peak Information

#	tR [min]	Area [µV·sec]	Height [µV]	Area%	Height%
1	14.232	6046497	170498	98.586	98.214
2	16.044	86698	3100	1.414	1.786

Figure S52. Chromatogram of optically active 1-phenylpropan-1-ol (**P14**)

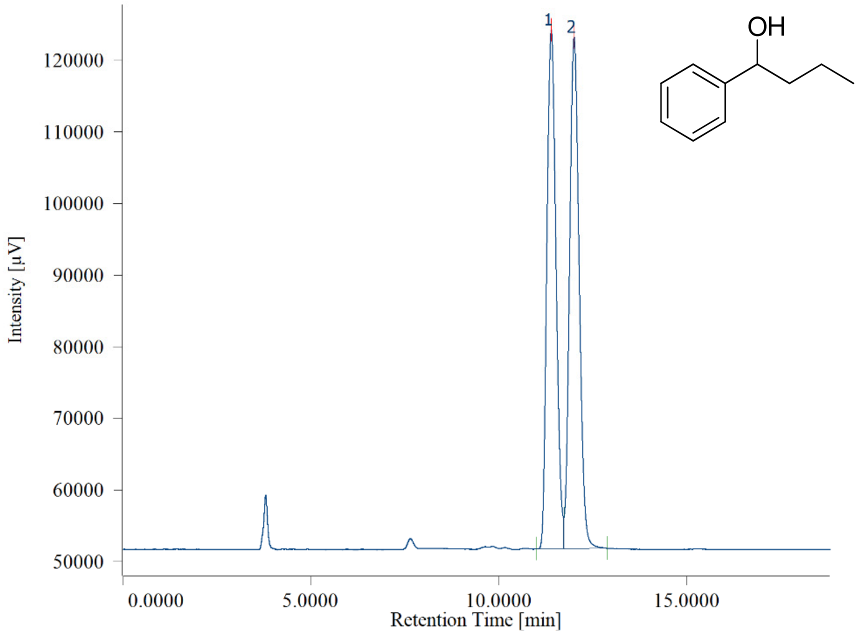

Peak Information

#	tR [min]	Area [µV·sec]	Height [µV]	Area%	Height%
1	11.391	1223538	72395	49.105	50.348
2	11.999	1268132	71395	50.895	49.652

Figure S53. Chromatogram of racemic 1-phenylbutan-1-ol

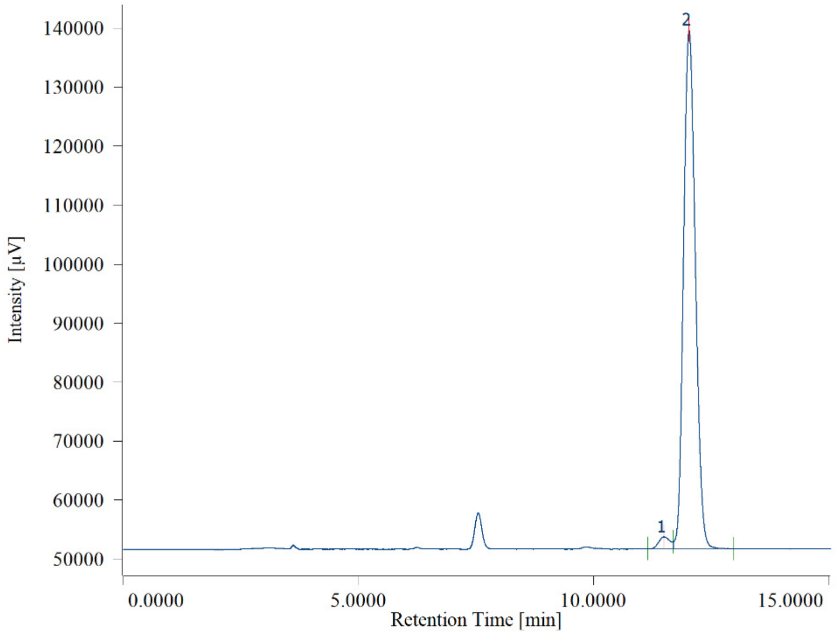

Peak Information

#	tR [min]	Area [µV·sec]	Height [µV]	Area%	Height%
1	11.490	34213	2045	2.183	2.275
2	12.016	1532761	87813	97.817	97.725

Figure S54. Chromatogram of optically active 1-phenylbutan-1-ol (**P15**)

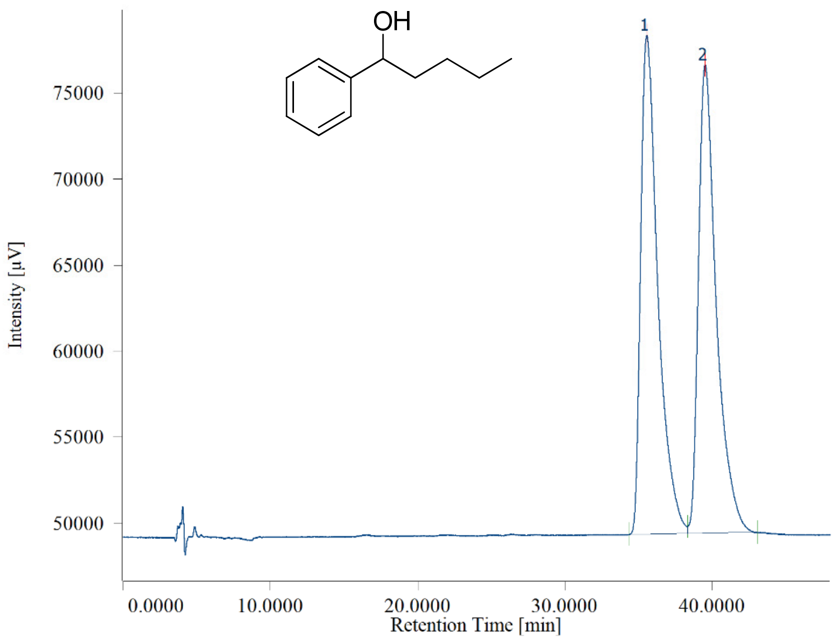

Peak Information

#	tR [min]	Area [µV·sec]	Height [µV]	Area%	Height%
1	35.544	2297561	28928	50.018	51.574
2	39.489	2295884	27162	49.982	48.426

Figure S55. Chromatogram of racemic 1-phenylpentan-1-ol

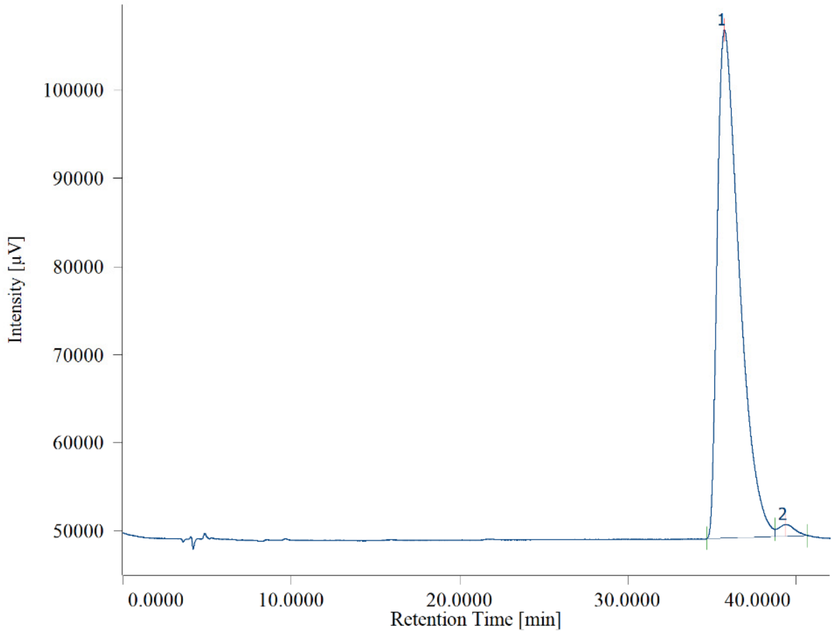

Peak Information

#	tR [min]	Area [µV·sec]	Height [µV]	Area%	Height%
1	35.694	5247875	57518	98.328	97.748
2	39.318	89232	1325	1.672	2.252

Figure S56. Chromatogram of optically active 1-phenylpentan-1-ol (**P16**)

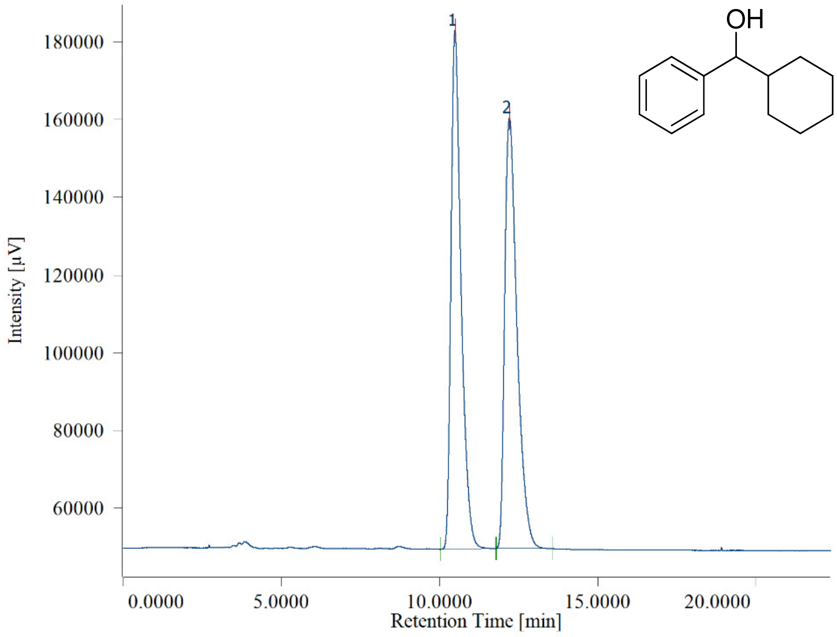

Peak Information

#	tR [min]	Area [µV·sec]	Height [µV]	Area%	Height%
1	10.477	2951674	133364	49.675	54.624
2	12.197	2990272	110786	50.325	45.376

Figure S57. Chromatogram of racemic cyclohexyl(phenyl)methanol

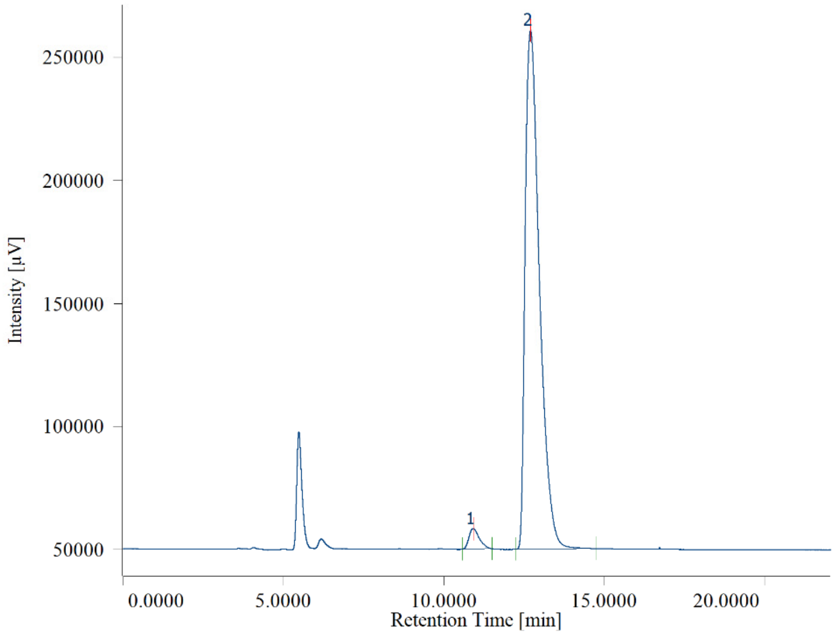

Peak Information

#	tR [min]	Area [µV·sec]	Height [µV]	Area%	Height%
1	10.901	185096	8380	2.760	3.826
2	12.690	6522459	210608	97.240	96.174

Figure S58. Chromatogram of optically active cyclohexyl(phenyl)methanol (**P17**)

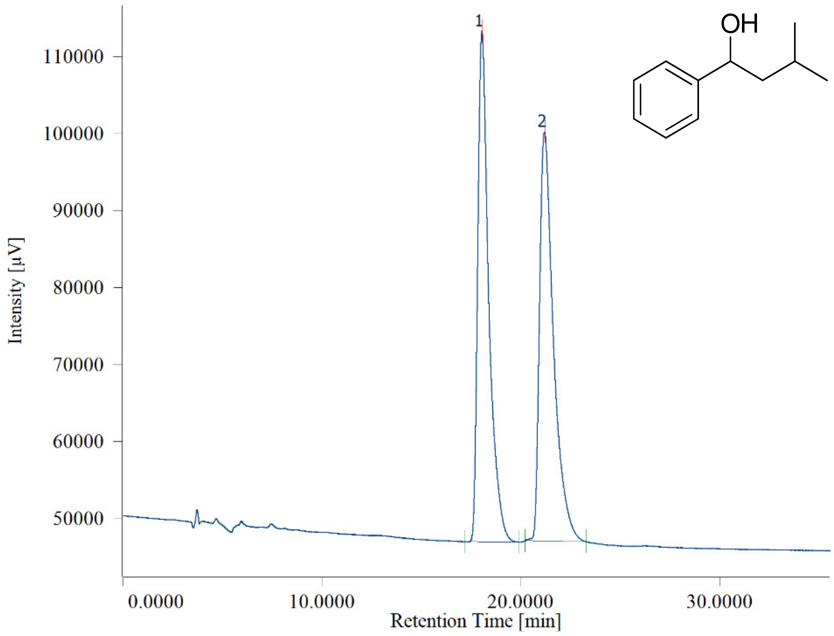

Peak Information

#	tR [min]	Area [µV·sec]	Height [µV]	Area%	Height%
1	18.026	2552020	66230	49.635	55.487
2	21.170	2589519	53131	50.365	44.513

Figure S59. Chromatogram of racemic 3-methyl-1-phenylbutan-1-ol

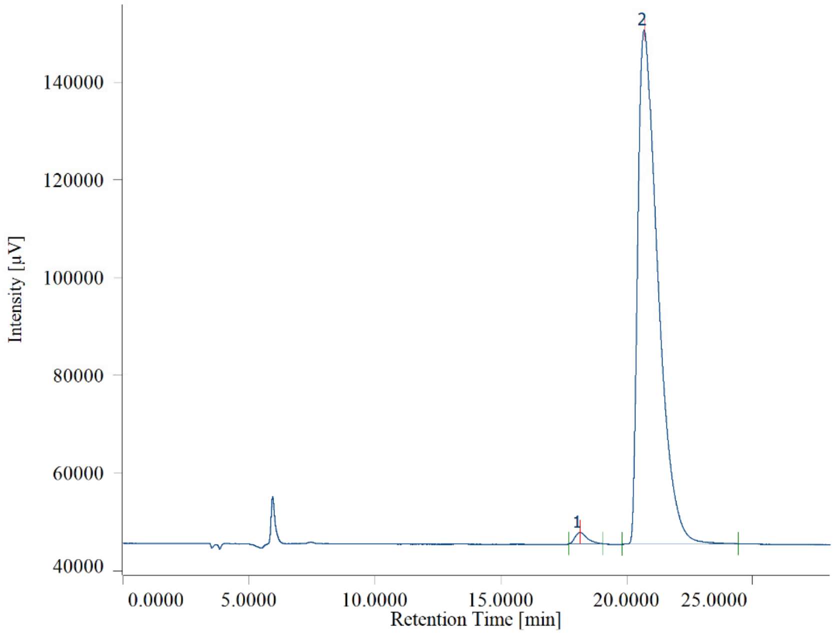

Peak Information

#	tR [min]	Area [µV·sec]	Height [µV]	Area%	Height%
1	18.130	77567	2341	1.339	2.182
2	20.679	5715701	104939	98.661	97.818

Figure S60. Chromatogram of optically active 3-methyl-1-phenylbutan-1-ol (**P18**)

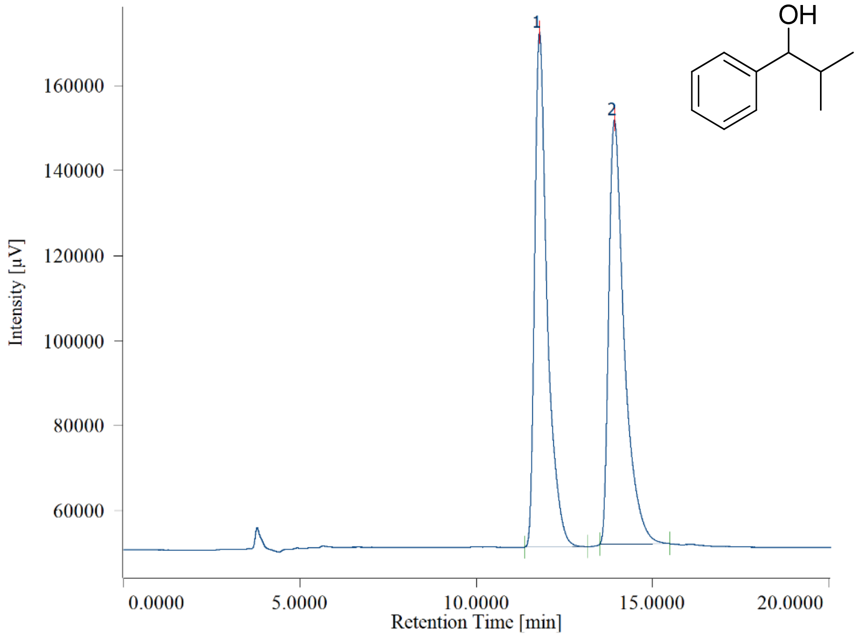

Peak Information

#	tR [min]	Area [µV·sec]	Height [µV]	Area%	Height%
1	11.787	2985552	120819	49.498	54.780
2	13.910	3046059	99735	50.502	45.220

Figure S61. Chromatogram of racemic 2-methyl-1-phenylpropan-1-ol

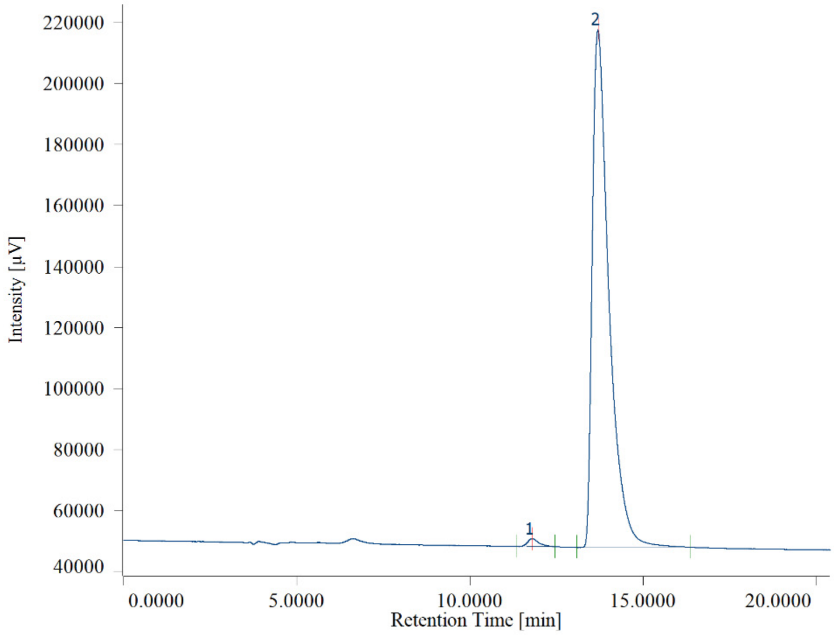

Peak Information

#	tR [min]	Area [µV·sec]	Height [µV]	Area%	Height%
1	11.782	55547	2498	0.974	1.453
2	13.682	5648765	169438	99.026	98.547

Figure S62. Chromatogram of optically active 2-methyl-1-phenylpropan-1-ol (**P19**)

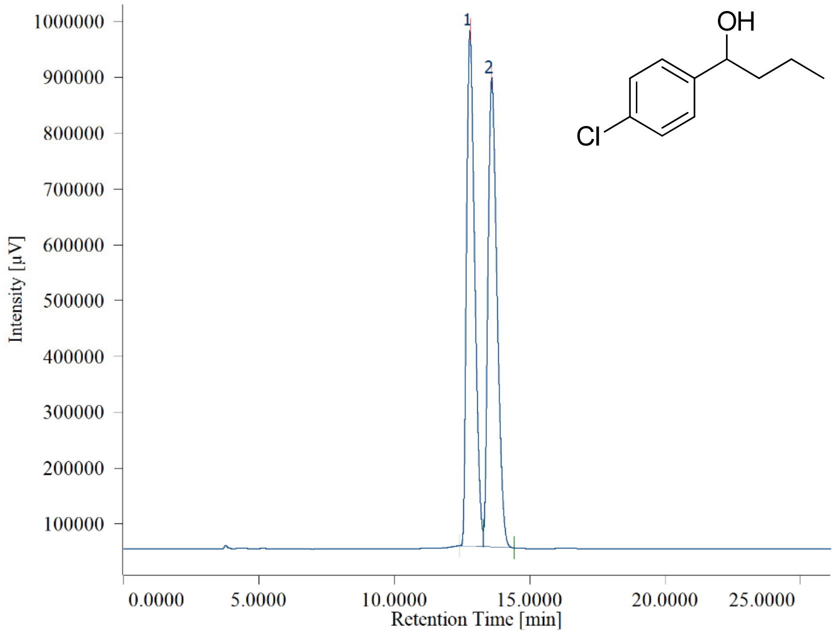

Peak Information

#	tR [min]	Area [µV·sec]	Height [µV]	Area%	Height%
1	12.787	19298156	923707	49.683	52.365
2	13.589	19544237	840283	50.317	47.635

Figure S63. Chromatogram of racemic 1-(4-chlorophenyl)butan-1-ol

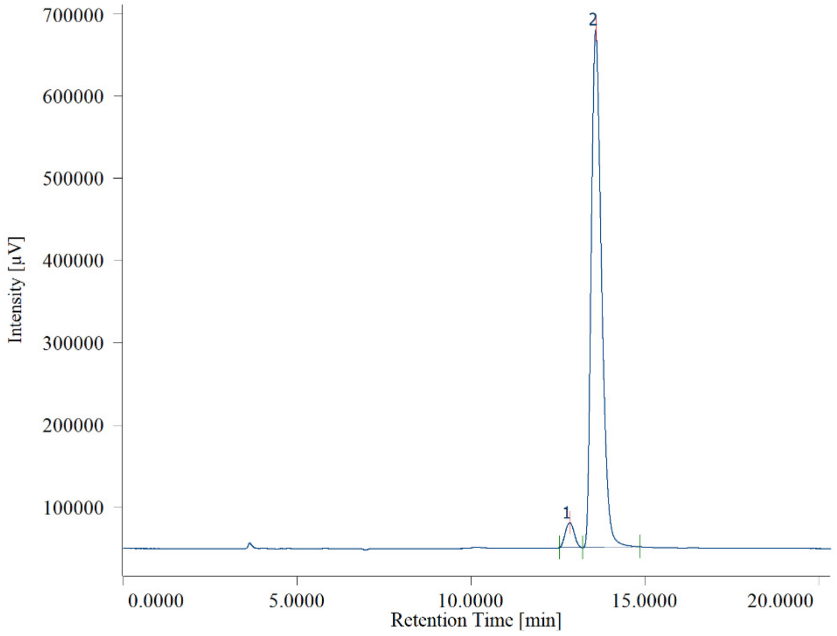

Peak Information

#	tR [min]	Area [µV·sec]	Height [µV]	Area%	Height%
1	12.828	539452	30239	4.016	4.595
2	13.576	12891642	627773	95.984	95.405

Figure S64. Chromatogram of optically active 1-(4-chlorophenyl)butan-1-ol (**P20**)

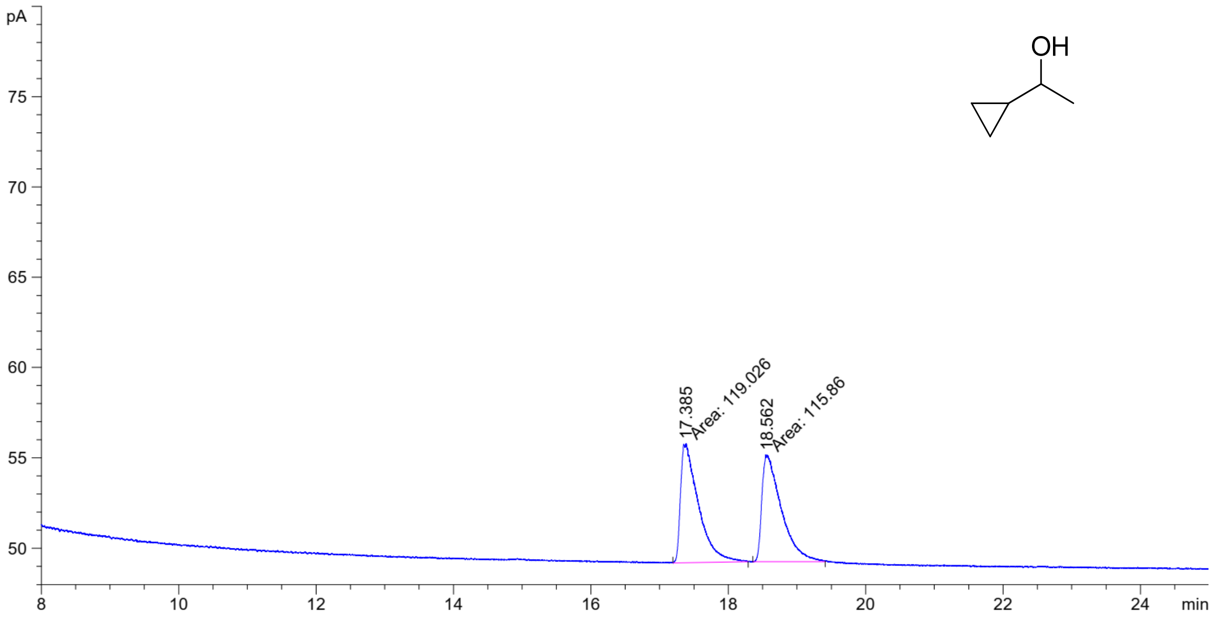

Peak #	RetTime [min]	Type	Width [min]	Area [pA*s]	Height [pA]	Area %
1	17.385	MM	0.3007	119.02570	6.59765	50.67383
2	18.562	MM	0.3273	115.86024	5.89938	49.32617
Totals :				234.88594	12.49703	

Figure S65. Chromatogram of racemic 1-cyclopropylethanol

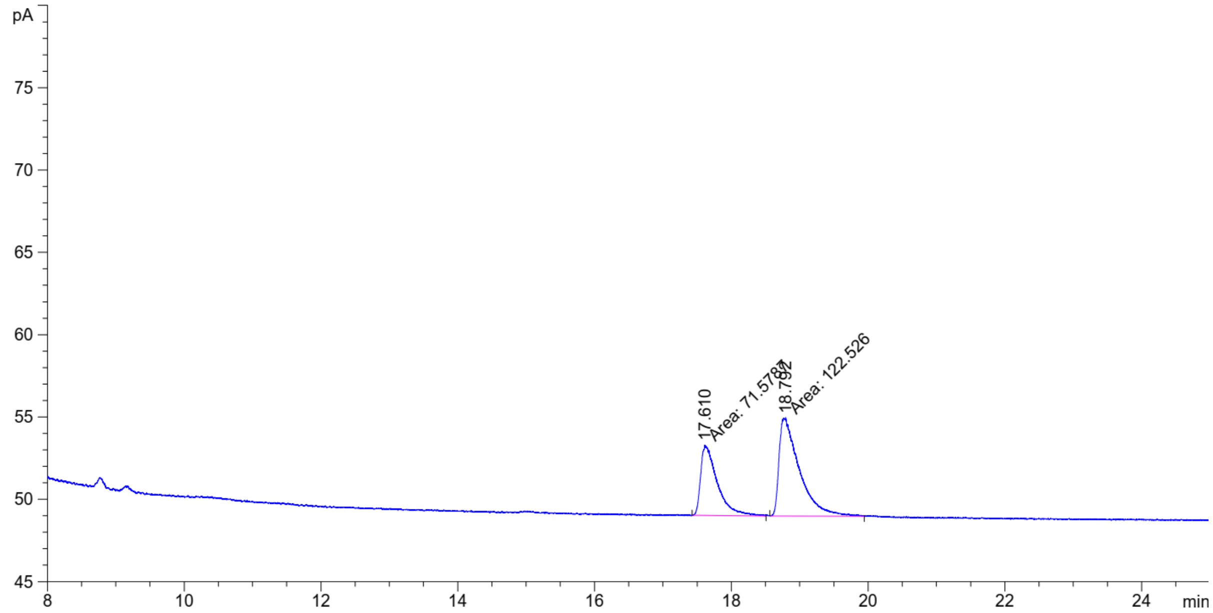

Peak #	RetTime [min]	Type	Width [min]	Area [pA*s]	Height [pA]	Area %
1	17.610	MM	0.2796	71.57865	4.26663	36.87625
2	18.792	MM	0.3422	122.52636	5.96798	63.12375
Totals :				194.10501	10.23461	

Figure S66. Chromatogram of optically active 1-cyclopropylethanol (**P21**)

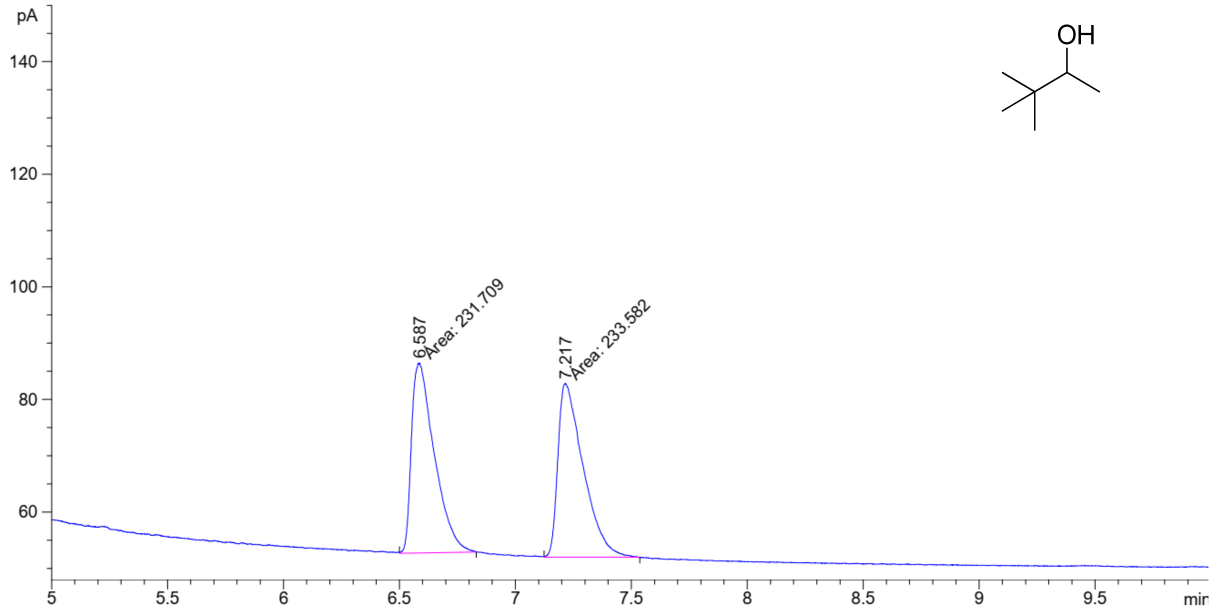

Peak #	RetTime [min]	Type	Width [min]	Area [pA*s]	Height [pA]	Area %
1	6.587	MM	0.1144	231.70859	33.76299	49.79863
2	7.217	MM	0.1263	233.58247	30.82583	50.20137
Totals :				465.29106	64.58882	

Figure S67. Chromatogram of racemic 3,3-dimethylbutan-2-ol

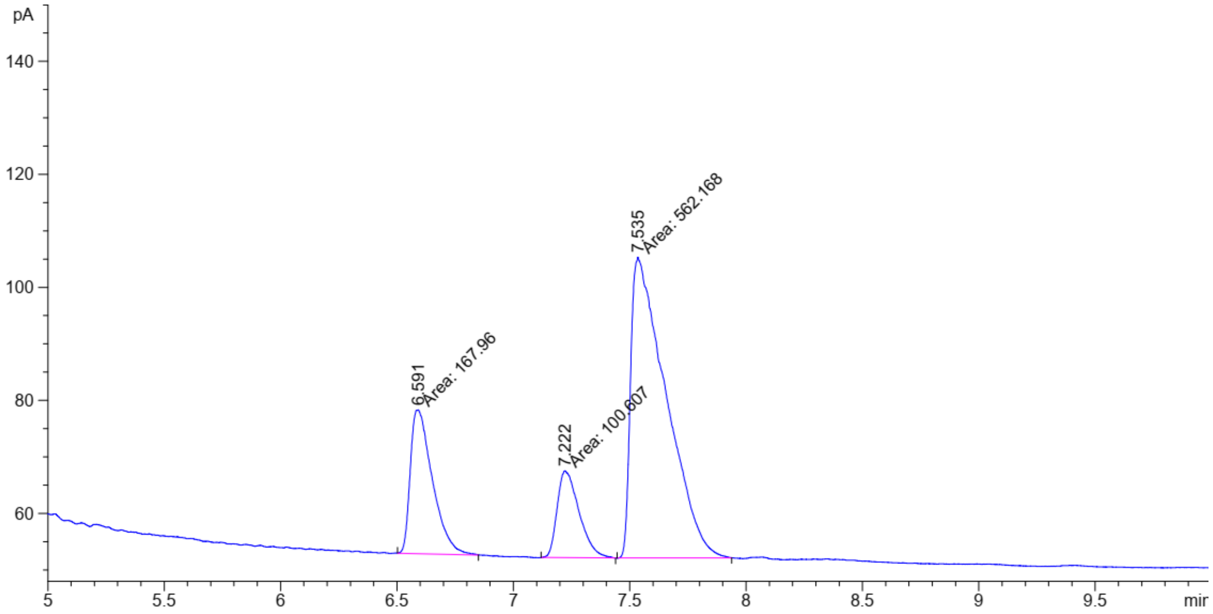

Peak #	RetTime [min]	Type	Width [min]	Area [pA*s]	Height [pA]	Area %
1	6.591	MM	0.1100	167.95955	25.45743	20.21820
2	7.222	MM	0.1092	100.60707	15.36175	12.11062
3	7.535	MM	0.1760	562.16785	53.24131	67.67118
Totals :				830.73447	94.06049	

Figure S68. Chromatogram of optically active 3,3-dimethylbutan-2-ol (**P22**)

4. DFT computational studies

DFT calculations were performed using the Gaussian 09 package[12]. All the structures are optimized using the CAM-B3LYP functional combination[13] with the SDD basis set and pseudopotential[14] for Ir as well as the 6-31G* basis set[15] for the rest of the atoms. All the structures were optimized in ethanol using the CPCM implicit solvation model.[16]

Table S1. Relative enthalpies of Ir-complexes **A-H**

Complex	Structure of the complex	Relative enthalpy (kJ/mol)
**A**	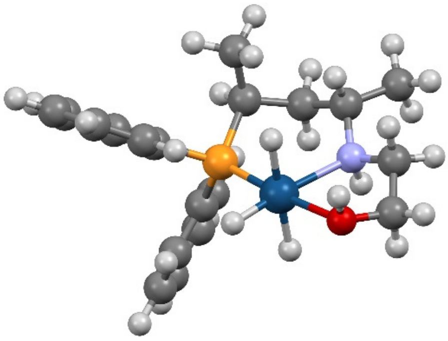	0
**B**	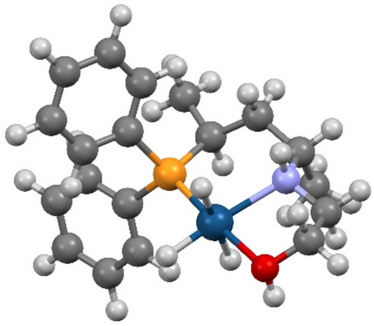	12
**C**	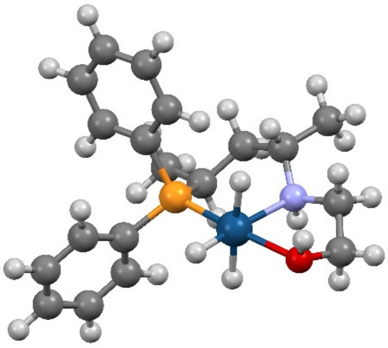	12
**D**	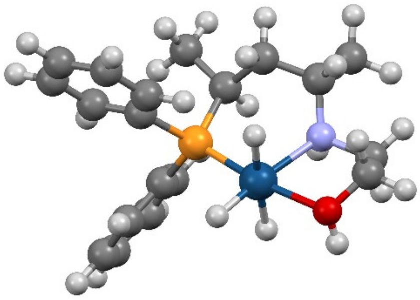	15

**E**	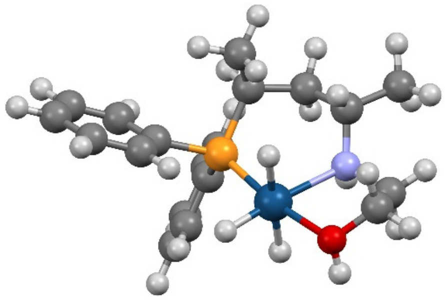	16
**F**	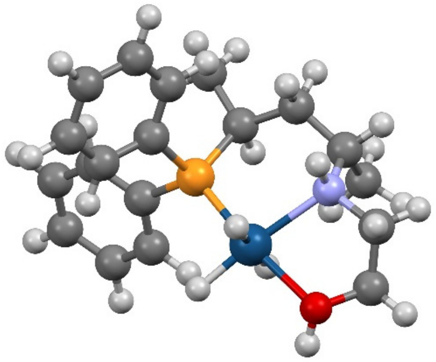	23
**G**	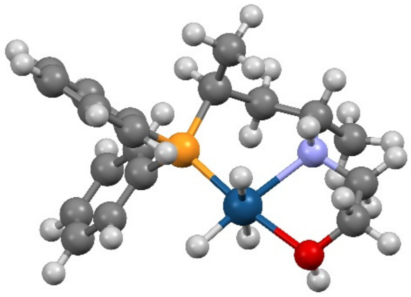	26
**H**	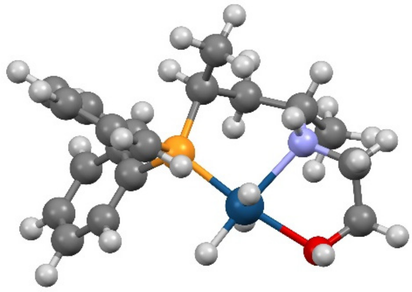	28

Cartesian coordinates of optimized structures

A

```
 C      3.071506    -1.290137    -0.726650
 C      2.130658    -1.196403     0.302853
 C      2.189265    -2.122386     1.348841
 C      3.175446    -3.102405     1.375762
 C      4.111082    -3.181624     0.348818
 C      4.053549    -2.275565    -0.704765
 P      0.756198     0.020119     0.195765
Ir     -1.129713    -0.764549    -0.665048
 O     -3.130033    -1.499955    -1.301949
 C     -4.073213    -1.170505    -0.262453
 C     -3.790459     0.201397     0.315304
 N     -2.418938     0.253613     0.864228
```

C	-1.956824	1.630041	1.199606
C	-3.013002	2.474622	1.915620
C	1.577892	1.518752	-0.497494
C	0.888043	2.365480	-1.367267
C	1.475397	3.537363	-1.836673
C	2.764217	3.878629	-1.444290
C	3.465135	3.042034	-0.579928
C	2.877784	1.873329	-0.110182
C	0.378767	0.502358	1.956515
C	1.579071	0.896085	2.823085
C	-0.682014	1.618641	2.068367
H	-0.053998	-0.417177	2.365683
H	-0.991273	1.624631	3.121347
H	-0.188866	2.582780	1.900670
H	-1.733727	2.089075	0.233734
H	2.356018	0.128680	2.830566
H	2.026131	1.831957	2.476790
H	1.249958	1.049163	3.856345
H	-3.882225	2.678378	1.286199
H	-3.353658	1.982825	2.833771
H	-2.576460	3.437978	2.195411
H	-3.864891	0.958126	-0.471150
H	-4.543473	0.426294	1.078119
H	-5.087367	-1.222193	-0.665295
H	-3.949889	-1.950361	0.491668
H	1.458772	-2.091867	2.150570
H	3.209591	-3.806862	2.200893
H	4.775709	-2.331668	-1.513220
H	3.044695	-0.587296	-1.553165
H	3.445734	1.229083	0.551823
H	4.474232	3.297261	-0.272005
H	3.224689	4.790069	-1.812702
H	0.923302	4.180510	-2.515140
H	-0.111589	2.088825	-1.684084
H	4.879610	-3.947835	0.368850
H	-2.408095	-0.304799	1.717447
H	-0.322668	-1.538621	-1.819805
H	-1.388192	0.513760	-1.722291
H	-0.982582	-2.106436	0.355360
H	-3.345493	-0.989693	-2.098616

B

C	2.504921	1.612602	-1.169287
C	1.738613	1.495353	-0.003546
C	1.664260	2.595390	0.854039
C	2.347581	3.773575	0.564396
C	3.111109	3.873587	-0.592530
C	3.186030	2.788062	-1.460995
P	0.746655	-0.035916	0.248840
C	0.463097	-0.260235	2.084748
C	-0.753430	0.511734	2.639073
C	-2.159609	0.017534	2.268981
C	-2.346509	-1.478917	2.493880
C	1.982170	-1.365344	-0.088026
C	3.346022	-1.189641	0.177482
C	4.249266	-2.227960	-0.022393
C	3.805037	-3.460958	-0.489918
C	2.453258	-3.647970	-0.756779
C	1.549989	-2.607682	-0.559161

Ir	-1.179153	-0.081744	-0.847743
N	-2.506642	0.437710	0.883051
C	-3.891182	0.102608	0.495593
C	-4.138157	0.522037	-0.938964
O	-3.189281	-0.113901	-1.818077
C	1.693449	-0.047569	2.975230
H	-2.851915	0.544786	2.941197
H	0.222501	-1.328647	2.127246
H	-0.692124	0.452210	3.731768
H	-0.683110	1.579261	2.400855
H	2.574973	0.775924	-1.857573
H	3.776891	2.855439	-2.369119
H	3.644025	4.791817	-0.818181
H	2.279551	4.613541	1.248606
H	1.066600	2.552957	1.756247
H	0.497171	-2.746567	-0.776326
H	2.097821	-4.605034	-1.126301
H	4.510831	-4.270468	-0.648208
H	5.302813	-2.070329	0.186230
H	3.714674	-0.233881	0.532979
H	1.465029	-0.383010	3.992108
H	2.551987	-0.623255	2.623372
H	1.991550	1.001850	3.032777
H	-3.403550	-1.750819	2.543113
H	-1.878008	-2.060141	1.696686
H	-1.894906	-1.761457	3.449361
H	-4.618542	0.591426	1.156786
H	-5.152402	0.266616	-1.254587
H	-3.979858	1.593355	-1.076496
H	-2.442967	1.453776	0.881997
H	-4.025186	-0.977421	0.581447
H	-1.356733	-1.733244	-0.588006
H	-0.374064	-0.406016	-2.199480
H	-1.104911	1.573105	-1.185708
H	-3.417923	-1.052710	-1.906840

C

C	3.071506	-1.290137	-0.726650
C	2.130658	-1.196403	0.302853
C	2.189265	-2.122386	1.348841
C	3.175446	-3.102405	1.375762
C	4.111082	-3.181624	0.348818
C	4.053549	-2.275565	-0.704765
P	0.756198	0.020119	0.195765
Ir	-1.129713	-0.764549	-0.665048
O	-3.130033	-1.499955	-1.301949
C	-4.073213	-1.170505	-0.262453
C	-3.790459	0.201397	0.315304
N	-2.418938	0.253613	0.864228
C	-1.956824	1.630041	1.199606
C	-3.013002	2.474622	1.915620
C	1.577892	1.518752	-0.497494
C	0.888043	2.365480	-1.367267
C	1.475397	3.537363	-1.836673
C	2.764217	3.878629	-1.444290
C	3.465135	3.042034	-0.579928
C	2.877784	1.873329	-0.110182
C	0.378767	0.502358	1.956515
C	1.579071	0.896085	2.823085

C	-0.682014	1.618641	2.068367
H	-0.053998	-0.417177	2.365683
H	-0.991273	1.624631	3.121347
H	-0.188866	2.582780	1.900670
H	-1.733727	2.089075	0.233734
H	2.356018	0.128680	2.830566
H	2.026131	1.831957	2.476790
H	1.249958	1.049163	3.856345
H	-3.882225	2.678378	1.286199
H	-3.353658	1.982825	2.833771
H	-2.576460	3.437978	2.195411
H	-3.864891	0.958126	-0.471150
H	-4.543473	0.426294	1.078119
H	-5.087367	-1.222193	-0.665295
H	-3.949889	-1.950361	0.491668
H	1.458772	-2.091867	2.150570
H	3.209591	-3.806862	2.200893
H	4.775709	-2.331668	-1.513220
H	3.044695	-0.587296	-1.553165
H	3.445734	1.229083	0.551823
H	4.474232	3.297261	-0.272005
H	3.224689	4.790069	-1.812702
H	0.923302	4.180510	-2.515140
H	-0.111589	2.088825	-1.684084
H	4.879610	-3.947835	0.368850
H	-2.408095	-0.304799	1.717447
H	-0.322668	-1.538621	-1.819805
H	-1.388192	0.513760	-1.722291
H	-0.982582	-2.106436	0.355360
H	-3.345493	-0.989693	-2.098616

D

C	2.550472	-1.731358	-1.027592
C	1.838485	-1.469037	0.149334
C	1.927805	-2.382591	1.201002
C	2.710321	-3.529324	1.081047
C	3.413297	-3.777596	-0.091187
C	3.331611	-2.873100	-1.147542
P	0.741307	0.005542	0.192031
Ir	-1.154860	-0.140560	-0.947960
O	-3.183604	-0.209613	-1.850861
C	-4.109197	0.197733	-0.823766
C	-3.805901	-0.515894	0.478131
N	-2.402839	-0.284805	0.909771
C	-2.268566	0.766002	1.962447
C	-2.757304	0.235165	3.311913
C	1.918518	1.377891	-0.175994
C	1.456623	2.575353	-0.728682
C	2.326702	3.638312	-0.953363
C	3.674051	3.518987	-0.632528
C	4.147460	2.331572	-0.082140
C	3.277544	1.271564	0.145906
C	0.304144	0.268215	1.992806
C	1.480866	0.664630	2.888333
C	-0.832726	1.310951	2.076648
H	-0.074295	-0.700583	2.340813
H	-0.762829	1.814378	3.047097
H	-0.677024	2.083929	1.320002
H	-2.911255	1.593344	1.646057

H	2.324889	-0.023935	2.795375
H	1.838898	1.668868	2.646106
H	1.160662	0.664662	3.935164
H	-3.763935	-0.187444	3.251095
H	-2.086732	-0.545973	3.688936
H	-2.779494	1.041209	4.050154
H	1.388703	-2.217627	2.127144
H	2.768510	-4.226672	1.910810
H	3.876955	-3.058049	-2.067693
H	2.494713	-1.035341	-1.859543
H	3.666953	0.353883	0.572151
H	5.198140	2.227555	0.169983
H	4.354326	4.345567	-0.812803
H	1.948457	4.559350	-1.386255
H	0.408588	2.663215	-0.991418
H	4.022779	-4.670897	-0.183849
H	-2.084042	-1.157085	1.322382
H	-4.519019	-0.189618	1.241453
H	-3.943845	-1.591921	0.339090
H	-5.130779	-0.010416	-1.152194
H	-3.970784	1.275565	-0.732973
H	-0.367985	-0.088691	-2.349461
H	-1.305673	1.527349	-0.976458
H	-1.127798	-1.835677	-0.996006
H	-3.359007	-1.133259	-2.092816

E

C	-2.049603	-2.469020	-0.118350
C	-2.202829	-1.139467	0.277600
C	-3.449788	-0.714008	0.749681
C	-4.517730	-1.600464	0.824411
C	-4.355183	-2.924271	0.424450
C	-3.120292	-3.356044	-0.046861
P	-0.775890	0.022680	0.249283
C	-0.407084	0.260049	2.060569
C	-0.308432	-1.069336	2.815432
C	-1.606052	1.586439	-0.273288
C	-1.858540	2.667147	0.573947
C	-2.494207	3.813147	0.097239
C	-2.896735	3.889698	-1.229209
C	-2.658785	2.814216	-2.082935
C	-2.015431	1.679251	-1.609609
Ir	1.071303	-0.544046	-0.829498
N	2.402472	0.772871	0.435798
C	2.229266	0.631334	1.912510
C	0.830752	1.147119	2.304011
O	3.054260	-1.140445	-1.625769
C	4.022411	-0.797572	-0.614640
C	3.774455	0.607102	-0.108589
C	3.275714	1.403545	2.722466
H	-1.263514	0.798209	2.482258
H	2.314206	-0.434525	2.136577
H	-1.260276	-1.604959	2.799472
H	-0.049389	-0.874740	3.861837
H	0.454418	-1.722615	2.386915
H	3.266716	2.469139	2.465912
H	3.049072	1.312551	3.788010
H	4.289170	1.023126	2.580944
H	0.853242	1.340124	3.381671

H	0.691604	2.130332	1.835100
H	-1.821969	0.854392	-2.288170
H	-2.971597	2.862601	-3.121362
H	-3.393766	4.780967	-1.599085
H	-2.675310	4.643734	0.772237
H	-1.568329	2.637839	1.617636
H	-1.084945	-2.799538	-0.484898
H	-2.987462	-4.386241	-0.362764
H	-5.190731	-3.615271	0.478330
H	-5.479428	-1.255540	1.191266
H	-3.594567	0.317723	1.055161
H	2.123170	1.723705	0.206812
H	3.872764	1.303515	-0.946692
H	4.543778	0.871451	0.619292
H	3.888125	-1.543103	0.169741
H	5.028868	-0.886671	-1.031676
H	0.257665	-1.511165	-1.810755
H	1.019807	0.686859	-1.994618
H	1.213881	-1.822261	0.245834
H	3.219956	-0.608001	-2.420313

F

C	1.501689	-2.670924	-0.323167
C	1.955689	-1.400936	0.042034
C	3.320285	-1.229190	0.304863
C	4.203997	-2.298312	0.204493
C	3.738442	-3.558489	-0.157740
C	2.385139	-3.741901	-0.419943
P	0.745973	-0.022217	0.246488
Ir	-1.166053	-0.135228	-0.866013
O	-3.221541	-0.332887	-1.717427
C	-4.127750	-0.409786	-0.596750
C	-3.830632	0.782817	0.284400
N	-2.414863	0.809311	0.734746
C	-2.201212	0.434992	2.159573
C	-2.604754	-0.999930	2.468407
C	1.782352	1.460442	-0.098641
C	2.535026	1.478610	-1.279816
C	3.251931	2.607934	-1.653264
C	3.227283	3.747732	-0.853424
C	2.480396	3.745871	0.317925
C	1.762157	2.611959	0.691079
C	0.401954	-0.107864	2.086508
C	1.627160	0.079636	2.990058
C	-0.764305	0.776174	2.580326
H	-2.848055	1.094084	2.756996
H	0.085942	-1.153185	2.182832
H	-0.752304	0.721190	3.674971
H	-0.578243	1.830241	2.343457
H	2.564412	0.599099	-1.915950
H	3.830668	2.597826	-2.571579
H	3.786901	4.631196	-1.143842
H	2.452828	4.628099	0.949706
H	1.183841	2.646460	1.605935
H	0.448200	-2.807934	-0.536906
H	2.013280	-4.720727	-0.707042
H	4.428796	-4.392469	-0.237667
H	5.258787	-2.143745	0.409058
H	3.704454	-0.253754	0.581972

H	1.357572	-0.167106	4.022125
H	2.450104	-0.575447	2.697771
H	1.996116	1.108253	2.982256
H	-3.680615	-1.146898	2.337008
H	-2.080930	-1.709880	1.826513
H	-2.372334	-1.222917	3.514077
H	-3.963641	-1.351663	-0.068587
H	-5.156187	-0.361885	-0.964907
H	-4.012865	1.692025	-0.294609
H	-4.514886	0.790117	1.139275
H	-2.119612	1.778522	0.666169
H	-1.032082	1.417563	-1.540697
H	-1.388468	-1.686202	-0.276544
H	-0.403667	-0.780213	-2.124926
H	-3.254243	-1.169942	-2.206521

G

C	-2.105839	2.523860	0.648626
C	-1.784915	1.478138	-0.218511
C	-2.261344	1.532360	-1.534638
C	-3.035206	2.599314	-1.969929
C	-3.343484	3.640740	-1.096956
C	-2.876536	3.599963	0.210056
P	-0.772264	0.009292	0.248262
Ir	1.115112	-0.272322	-0.869843
O	3.101871	-0.618523	-1.799799
C	4.002152	-1.032716	-0.753056
C	3.858583	-0.129235	0.454156
N	2.461653	-0.134367	0.943789
C	2.208922	0.797475	2.099015
C	3.071139	2.057484	2.055078
C	-2.070789	-1.299968	0.252968
C	-3.330027	-1.020760	0.799327
C	-4.308866	-2.004670	0.864235
C	-4.047023	-3.282714	0.376668
C	-2.801843	-3.569255	-0.170301
C	-1.818555	-2.584526	-0.229205
C	-0.398091	0.228960	2.057769
C	0.742595	1.250305	2.228989
C	-0.156491	-1.095083	2.790264
H	4.120731	0.887259	0.158351
H	0.585954	2.083946	1.536523
H	0.659012	1.674478	3.236641
H	-3.554650	-0.025959	1.171785
H	-5.279122	-1.771831	1.291802
H	-4.813330	-4.050319	0.422584
H	-2.590950	-4.562693	-0.554333
H	-0.843096	-2.807273	-0.645691
H	-1.763290	2.521744	1.676959
H	-3.111783	4.404688	0.899551
H	-3.945132	4.477908	-1.436542
H	-3.396518	2.620335	-2.993400
H	-2.016176	0.732596	-2.227144
H	0.623799	-1.704943	2.326317
H	-1.065674	-1.699819	2.815409
H	0.142319	-0.896335	3.824683
H	4.135653	1.848415	2.177150
H	2.771025	2.714611	2.875676
H	2.921958	2.606283	1.119437

H	-1.293529	0.672671	2.506168
H	2.480972	0.240654	3.004230
H	3.720293	-2.062640	-0.529170
H	5.028160	-1.013030	-1.128821
H	4.561278	-0.448179	1.234243
H	2.300841	-1.067466	1.310332
H	1.059781	-1.928742	-0.593401
H	1.280520	1.372339	-1.229128
H	0.286179	-0.468355	-2.227913
H	3.402279	0.229811	-2.163785

H

C	-2.025041	2.509718	0.860913
C	-1.772008	1.511801	-0.081351
C	-2.287350	1.667049	-1.374296
C	-3.037257	2.784866	-1.713334
C	-3.280446	3.776723	-0.765273
C	-2.771922	3.636429	0.519143
P	-0.779329	-0.005786	0.249960
Ir	1.114065	-0.172036	-0.888176
O	3.152760	-0.278026	-1.779139
C	4.117609	-0.095561	-0.721892
C	3.701951	-0.867908	0.517128
N	2.330058	-0.503337	0.951331
C	2.243285	0.442732	2.106146
C	3.170108	1.644923	1.963799
C	-2.084611	-1.300207	0.134196
C	-3.345922	-1.069366	0.697874
C	-4.326276	-2.053162	0.664551
C	-4.062917	-3.280648	0.060812
C	-2.815295	-3.517843	-0.503815
C	-1.830279	-2.533621	-0.465092
C	-0.402206	0.049030	2.080130
C	0.810409	0.968076	2.338329
C	-0.298117	-1.335740	2.732748
H	0.689584	1.875991	1.740253
H	0.772726	1.284744	3.387381
H	-3.569820	-0.113190	1.161225
H	-5.298945	-1.859996	1.106137
H	-4.830881	-4.047342	0.030152
H	-2.604372	-4.471299	-0.978469
H	-0.853393	-2.714701	-0.899985
H	-1.645085	2.431841	1.872948
H	-2.955454	4.402435	1.266065
H	-3.863539	4.653307	-1.029350
H	-3.430501	2.884641	-2.720233
H	-2.092436	0.906593	-2.124464
H	0.390075	-2.016726	2.223371
H	-1.271556	-1.830110	2.750777
H	0.044215	-1.229422	3.767186
H	4.226707	1.368165	1.944076
H	3.024445	2.305995	2.822854
H	2.928694	2.208386	1.058196
H	-1.263471	0.535866	2.549165
H	2.557797	-0.125139	2.992132
H	5.100257	-0.422123	-1.071690
H	4.138862	0.978396	-0.543616
H	4.434218	-0.718147	1.316990
H	3.693605	-1.935366	0.278614

H	1.919338	-1.367444	1.284236
H	3.235125	-1.176957	-2.134956
H	1.055923	-1.851721	-1.069978
H	0.335560	0.003697	-2.284457
H	1.284481	1.496968	-0.784942

[1] Xie, J.-B.; Xie, J.-H.; Liu, X.-Y.; Zhang, Q.-Q.; Zhou, Q.-L. *Chem. Asian J.* **2011**, *6*, 899.

[2] Wu, W.; Liu, S.; Duan, M.; Tan, X.; Chen, C.; Xie, Y.; Lan, Y.; Dong, X.-Q.; Zhang, X. *Org. Lett.* **2016**, *18*, 2938.

[3] Zhang, L.; Tang, Y.; Han, Z.; Ding, K. *Angew. Chem. Int. Ed.* **2019**, *58*, 4973.

[4] Liu, W.-P.; Yuan, M.-L.; Yang, X.-H.; Li, K.; Xie, J.-H.; Zhou, Q.-L. *Chem. Commun.* **2015**, *51*, 6123.

[5] More, G. V.; Badgujara, K. C.; Bhanage, B. M. *RSC Adv.* **2015**, *5*, 4592.

[6] Császár, Z.; Kovács, R.; Fonyó, M.; Simon, J.; Bényei, A.; Lendvay, G.; Bakos, J.; Farkas, G. *Mol. Catal.* **2022**, 529, 112531.

[7] Qin, C.; Hou, C.-J.; Liu, H.; Liu, Y.-J.; Huang, D.-Z.; Hu, X.-P. *Tetrahedron Lett.* **2018**, *59*, 719.

[8] Contente, M. L.; Molinari, F.; Zambelli, P.; De Vitis, V.; Gandolfi, R.; Pinto, A.; Romano, D. *Tetrahedron Lett.* **2014**, *55*, 7051.

[9] Collados, J. F.; Ortiz, P.; Pérez, J. M.; Xia, Y.; Koenis, M. A. J.; Buma, W. J.; Nicu, V. P.; Harutyunyan, S. R. *Eur. J. Org. Chem.* **2018**, *2018*, 3900.

[10] Komagawa, H.; Maejima, Y.; Nagano, T. *Synlett*, **2016**, *27*, 789.

[11] Yu, J.; Long, J.; Yang, Y.; Wu, W.; Xue, P.; Chung, L. W.; Dong, X-Q.; Zhang, X. *Org. Lett.* **2017**, *19*, 690.

[12] M. J. Frisch, G. W. Trucks, H. B. Schlegel, G. E. Scuseria, M. A. Robb, J. R. Cheeseman, G. Scalmani, V. Barone, B. Mennucci, G. A. Petersson, H. Nakatsuji, M. Caricato, X. Li, H. P. Hratchian, A. F. Izmaylov, J. Bloino, G. Zheng, J. L. Sonnenberg, M. Hada, M. Ehara, K. Toyota, R. Fukuda, J. Hasegawa, M. Ishida, T. Nakajima, Y. Honda, O. Kitao, H. Nakai, T. Vreven, J. A. Montgomery, Jr., J. E. Peralta, F. Ogliaro, M. Bearpark, J. J. Heyd, E. Brothers, K. N. Kudin, V. N. Staroverov, R. Kobayashi, J. Normand, K. Raghavachari, A. Rendell, J. C. Burant, S. S. Iyengar, J. Tomasi, M. Cossi, N. Rega, J. M. Millam, M. Klene, J. E. Knox, J. B. Cross, V. Bakken, C. Adamo, J. Jaramillo, R. Gomperts, R. E. Stratmann, O. Yazyev, A. J. Austin, R. Cammi, C. Pomelli, J. W. Ochterski, R. L. Martin, K. Morokuma, V. G. Zakrzewski, G. A. Voth, P. Salvador, J. J. Dannenberg, S. Dapprich, A. D. Daniels, O. Farkas, J. B. Foresman, J. V. Ortiz, J. Cioslowski, and D. J. Fox, Gaussian, Inc., Wallingford CT, 2009.

[13] T. Yanai, D. Tew, and N. Handy *Chem. Phys. Lett.* **2004**, *393*, 51-57.

[14] D. Andrae, U. Häußermann, M. Dolg, H. Stoll, H. Preuß, *Theor. Chim. Acta* **1990**, *77*, 123-141.

[15] a) W. J. Hehre, R. Ditchfield, J. A. Pople, *J. Chem. Phys.* **1972**, *56*, 2257-2261. b) P. C. Hariharan, J. A. Pople, *Theor. Chim. Acta* **1973**, *28*, 213-222. c) M. M. Francl, W. J. Pietro, W. J. Hehre, J. S. Binkley, M. S. Gordon, D. J. DeFrees, J. A. Pople *J. Chem. Phys.* **1982**, *77*, 3654-3665. d) M. S. Gordon, J. S. Binkley, J. A. Pople, W. J. Pietro, W. J. Hehre *J. Am. Chem. Soc.* **1982**, *104*, 2797-2803.

[16] Tomasi, J.; Mennucci, B.; Cammi, R. *Chem. Rev.* **2005**, *105*, 2999.
